# Metal–organic layers stabilize earth-abundant metal–terpyridine diradical complexes for catalytic C–H activation†
†Electronic supplementary information (ESI) available. See DOI: 10.1039/c7sc03537c


**DOI:** 10.1039/c7sc03537c

**Published:** 2017-10-30

**Authors:** Zekai Lin, Nathan C. Thacker, Takahiro Sawano, Tasha Drake, Pengfei Ji, Guangxu Lan, Lingyun Cao, Shubin Liu, Cheng Wang, Wenbin Lin

**Affiliations:** a Department of Chemistry , University of Chicago , 929 E. 57th St. , Chicago , Illinois 60637 , USA . Email: wenbinlin@uchicago.edu; b Collaborative Innovation Center of Chemistry for Energy Materials , State Key Laboratory of Physical Chemistry of Solid Surfaces , Department of Chemistry , College of Chemistry and Chemical Engineering , Xiamen University , Xiamen 361005 , PR China; c Research Computing Center , University of North Carolina , Chapel Hill , North Carolina 27599-3420 , USA

## Abstract

We report the synthesis of a terpyridine-based metal–organic layer (TPY-MOL) and its metalation with CoCl_2_ and FeBr_2_ to afford CoCl_2_·TPY-MOL and FeBr_2_·TPY-MOL, respectively. Upon activation with NaEt_3_BH, CoCl_2_·TPY-MOL catalyzed benzylic C–H borylation of methylarenes whereas FeBr_2_·TPY-MOL catalyzed intramolecular C_sp^3^_–H amination of alkyl azides to afford pyrrolidines and piperidines. X-ray absorption near edge structure (XANES), extended X-ray absorption fine structure (EXAFS), X-ray photoelectron spectroscopy, UV-Vis-NIR spectroscopy, and electron paramagnetic spectroscopy (EPR) measurements as well as density functional theory (DFT) calculations identified M(THF)_2_·TPY-MOL (M = Co or Fe) as the active catalyst with a M^II^-(TPY˙˙)^2–^ electronic structure featuring divalent metals and TPY diradical dianions. We believe that site isolation stabilizes novel M^II^-(TPY˙˙)^2–^ (M = Co or Fe) species in the MOLs to endow them with unique and enhanced catalytic activities for C_sp^3^_–H borylation and intramolecular amination over their homogeneous counterparts. The MOL catalysts are also superior to their metal–organic framework analogs owing to the removal of diffusion barriers. Our work highlights the potential of MOLs as a novel 2D molecular material platform for designing single-site solid catalysts without diffusional constraints.

## Abstract 2

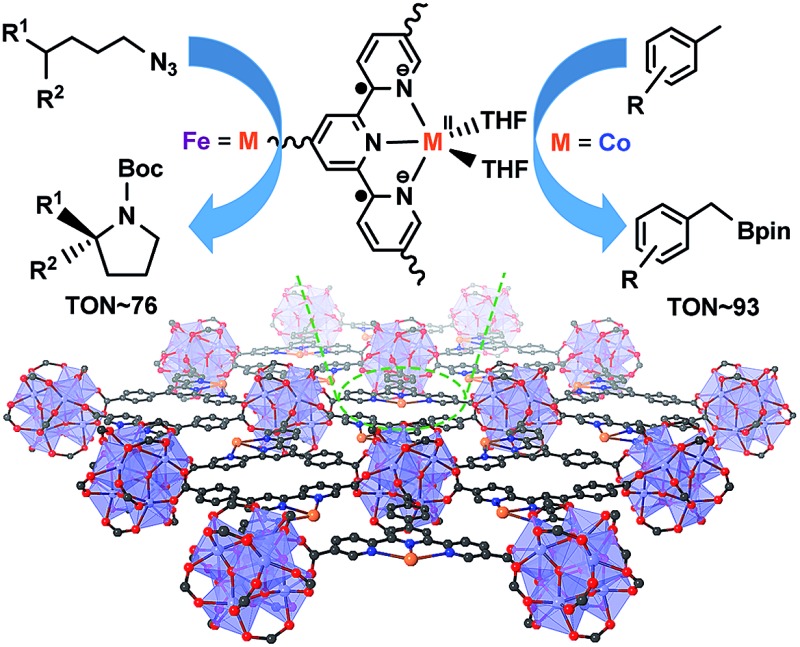
Metal–organic layers stabilize Fe^II^ or Co^II^-terpyridine diradical complexes to catalyze alkylazide C_sp^3^_–H amination and benzylic C–H borylation, respectively.

## Introduction

Over the past two decades, metal–organic frameworks (MOFs) have attracted great interest among scientists and engineers owing to their potential in various applications including gas storage and separation,^1^–^6^ heterogeneous catalysis,^7^–^16^ nonlinear optics,^17^,^18^ chemical sensing,^19^–^21^ biomedical imaging,^22^,^23^ and drug delivery.^24^,^25^ In particular, MOFs have provided an excellent platform for designing single-site solid catalysts for many important organic transformations.^26^–^32^ By shutting down intermolecular deactivation pathways *via* spatial isolation of active sites, MOFs have afforded turnover numbers (TONs) several orders of magnitude higher than their homogeneous analogs.^26^,^29^ The catalytic performance of MOFs is, however, still limited by the diffusion rates of large substrates and products within the 3D frameworks.^33^ Although many strategies have been devised to overcome this diffusion limitation of MOFs, for example, by elongating functional ligands^26^ or diluting them with catalytically inactive spectator ligands to construct MOFs with larger channels and pores,^34^ only moderate success has been achieved to date. MOFs constructed from elongated ligands tend to suffer from interpenetration as well as framework distortion, whereas MOFs built from mixed functional and spectator ligands have diminished atom efficiency.

**Fig. 1:**
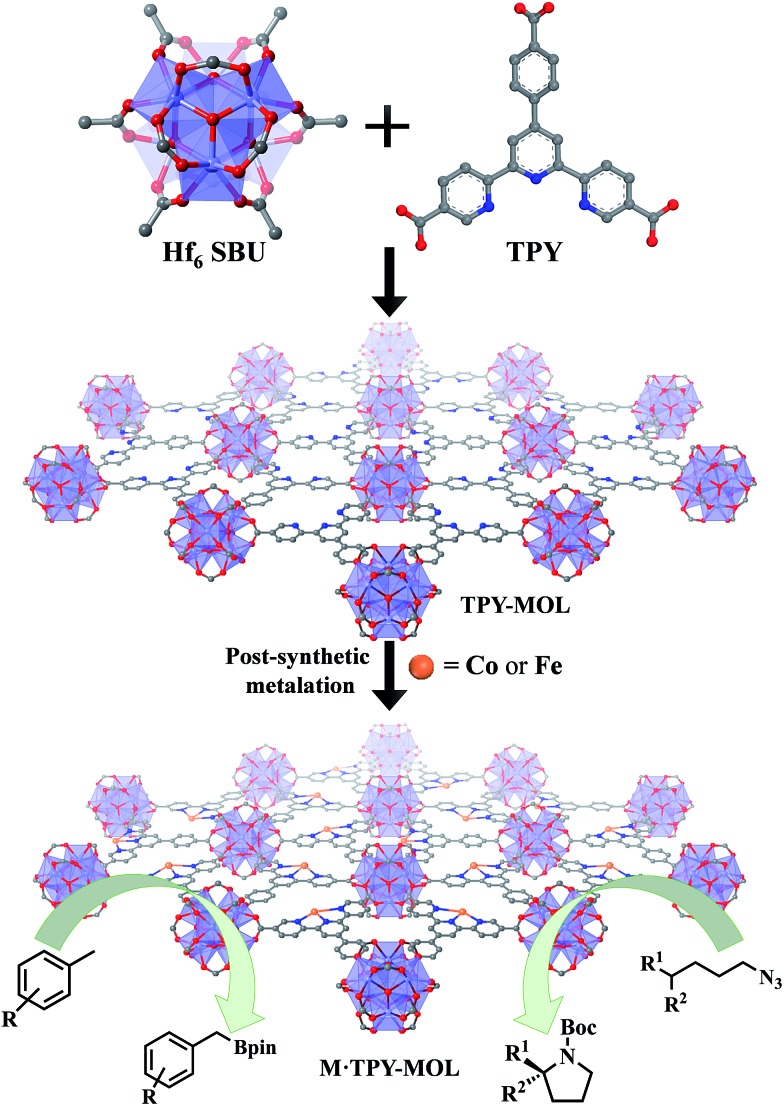
M·TPY-MOLs, constructed from Hf_6_ SBUs and TPY and then metalated with Co and Fe, were used for benzylic C–H borylation and intramolecular sp^3^ C–H Amination reactions, respectively.

We recently showed that diffusional constraint of MOFs could be lifted by reducing one dimension of the MOF crystals to only a few nanometers in thickness to afford a new category of 2D materials, metal–organic layers (MOLs).^35^ Unlike 3D MOFs, the active sites in ultrathin 2D MOLs are readily accessible to substrates during catalytic reactions. On the other hand, MOLs still inherit the heterogeneous nature, ordered structure, and molecular tunability of MOF catalysts,^36^–^38^ and have the potential to provide a rare 2D molecular material platform for designing a new class of single-site solid catalysts without diffusional constraints. We report here the synthesis of a new metal–organic layer, TPY-MOL, based on Hf_6_(μ_3_-O)_4_(μ_3_-OH)_4_(HCO_2_)_6_ secondary building units (SBUs) and 4′-(4-carboxyphenyl)-[2,2′:6′,2′′-terpyridine]-5,5′′-dicarboxylate (TPY) bridging ligands and the metalation of TPY ligands in TPY-MOL with CoCl_2_ and FeBr_2_ to afford highly effective recyclable and reusable MOL catalysts for challenging benzylic C–H borylation and intramolecular sp^3^ C–H amination reactions (Fig. 1). Spectroscopic and computational studies identified unprecedented Co^II^/Fe^II^-terpyridine diradical complexes as catalytic active sites for the borylation and amination reactions.

Owing to their distinct coordination, redox, and photophysical properties, terpyridines (tpy) and their metal complexes have been explored for potential applications in many fields, including polymer science,^39^,^40^ optoelectronics,^41^,^42^ medicinal chemistry,^43^,^44^ nanotechnology,^45^ and molecular catalysis.^41^,^46^,^47^ Although tpy derivatives provide a potentially interesting ligand platform for designing earth-abundant metal catalysts, few examples have been reported in the literature,^47^–^50^ in part due to their strong propensity to undergo disproportionation reactions to form catalytically inactive M(tpy)_2_ complexes.^48^,^49^ Installation of bulky groups on the 6,6′′-positions of tpy could prevent such bimolecular deactivation processes in M-tpy catalysts but often at the expense of their catalytic activities.^48^ By incorporating a tpy derivative into the TPY-MOL, we effectively shut down the disproportionation decomposition pathway without relying on steric protection at the 6,6′′ positions and obtained highly effective MOL catalysts based on M-tpy complexes (M = Co or Fe) for benzylic C–H borylation and intramolecular sp^3^ C–H amination reactions. The MOL-based M-tpy catalysts displayed at least 20 times higher catalytic activity and distinct chemoselectivity in benzylic C–H borylation reactions and 50 times higher TONs in intramolecular sp^3^ C–H amination reactions over their homogeneous analogs.

## Results and discussion

### Synthesis and postsynthetic metalation of TPY-MOL

**Fig. 2:**
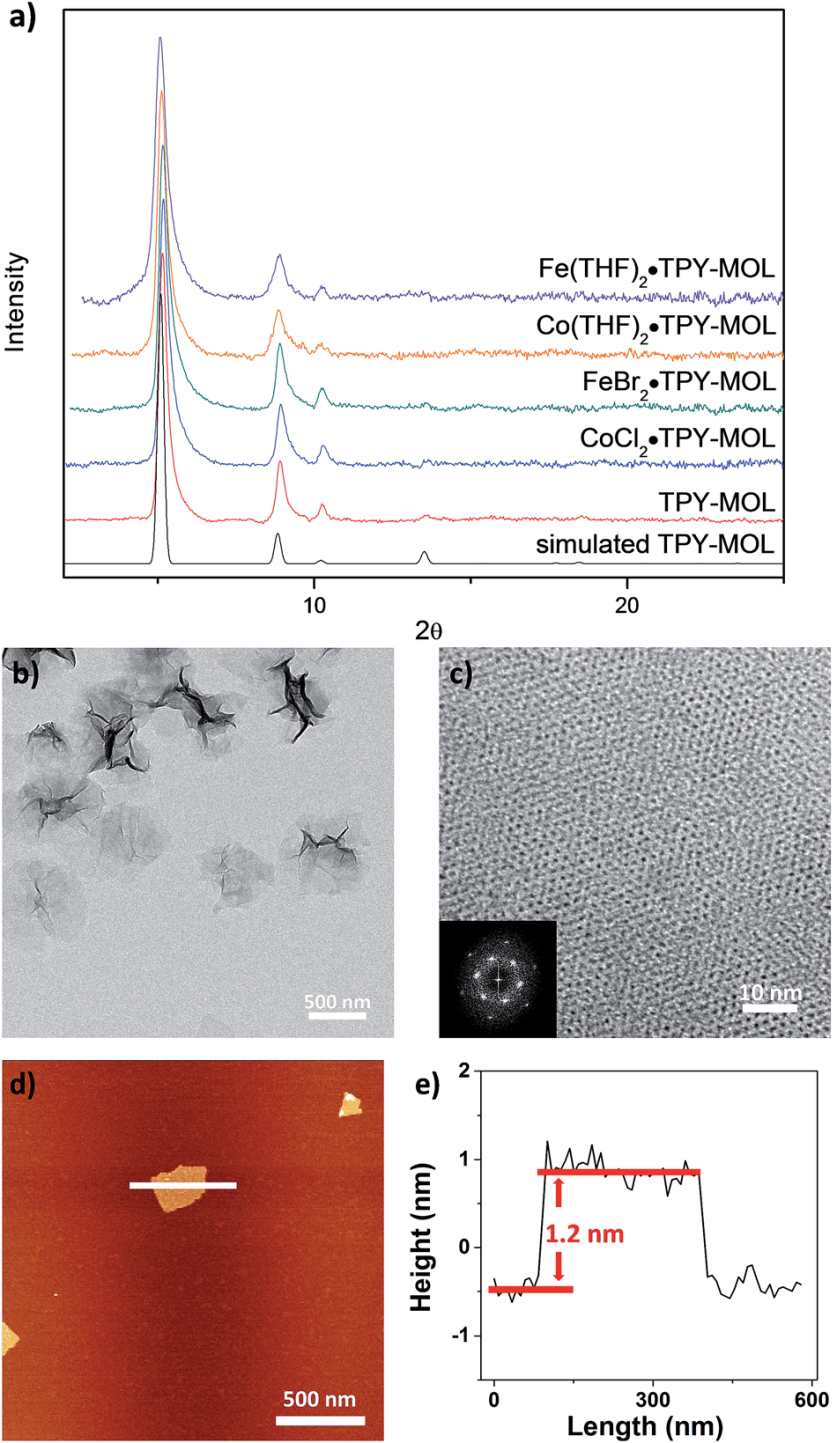
(a) PXRD patterns of TPY-MOL, CoCl_2_·TPY-MOL, FeBr_2_·TPY-MOL, Co(THF)_2_·TPY-MOL, and Fe(THF)_2_·TPY-MOL in comparison to simulated PXRD pattern for TPY-MOL; (b) TEM image of TPY-MOL; (c) HRTEM image and fast Fourier transform (FFT) pattern of TPY-MOL; (d) tapping-mode atomic-force microscope (AFM) topographic image of TPY-MOL; (e) height profile along the white line of TPY-MOL.

TPY-MOL was synthesized in 76% yield by heating a mixture of HfCl_4_, H_3_TPY, and formic acid in DMF and water at 120 °C for 24 h. The PXRD pattern of TPY-MOL matched the simulated pattern based on the (*hk*0) reflections only that are characteristic of 2D MOL structures and aligned well with that of isostructural BTB-MOL (BTB is 1,3,5-benzenetribenzoate, Fig. 2a).^35^ Transmission Electron Microscopy (TEM) images showed ultra-thin films of TPY-MOL whereas the high resolution TEM (HRTEM) images of TPY-MOL showed a clear lattice with the dark spots corresponding to Hf_6_ clusters (Fig. 2b and c). The distances between adjacent spots on the HRTEM image (20.1 Å) matched well with that between two adjacent Hf_6_ SBUs (20.0 Å) in the MOL structural model. Atomic Force Microscopy (AFM) images of TPY-MOL indicated monolayer thickness for many nano-sheets with an average measured thickness of 1.2 nm, corresponding to the van der Waals size of Hf_6_ SBUs (Fig. 2d and e).

**Fig. 3:**
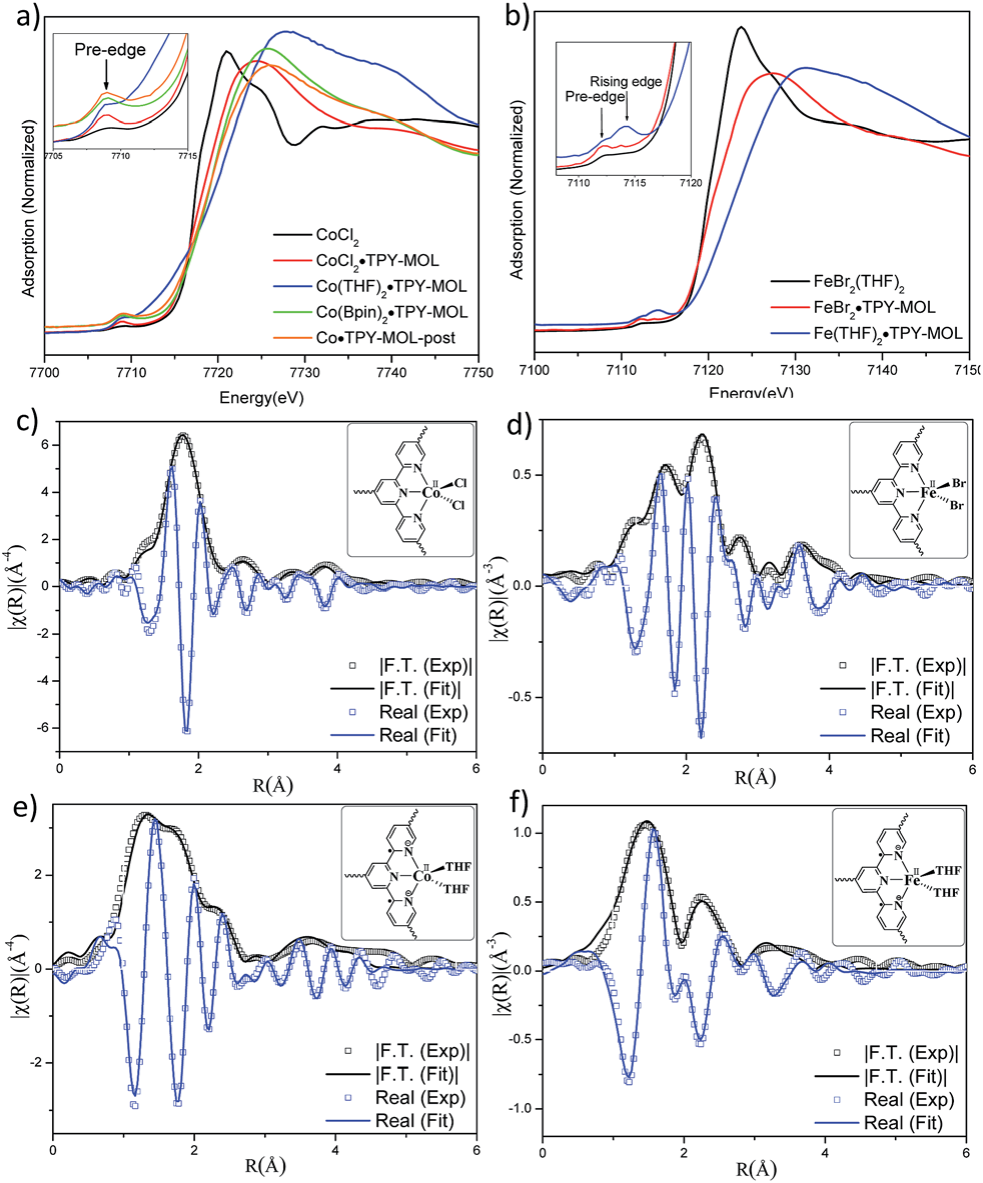
(a) XANES spectra of CoCl_2_, CoCl_2_·TPY-MOL, Co(THF)_2_·TPY-MOL, Co(Bpin)_2_·TPY-MOL, and Co·TPY-MOL-post; (b) XANES spectra of FeBr_2_(THF)_2_, FeBr_2_·TPY-MOL, and Fe(THF)_2_·TPY-MOL; (c–f) experimental EXAFS spectra and fits of CoCl_2_·TPY-MOL, *R* factor = 0.006 (c), FeBr_2_·TPY-MOL, *R* factor = 0.011 (d), Co(THF)_2_·TPY-MOL, *R* factor = 0.013 (e) and Fe(THF)_2_·TPY-MOL, *R* factor = 0.015 (f) in *R* space showing the magnitude of Fourier transform (black hollow squares, black solid line) and real components (blue hollow squares, blue solid line).

TPY-MOL was readily metalated with CoCl_2_ or FeBr_2_(THF)_2_ (1.05 eqv. w.r.t TPY) to afford CoCl_2_·TPY-MOL or FeBr_2_·TPY-MOL with 100% metal loading, as determined by inductively coupled plasma-mass spectrometry (ICP-MS). X-ray absorption near edge structure (XANES) analysis revealed +2 oxidation state for CoCl_2_·TPY-MOL and FeBr_2_·TPY-MOL (Fig. 3a and b). The oxidation state assignments were further confirmed by X-ray photoelectron spectroscopy (XPS, Fig. S15, ESI†). Extended X-ray absorption fine structure (EXAFS) fitting indicated the coordination of Co(ii) to three N atoms of TPY and two chlorides in CoCl_2_·TPY-MOL and the coordination of Fe(ii) to three N atoms of TPY and two bromides in FeBr_2_·TPY-MOL (Fig. 3c and d). The similarity of EXAFS-derived bond distances in CoCl_2_·TPY-MOL (Co–N_c_ = 2.09 ± 0.01 Å, Co–N_t_ = 2.16 ± 0.01 Å and Co–Cl = 2.28 ± 0.01 Å) and crystallographically determined CoCl_2_·tpy distances (Co–N_c_ = 2.071 Å, Co–N_t_ = 2.139 Å and Co–Cl = 2.298 Å) validates the EXAFS fitting results.

### Co-TPY-MOL catalyzed benzylic C–H borylation

We first investigated C–H borylation of *m*-xylene by Co·TPY-MOL. Organoboronic compounds are a useful class of intermediates for forming carbon–carbon and carbon–heteroatom bonds through coupling reactions. C–H borylation with boron reagents such as B_2_pin_2_ is one of the most direct and convenient methods for the synthesis of organoboronic compounds. Although C–H borylation with arenes has been developed in the past two decades, benzylic C–H borylation is still rare (Table S7, ESI†).^27^,^51^–^56^ Upon activation with NaEt_3_BH, CoCl_2_·TPY-MOL (0.5 mol%) catalyzed *m*-xylene borylation with B_2_pin_2_ at 100 °C over 3 days to afford 42% yield of borylated products, with a 4.2 : 1 selectivity favoring the benzylic position (Table 1, entry 1). The borylated products were obtained in 95% yield with a slightly higher selectivity for benzylic borylation (4.6 : 1) when the catalyst loading increased to 1.0 mol% (Table 1, entry 2). The activation of CoCl_2_·TPY-MOL with NaEt_3_BH is necessary for the borylation reaction (Table 1, entry 3). Under identical conditions, a TPY-MOF control, which is isostructural to the previously reported BTB-MOF in which 2D layers stack in a staggered arrangement to result in a 3D MOF,^35^ gave no conversion, likely due to slow diffusion of the substrates and products (Table 1, entry 4). The homogeneous analog gave 2% borylated products with a 5.7 : 1 selectivity favoring the arene C–H bond (Table 1, entry 5). Such moderate arene borylation activity was recently reported for homogenous tpy-Co derivatives.^49^ Active site isolation in MOLs thus not only increases the TON by more than 20 times (over the homogeneous analog) but also afforded unusual selectivity of borylation for the benzylic C–H bond.

**Table 1:** Cobalt-catalyzed C–H borylation of *m*-xylene

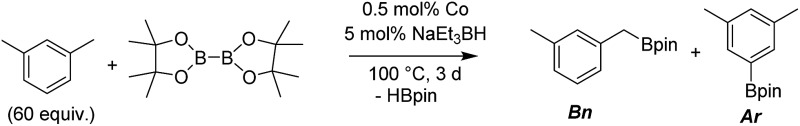		
Entry	Catalyst	Yield^*a*^ (%) (Bn : Ar)
1	CoCl_2_·TPY-MOL	42 (4.2 : 1)
2^*b*^	CoCl_2_·TPY-MOL	95 (4.6 : 1)
3^*c*^	CoCl_2_·TPY-MOL	0
4	CoCl_2_·TPY MOF	0
5	“Homogeneous” CoCl_2_·tpy	2 (1 : 5.7)

^*a*^NMR yield based on CH_3_NO_2_ as an internal standard.

^*b*^1.0 mol% Co.

^*c*^Without the addition of NaEt_3_BH.

We further investigated the substrate scope for Co(THF)_2_·TPY-MOL catalyzed C–H borylation reactions. Benzylic borylated products were produced exclusively for *p*-xylene, 1-*t*-butyl-4-methylbenzene, and mesitylene in >90% yields (Table 2, entries 2–4). For *p*-methoxytoluene, a high selectivity of 59: 6: 1 was obtained for the benzylic borylated product (Table 2, entry 5). For toluene, borylated products were obtained in 92% yield, but the selectivity for the benzylic borylation product was moderate (Table 2, entry 6). These results indicate the influence of steric hindrance on the selectivity of benzylic *vs.* aromatic borylation by Co(THF)_2_·TPY-MOL.

**Table 2:** Co·TPY-MOL catalyzed C–H borylation of arenes^*a*^

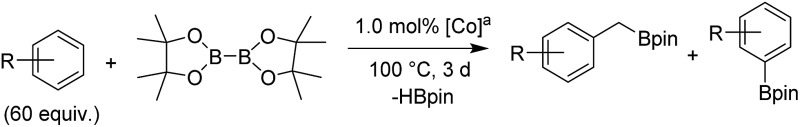			
Entry	Substrate	Product	Yield (%) (Bn : Ar)
1	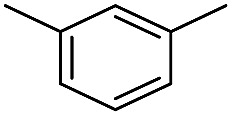	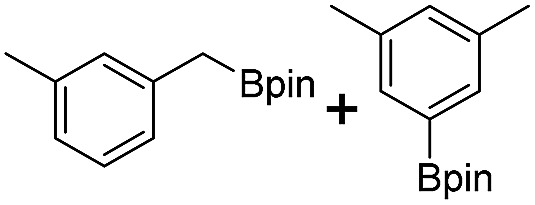	95 (4.6 : 1) (73 : 14)^*c*^
2	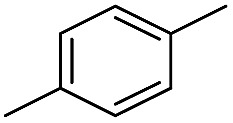	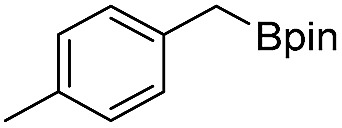	93 (88)^*c*^
3^*b*^	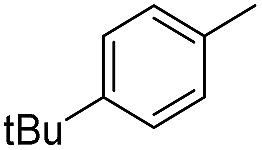	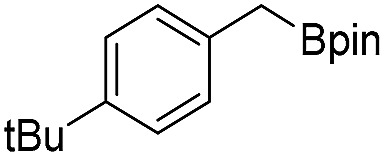	91
4	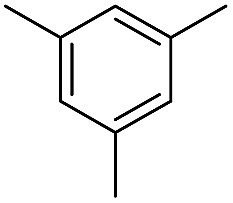	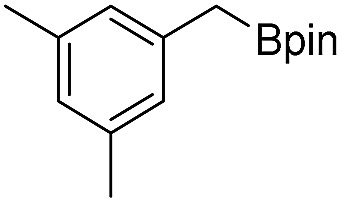	91 (84)^*c*^
5^*b*^	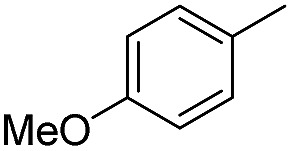	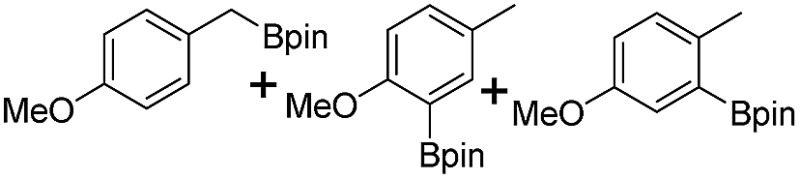	86 (59 : 6 : 1)
6	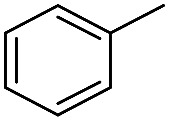	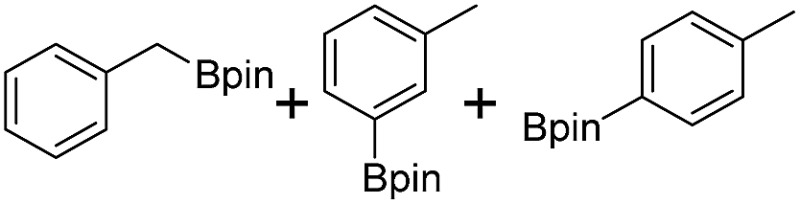	92 (0.91 : 1.4 : 1)

^*a*^[Co] = Co(THF)_2_·TPY-MOL, NMR yield with CH_3_NO_2_ as an internal standard.

^*b*^4 mol% [Co].

^*c*^Isolated yields are shown in parentheses.

Co·TPY-MOL was recovered and used for at least 10 times without any loss of activity in C–H borylation of *p*-xylene (Fig. S32, ESI†). We conducted several tests to demonstrate the heterogeneity of Co·TPY-MOL. First, we showed that the PXRD of Co·TPY-MOL recovered from C–H borylation of *p*-xylene remained the same as that of freshly prepared Co·TPY-MOL (Fig. S33, ESI†). Second, we used ICP-MS to show that the amounts of Co and Hf leaching into the supernatant during the C–H borylation of *p*-xylene were only 0.092% and 0.037% respectively. Finally, we observed that the removal of Co·TPY-MOL from the reaction mixture after several hours stopped the C–H borylation of *p*-xylene (Scheme S2, ESI†).

### Identification of the Co(THF)_2_·TPY-MOL catalyst

**Fig. 4:**
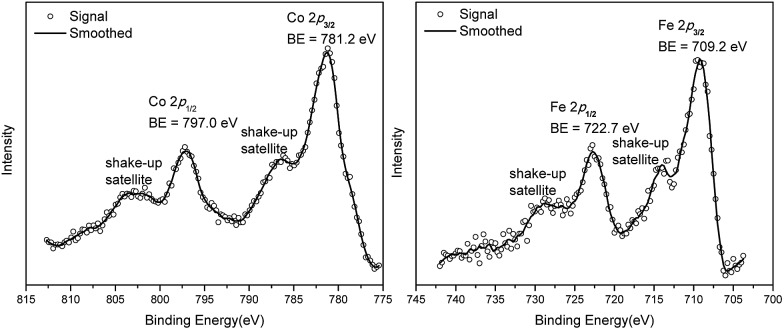
Co 2p and Fe 2p XPS spectra of Co(THF)_2_·TPY-MOL (left) and Fe(THF)_2_·TPY-MOL (right).

We studied the catalytically active species by hydrogen quantification, infrared (IR), UV-Vis-NIR, XPS, and electron paramagnetic resonance (EPR) spectroscopy, XANES, EXAFS, and density functional theory (DFT) calculations. One equiv. of H_2_ was generated upon treatment of CoCl_2_·TPY-MOL with NaEt_3_BH, suggesting the formation of Co(THF)_*x*_·TPY-MOL *via* reductive elimination of H_2_ from the putative CoH_2_·TPY-MOL intermediate. This 2-electron reduction process was also confirmed by titration of Co(THF)_*x*_·TPY-MOL with ferrocenium hexafluorophosphate which resulted in the generation of two equiv. of ferrocene w.r.t to CoTPY-MOL (Fig. S6, ESI†). IR spectra showed no characteristic band of N≡N, ruling out the coordination of dinitrogen to Co. XANES analysis indicated +2 oxidation state for the Co center (Fig. 3a). This oxidation state assignment was further supported by XPS spectroscopy which gave a Co 2p_3/2_ binding energy of 781.2 eV with the expected shake-up peak for the Co^II^ centers (Fig. 4).

The EXAFS spectra at the Co K-edge were well fitted with a structural model in which Co coordinates with three N atoms of TPY and two THF molecules (Fig. 3e). Co–N bond distances (Co–N_c_ = 1.81 ± 0.02 Å, Co–N_t_ = 1.92 ± 0.02 Å) are shorter than those of the reported [Co^I^(tpy)_2_]^57^ (Co–N_c_ = 2.003 Å, Co–N_t_ = 2.130 Å), arguing against the +1 oxidation state for Co(THF)_2_·TPY-MOL. Furthermore, Co(THF)_2_·TPY-MOL has shorter Co–N bond distances than those for Co^II^Cl_2_·TPY-MOL (Co–N_c_ = 1.90 ± 0.01 Å, Co–N_t_ = 2.09 ± 0.01 Å), but similar Co–N bond distances to a reported low-spin Co^II^(tpy)(BH_4_) complex with the (tpy˙)^–^ ligand (Co–N_c_ = 1.810 Å, Co–N_t_ = 1.925 Å).^58^ The Co–N bond distance analysis thus supports the formulation of the Co^II^-(tpy˙˙)^2–^ electronic structure for Co(THF)_2_·TPY-MOL.

**Fig. 5:**
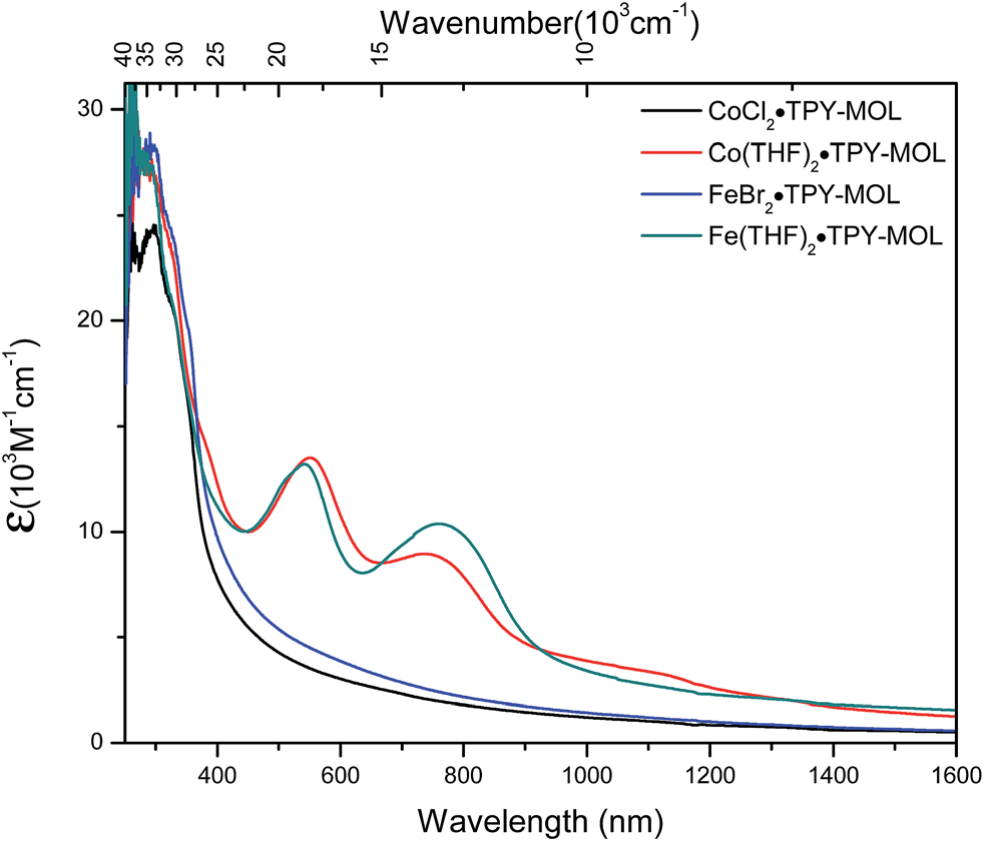
UV-Vis-NIR absorption spectra of CoCl_2_·TPY-MOL, Co(THF)_2_·TPY-MOL, FeBr_2_·TPY-MOL, and Fe(THF)_2_·TPY-MOL in THF at 25 °C.

We used UV-Vis-NIR spectroscopy to discern the diradical nature of TPY ligands in CoTPY-MOLs (Fig. 5). Co(THF)_2_·TPY-MOL exhibited two intense, broad bands centered at 552 and 759 nm and a weak but broad band at 1105 nm, indicative of π to π* and π* to π* transitions for the reduced tpy ligand.^59^–^63^ In contrast, these bands are absent in CoCl_2_·TPY-MOL with the neutral TPY ligand (Fig. 5). The proposed (tpy˙˙)^2–^ species was previously observed in reduced M(tpy)_2_ complexes, such as Cr^III^(tpy)_2_, V^IV^(tpy)_2_, and Ti^IV^(tpy)_2_, by Wieghardt and coworkers.^62^,^63^ However, we are not aware of any example of M-tpy complexes featuring the (tpy˙˙)^2–^ species.

**Fig. 6:**
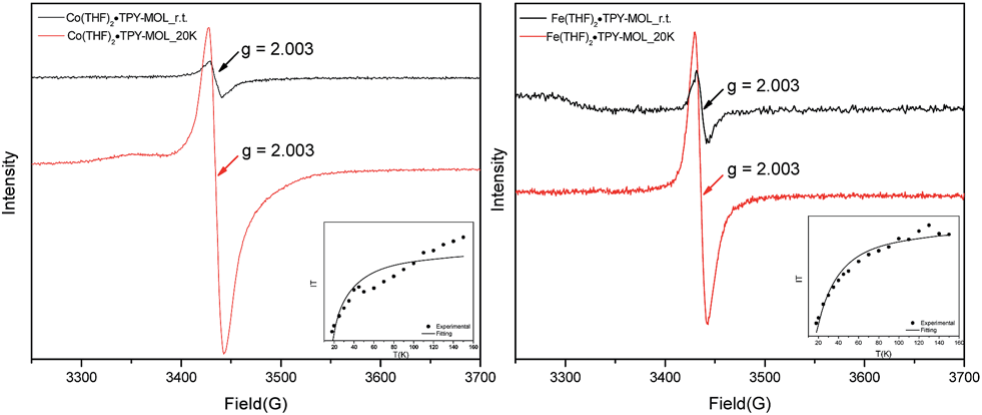
X-band EPR spectra of Co(THF)_2_·TPY-MOL (left) and Fe(THF)_2_·TPY-MOL (right) suspended in toluene at r.t. and 20 K. Microwave frequency: 9.629 GHz for Co(THF)_2_·TPY-MOL at r.t.; 9.629 GHz for Co(THF)_2_·TPY-MOL at 20 K; 9.634 GHz for Fe(THF)_2_·TPY-MOL at r.t.; 9.630 GHz for Fe(THF)_2_·TPY-MOL at 20 K. Insets are temperature-dependent EPR intensity plots and their fits to the Bleaney and Bowers equation. The fitting results gave a singlet to triplet (TPY˙˙)^2–^ energy gap of 0.04 and 0.10 kcal mol^–1^ for Co(THF)_2_·TPY-MOL (left) and Fe(THF)_2_·TPY-MOL, respectively.

Our XANES, EXAFS, and XPS results clearly indicate the Co^II^ oxidation state for Co(THF)_2_·TPY-MOL whose electronic structure is best described as Co^II^(THF)_2_·(TPY˙˙)^2–^-MOL. The (tpy˙˙)^2–^ diradical dianion can have either a singlet (*S* = 0) or a triplet (*S* = 1) ground state, which can potentially be experimentally differentiated by EPR spectroscopy. EPR spectroscopy of Co(THF)_2_·TPY-MOL gave an isotropic signal with *g*_iso_ = 2.003 at r.t. in toluene suspension. The same MOL sample frozen at 20 K exhibits a stronger isotropic signal with *g*_iso_ = 2.003, confirming that the same species was detected at r.t. and 20 K (Fig. 6). More interestingly, the *g* value falls in the range of 2.003–2.005,^59^,^64^,^65^ where radicals in extended organic π systems were often observed. The EPR signal intensity was temperature-dependent, which can be fitted with the Bleaney and Bowers equation^66^ typically used for organic diradicals (Fig. 6). The fitting of temperature-dependent EPR signals indicates that the (TPY˙˙)^2–^ diradical has a singlet ground state with singlet-to-triplet energy gap of 0.04 kcal mol^–1^. The observed EPR signal is thus attributed to the thermally populated TPY triplet excited state.^67^ Moreover, a weak signal *g*_iso_ ≈ 2.04 was observed at 20 K, consistent with low-spin Co^II^ centers. Therefore, our EPR data provide strong support to our proposed electronic structure Co^II^(THF)_2_·(TPY˙˙)^2–^-MOL. We have ruled out the possibility of SBU-based free radicals because TPY-MOL treated with NaEt_3_BH exhibited no signal at r.t. or 20 K (Fig. S16, ESI†).

**Fig. 7:**
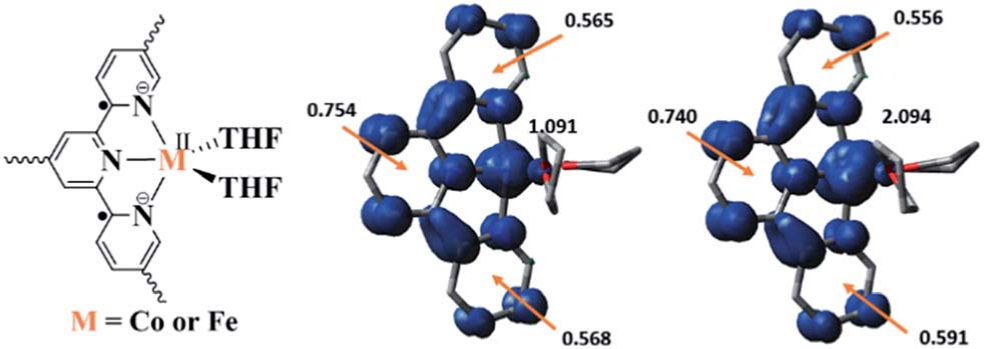
Proposed electronic structure of M^II^(THF)_2_·(TPY˙˙)^2–^-MOL, M = Co or Fe (left); calculated Mulliken spin density distribution and spin density plots (blue: positive; green: negative) of Co(tpy)(THF)_2_ quartet state (middle) and Fe(tpy)(THF)_2_ quintet state (right).

Density functional theory (DFT) calculations and natural population analyses with the B3LYP/6-311G(d) basis set on Co(THF)_2_·tpy gave a doublet ground state (GS) with high positive charge distribution (1.24) on the Co center and negative charge distribution (–1.34) on tpy (Table S9, ESI†). A comparison charge distribution on CoCl_2_·tpy revealed that the Co center in Co(THF)_2_·tpy maintains +2 oxidation state. A Mulliken spin population analysis and spin density plot revealed that 0.996 unpaired electron resides on the Co center, affording a ground state with a low-spin Co^II^, d^7^ doublet (*S*_Co_ = 1/2) and a tpy diradical dianion singlet (*S*_tpy_ = 0) (Fig. S47, ESI†). The singlet tpy diradical dianion is not expected to give any EPR signal. Interestingly, the energy of quartet state of Co(THF)_2_·tpy is calculated to be only 0.40 kcal mol^–1^ higher than that of the doublet GS. This small energy gap is consistent to that deduced from temperature-dependent EPR signals of Co(THF)_2_·tpy. The charge distribution of the quartet state is similar to that of the doublet GS with positive charge (1.29) on the Co center and negative charge (–1.40) on tpy (Table S9, ESI†). The calculated bond distances are similar between the quartet state and the doublet GS (Table S11, ESI†). A Mulliken spin density population and spin density plot of the quartet state revealed the residence of the 1.091 unpaired spin on Co center and 1.887 unpaired spins on tpy, affording a low-spin Co^II^, d^7^ doublet (*S*_Co_ = 1/2) and a tpy triplet diradical dianion (*S*_tpy_ = 1) (Fig. 7). The energetically accessible low-lying triplet excited state of (tpy˙˙)^2–^ was previously proposed for the hypothetical [Zn^II^(tpy^2–^)(NH_3_)_2_]^0^.^62^ DFT calculations thus support the origin of the experimental tpy diradical dianion EPR signal as thermally populated quartet state of Co^II^(THF)_2_·tpy˙˙. Moreover, we believe that conjugation of Hf_6_ SBU to TPY can further stabilize TPY diradical dianion and lower the energy difference between doublet and quartet states of Co^II^(THF)_2_·TPY˙˙-MOL.

We also investigated the activation of CoCl_2_·tpy molecular complex with NaEt_3_BH. Upon treating CoCl_2_·tpy in THF with 10 equiv. of NaEt_3_BH, the mixture turned dark green immediately with concomitant formation of Co nanoparticles as black precipitate (Fig. S7 and S9, ESI†). The solution was filtered through Celite and evaporated to afford Co(tpy)_2_ (HR-MS calculated for C_30_H_22_N_6_Co [M^+^]: 525.1238, found: 525.1257).

### Mechanistic studies of Co(THF)_2_·TPY-MOL catalyzed C–H borylation

To gain insight into the mechanism of the C–H borylation reaction, we carried out several experiments. First, we performed kinetic isotope effect (KIE) studies in order to afford information on the rate-determining step of the C–H borylation reactions. The initial rates of C–H borylations with *p*-xylene and *p*-xylene-d_8_ were determined by running parallel reactions in separate vessels, and the comparison of the initial rates gave a KIE value of 2.7 (Scheme S3, ESI†). Such a primary KIE indicates the involvement of the C–H bond breaking in the rate-determining step.

Second, we detected the presence of HBpin by gas chromatography-mass spectrometry (GC-MS) at the end of the C–H borylation reactions. Third, we determined the resting state of the catalyst by EXAFS studies. By treating Co(THF)_2_·TPY-MOL with 20 equiv. of B_2_pin_2_, we obtained the Co(Bpin)_2_·TPY-MOL product in which Co coordinates to three N atoms of TPY and two Bpin groups according to EXAFS fitting (Fig. S13, ESI†). To determine the resting state of the catalyst, the C–H borylation reaction was stopped at 70% conversion and the organic volatiles were evaporated. EXAFS studies indicated that the remaining residue had the same structure as Co(Bpin)_2_·TPY-MOL (Fig. S14, ESI†). Finally, EPR spectra of Co(Bpin)_2_·TPY-MOL did not show any signals corresponding to a TPY-based radical EPR signal (Fig. S16, ESI†), suggesting a typical Co^II^·TPY complex with negative charge localized on the Bpin ligands.

**Scheme 1:**
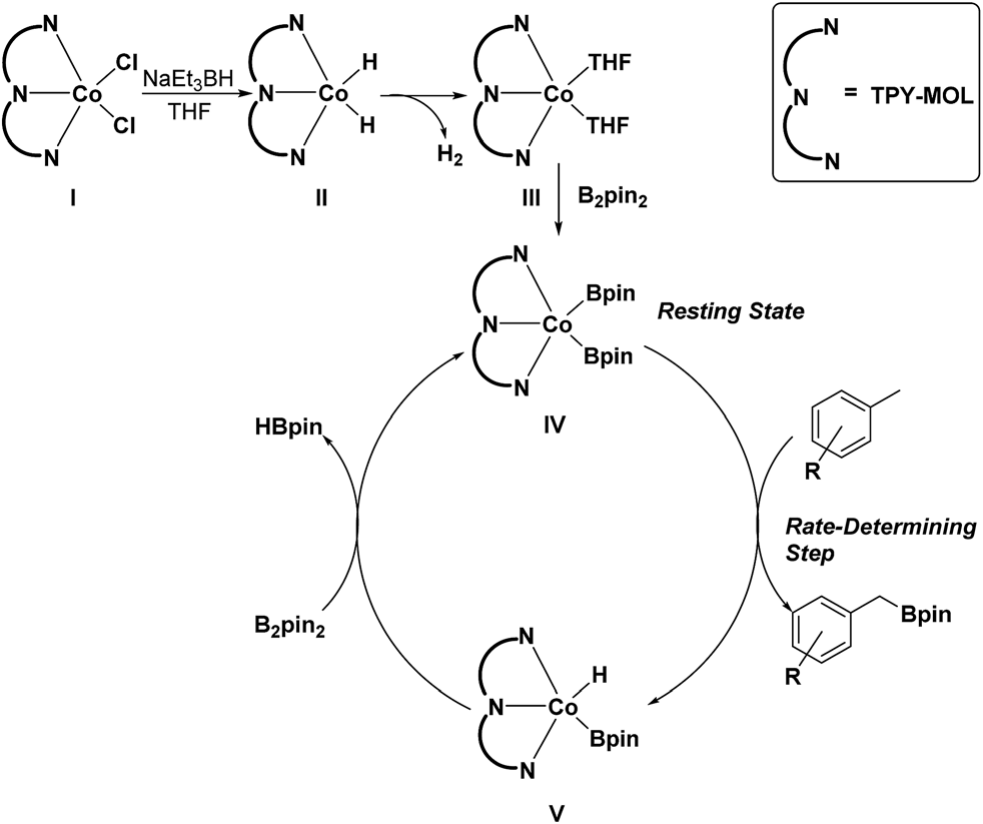
Proposed mechanism for the Co(THF)_2_·TPY-MOL catalyzed C–H borylation of arenes with B_2_pin_2_.

On the basis of these experimental and calculation results, we propose a catalytic cycle for the C–H active borylation of methylarenes as shown in Scheme 1. The CoCl_2_·TPY-MOL(i) is activated by NaEt_3_BH in THF to give the CoH_2_·TPY-MOL(ii) intermediate, which quickly undergoes reductive elimination of H_2_ to produce the Co^II^(THF)_2_·(TPY˙˙)^2–^-MOL(iii) catalyst. Oxidative addition of B_2_(pin)_2_ to III results in Co(Bpin)_2_·TPY-MOL(iv), which is the catalyst resting state for the C–H borylation reactions. σ-Bond metathesis between IV and methylarene proceeds as a rate-determining step to form Co(H)(Bpin)·TPY(v) and the benzylic borylated product. The reaction of V with B_2_pin_2_ regenerates the intermediate IV and forms HBpin as a byproduct *via* σ-Bond metathesis. The transformation of V to IV could alternatively involve a two-step process of reductive elimination of HBpin from V followed by oxidative addition of B_2_Pin_2_ to the intermediate to form IV. We are not able to differentiate between the concerted one-step σ-bond metathesis and the two-step reductive elimination/oxidative addition process.

### Fe·TPY-MOL catalyzed intramolecular sp^3^ C–H amination

TPY-MOL was also metalated with FeBr_2_(THF)_2_ to generate FeBr_2_·TPY-MOL. Similar to the Co(THF)_2_·TPY-MOL case, when FeBr_2_·TPY-MOL was treated with 10 equiv. of NaEt_3_BH, Fe(THF)_2_·TPY-MOL was generated along with 1 equiv. of H_2._ This 2-electron reduction process was also confirmed by titration of Fe(THF)_2_·TPY-MOL with ferrcenium hexafluorophosphate which resulted in the generation of two equiv. of ferrocene. EXAFS fitting indicates Fe coordinates to three N from TPY and two THF molecules for Fe(THF)_2_·TPY-MOL (Fig. 3f) while infrared spectroscopy indicates no coordination of dinitrogen to Fe centers. The oxidation state of Fe(THF)_2_·TPY MOL was determined to be +2 by XANES analysis since the pre-edge position for Fe(THF)_2_·TPY-MOL (7111.6 eV) aligned well with FeBr_2_(THF)_2_ (7111.5 eV), FeBr_2_·TPY-MOL (7111.5 eV) and two reported five-coordinate species (^iPr^PDI)FeCl_2_ (7111.8 eV) and (^iPr^PDI)Fe(N_2_)_2_ (7111.9 eV).^68^ Interestingly, a second feature at 7113.2 eV was observed for Fe(THF)_2_·TPY-MOL, assignable to the 1s to ligand π* transitions. This feature was also seen in a reported (^iPr^PDI^2–^)Fe^II^(N_2_)_2_ species (7114.0 eV). It is worth mentioning that [Fe(tpy)_2_]^*n*+^ (*n* = 0, 1, 2) were all determined to have Fe^II^ centers.^69^ Furthermore, XPS spectroscopy clearly shows Fe^II^ oxidation state for Fe(THF)_2_·TPY-MOL based on characteristic Fe 2P_3/2_ binding energy of 709.2 eV and shake-up peaks (Fig. 4). The electronic spectrum of Fe(THF)_2_·TPY-MOL is very similar to that of Co^II^(THF)_2_·(TPY˙˙)^2–^-MOL, indicating the presence of (TPY˙˙)^2–^ diradical dianion on Fe(THF)_2_·TPY-MOL (Fig. 5). Fe(THF)_2_·TPY-MOL gave an EPR signal with *g*_iso_ = 2.003 at r. t. in a toluene suspension. The same MOL sample frozen at 20 K exhibited a stronger signal with *g*_iso_ = 2.003 (Fig. 4). The fitting of temperature-dependent EPR signals indicates that the (TPY˙˙)^2–^ diradical has a singlet ground state with singlet-to-triplet energy gap of 0.10 kcal mol^–1^. The observed EPR signal is thus attributed to the thermally populated TPY triplet excited state (Fig. 6).^67^ Therefore, the EPR data provide strong evidence of our proposed electronic structure of the Fe^II^(THF)_2_·(TPY˙˙)^2–^-MOL catalyst.

DFT calculations and natural population analyses with the B3LYP/6-311G(d) basis set on Fe(THF)_2_·tpy gave a triplet GS with high positive charge distribution (1.29) on the Fe center and negative charge distribution (–1.39) on tpy (Table S10, ESI†). Spin density plot of the GS revealed that 2.013 unpaired electrons reside on the Fe center, affording an intermediate-spin Fe^II^, d^6^ center (*S*_Fe_ = 1), and a tpy singlet diradical dianion antiferromagnetically coupled to each other (*S*_tpy_ = 0) (Fig. S51, ESI†). The GS of Fe(THF)_2_·tpy again is not expected to give any organic radical EPR signal, which contradicts our experimental results. We believe that the experimental tpy EPR signal comes from thermal population of the quintet state of Fe(THF)_2_·tpy which is only 5.26 kcal mol^–1^ higher in energy than that of triplet GS, consistent to our EPR analysis. The charge distribution of the quintet state is similar to that of triplet GS with positive charge (1.34) on the Fe center and negative charge (–1.44) on tpy (Table S10, ESI†). A Mulliken spin population analysis and spin density plot revealed that 2.094 unpaired spins reside on the Fe center and 1.887 unpaired spins on tpy, affording an intermediate-spin Fe^II^, d^6^ compound (*S*_Fe_ = 1), and a tpy triplet diradical dianion (*S*_TPY_ = 1) (Fig. 7), which is consistent with our experimental EPR results. The coordination of Hf_6_ SBUs to TPY is expected to further stabilize TPY diradical dianion and lower the energy difference between triplet and quintet states of Fe^II^(THF)_2_·(TPY˙˙)^2–^-MOL.

Upon activation with NaEt_3_BH, 2 mol% of FeBr_2_·TPY-MOL catalyzed intramolecular C_sp^3^_–H amination of 1-azido-4-phenylbutane (**1a**) in the presence of two equivalents of di-*tert*-butyl dicarbonate (Boc_2_O) at 90 °C to form Boc-protected α-phenyl pyrrolidine (**2a**) in 89% yield. This level of activity is 9 times as high as that of the MOF control (Table 3, entry 4). Under identical conditions, the homogeneous tpy-Fe catalyst only afforded the product in 3% yield, probably due to the deactivation of tpy-Fe catalyst *via* bimolecular pathways (Table 3, entry 5). Indeed, treatment of FeBr_2_·tpy with 10 equiv. of NaEt_3_BH produced a mixture Fe(tpy)_2_ and Fe nanoparticles; such a disproportionation reaction was previously observed for a series of (PDI)FeBr_2_ complexes.^69^,^70^

**Table 3:** Iron catalyzed intramolecular C–H amination

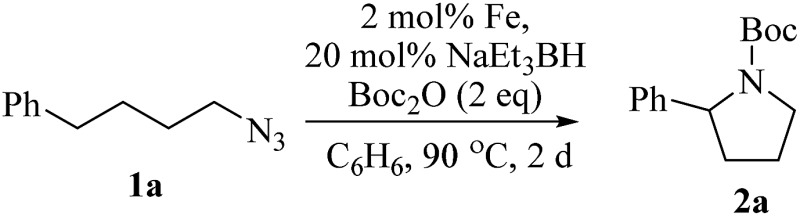			
Entry	Catalyst	Yield^*a*^ (%)	TON
1	FeBr_2_·TPY MOL	89	44.5
2^*b*^	FeBr_2_·TPY MOL	76	76
3^*c*^	FeBr_2_·TPY MOL	16	8
4	FeBr_2_·TPY MOF	10	5
5	“Homogeneous” Fe(tpy)Br_2_	3	1.5

^*a*^NMR yield with MeNO_2_ as an internal standard.

^*b*^1 mol% Fe loading.

^*c*^Without addition of NaEt_3_BH.

A higher TON of 76 was achieved when the Fe loading was decreased to 1 mol% (Table 3, entry 2). With a much simpler ligand, Fe·TPY-MOL outperformed Betley's Fe-dipyrrinato homogenous catalyst by 13 times^71^ and our recently reported NacNac-MOF catalysts by 4 times^28^ in TONs. It is worth noting that FeBr_2_·TPY-MOL, without activation with NaEt_3_BH, showed low activity (Table 3, entry 3), suggesting that the formation of Fe-nitrene compound might be a key elementary step of the intramolecular C_sp^3^_–H amination reaction.^71^–^77^

**Fig. 8:**
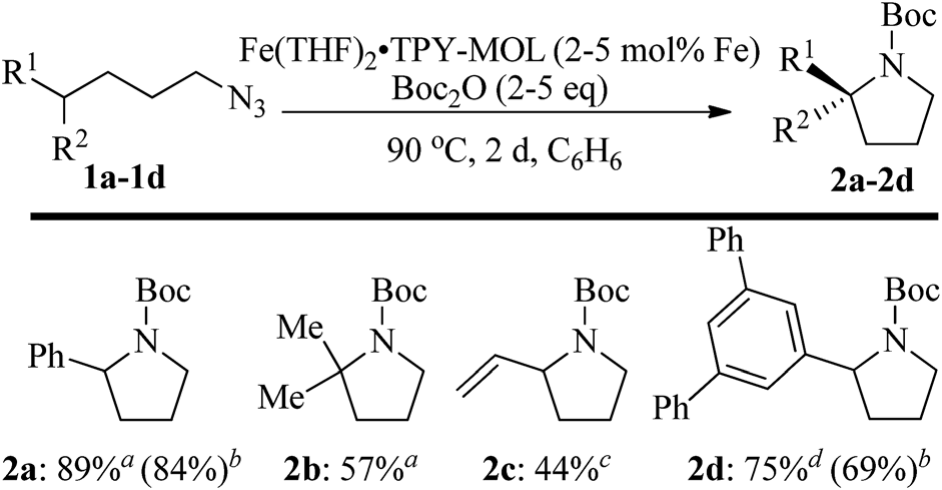
Substrate scope for α-substituted pyrrolidine synthesis. Reaction conditions: ^*a*^Fe (2 mol%), Boc_2_O (2 equiv.); ^*b*^isolated yields. ^*c*^Fe (2 mol%), Boc_2_O (5 equiv.); ^*d*^Fe (5 mol%), Boc_2_O (2 equiv.).

We further explored the substrate scope of intramolecular C_sp^3^_–H amination reactions (Fig. 8). At 2 mol% catalyst loading and in the presence of 2 equiv. of Boc_2_O, the 2,2-dimethylpyrrolidine (**2b**) was formed in 57% yield. Due to reactivity of the vinyl substituent in **2c**, 5 eq. of Boc_2_O was required to give modest yield at 2 mol% Fe. Since the MOL catalysts are free from diffusion constraints, substrates with a bulky substituent such as 3,5-diphenylphenyl was also tolerated and gave 75% yield at 5 mol% Fe and 2 eq. of Boc_2_O.

**Fig. 9:**
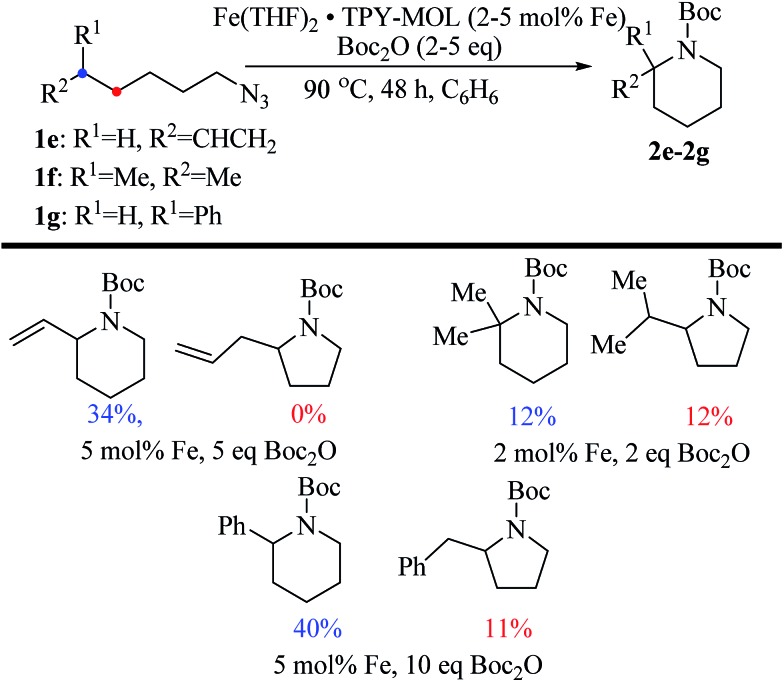
Substrate scope for α-substituted piperidine synthesis.

Piperidines can also be formed *via* C–H amination with the Fe·TPY-MOL catalyst (Fig. 9). For example, 7-azidohept-1-ene was converted to the exclusively six-member ring product 1-Boc-2-vinylpiperidine in 34% yield. By comparison, Betley's Fe-dipyrrinato homogenous catalyst required a stoichiometric equivalent of catalyst to obtain 45% yield. Furthermore, the 1-Boc-2,2-dimethylpiperidine and 1-Boc-2-phenylpiperidine could also be formed from alkyl azides. In these examples, the pyrrolidine products were also observed.

PXRD pattern of Fe·TPY-MOL catalysts recovered from C_sp^3^_–H amination reactions suggested that the integrity of the MOL was maintained under reaction conditions. ICP-MS of the supernatant showed <0.1% of Fe and <0.1% of Hf had leached into the supernatant. Furthermore, The Fe·TPY-MOL catalyst could be recovered and reused four times (Scheme S4, ESI†).

## Conclusions

We have synthesized a terpyridine-based TPY-MOL and metalated TPY-MOL with CoCl_2_ and FeBr_2_ to generate M·TPY-MOL catalysts for benzylic C–H borylation and C_sp^3^_–H amination reactions. Interestingly, M·TPY-MOL catalysts showed significantly higher activity and different chemo-selectivity than homogeneous and MOF controls. Spectroscopic studies and DFT calculations indicated the formation of unprecedented MOL-stabilized M^II^-(TPY˙˙)^2–^ species featuring divalent metals and TPY diradical dianions. We believe that the formation of novel M^II^-(TPY˙˙)^2–^ (M = Co or Fe) species endows them with unique and enhanced catalytic activities in C_sp^3^_–H borylation and intramolecular amination reactions. Our work demonstrates the ability to engineer MOLs as single-site solid catalysts without diffusional constraints and to elucidate intricate electronic structures of MOL-stabilized metal complexes.

## Conflicts of interest

There are no conflicts to declare.

## Supplementary Material

Supplementary information
