# Supplementary material for: Metal–organic layers stabilize earth-abundant metal–terpyridine diradical complexes for catalytic C–H activation
Source: Chem Sci. 2017 Oct 30;9(1):143–51. doi: 10.1039/c7sc03537c (PMC5869321; doi:10.1039/c7sc03537c)
Supplement: Supplementary file 1 [file SC-009-C7SC03537C-s001.pdf]

Electronic Supplementary Material (ESI) for Chemical Science.
This journal is © The Royal Society of Chemistry 2017

Supporting Information for

Metal-Organic Layers Stabilize Earth-Abundant Metal-Terpyridine Diradical Complexes for Catalytic C-H Activation

Zekai Lin,[†,‡] Nathan C. Thacker,[†,‡] Takahiro Sawano,[†,‡] Tasha Drake,[†] Pengfei Ji,[†] Guangxu Lan,[†] Lingyun Cao,[§] Shubin Liu,[#] Cheng Wang,[§] and Wenbin Lin[†,§,*]

[†]Department of Chemistry, University of Chicago, 929 E. 57th St., Chicago, Illinois 60637, United States

[§]Collaborative Innovation Center of Chemistry for Energy Materials, State Key Laboratory of Physical Chemistry of Solid Surfaces, Department of Chemistry, College of Chemistry and Chemical Engineering, Xiamen University, Xiamen 361005, PR China

[#]Research Computing Center, University of North Carolina, Chapel Hill, North Carolina 27599-3420, United States

Table of Contents

1. General Experimental S2
2. Synthesis and Characterization of TPY-MOLs and TPY-MOFs S2-S8
3. Synthesis and Characterization of Co(tpy)Cl_2, Fe(tpy)Br_2, Co$(tpy)_2$, and Fe$(tpy)_2$ S8-S11
4. X-Ray Absorption Spectroscopy S11-S18
5. X-Ray Photoelectron Spectroscopy S18-S19
6. Electron Paramagnetic Resonance Spectroscopy S19-S21
7. Synthesis of Substrates S21-S24
8. Benzylic C-H Borylation of Arenes with Co•TPY-MOL S24-S36
9. C_{sp}3-H Animation with Fe•TPY-MOL S36-S50
10. Density Functional Theory Calculations S49-S53
11. References S53-54

1. General Experimental

All of the reactions and manipulations were carried out under nitrogen with the use of standard inert atmosphere and Schlenk techniques. ^{1}H NMR spectra were recorded on a Bruker NMR 500 DRX spectrometer at 500 MHz and referenced to the proton resonance resulting from incomplete deuteration of $CDCl_3$ (δ 7.26). ^{13}C NMR spectra were recorded at 125 MHz, and all of the chemical shifts were reported downfield in ppm relative to the carbon resonance of $CDCl_3$ (δ 77.00). The following abbreviations are used here: s: singlet, d: doublet, t: triplet, q: quartet, m: multiplet, br: broad, app: apparent. Mass spectra were obtained with an Agilent 6224 Accurate Mass TOF LC/MS and an Agilent 1100 LC-MSD Mass Spectrometer. Thermogravimetric analysis (TGA) was performed in air using a Shimadzu TGA-50 equipped with a platinum pan. Powder X-ray diffraction (PXRD) patterns were collected on a Bruker D8 Venture, dual microsource (Cu and Mo) diffractometer with a CMOS detector. Cu Kα radiation was used for PXRD. The PXRD patterns were processed with the APEX 2 package using PILOT plug-in. Gas chromatography (GC) data were obtained with an SHIMADZU GC-2010 Plus gas chromatograph. ICP-MS data were obtained with an Agilent 7700x ICP-MS and analyzed using ICP-MS MassHunter version B01.03. Samples were diluted in a 2% HNO_3 matrix and analyzed with a 159Tb internal standard against a nine-point standard curve over the range from 1 ppb to 500 ppb. The correlation coefficient was > 0.9997 for all analyses of interest. Data collection was performed in Spectrum Mode with five replicates per sample and 100 sweeps per replicate. Electron microscopy images were obtained on a Tecnai F20 transmission electron microscopy. Atomic force microscopy (AFM) images were taken on a Bruker V /Multimode 8 instrument. Electron paramagnetic resonance (EPR) spectra were collected with a Bruker Elexsys 500 X-band EPR spectrometer. Tetrahydrofuran (THF) was purified by passing through a neutral alumina column under N_2. $CoCl_2$•tpy and $FeBr_2$•tpy were prepared according to literature procedures.[1, 2]

2. Synthesis and Characterization of TPY-MOL and TPY-MOF

2.1 Synthesis of TPY-MOL

$HfCl_4$ (14.0 mg, 0.044 mmol) and 4'-(4-carboxyphenyl)-[2,2':6',2''-terpyridine]-5,5''-dicarboxylic acid (H_3TPY) [3] (12.5 mg, 0.028 mmol) were added to DMF (5 mL), freshly distilled formic acid (0.75 mL), and water (0.3 mL). The mixture was sonicated for 2-3 minutes and placed

in an oven at 120 °C for 24 hours. The resulting solid was isolated by centrifugation and washed with DMF and THF. Yield: 13.1 mg (76%).

2.2 Synthesis of TPY-MOF

0.2 mL of DEF was added to a mixture of $ZrOCl_2{\cdot}8H_2O$ (3.2 mg, 9.9 µmol), H_3TPY (1.1 mg, 2.5 µmol), and benzoic acid (60 mg, 0.49 mmol). The suspension was sonicated for 2-3 minutes and placed in an oven at 120 °C for 24 hours. The resulting solid was isolated by centrifugation and washed with DMF and THF. Yield: 1.7 mg (58%).

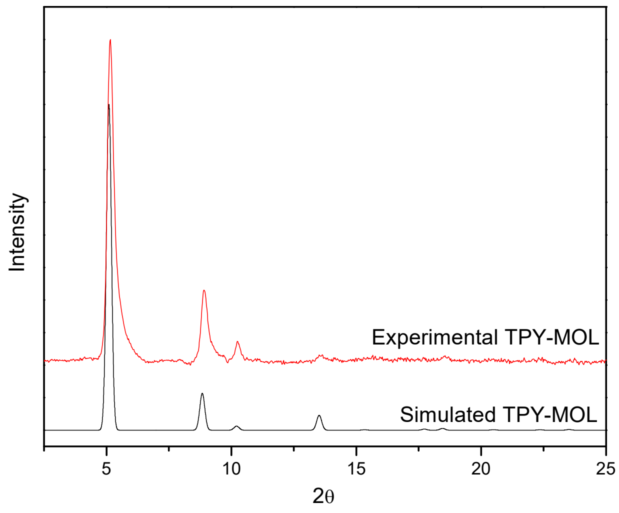

Figure S1. PXRD of TPY-MOL compared to simulated PXRD patterns from TPY-MOL.

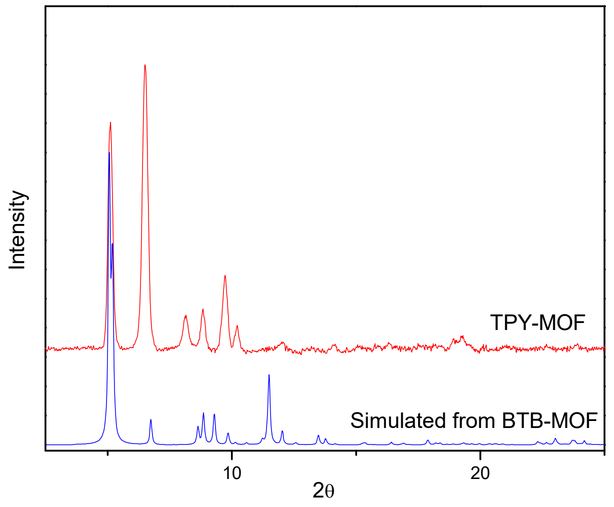

Figure S2. PXRD of TPY-MOF compared to simulated PXRD patterns from BTB-MOF.[3]

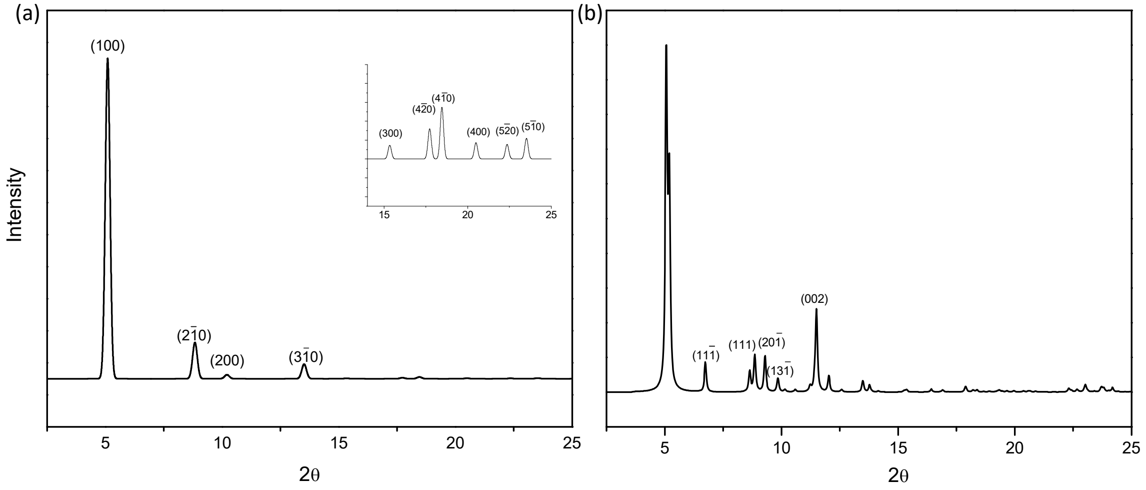

Figure S3. (a) Simulated PXRD of TPY-MOL (inset shows the zoomed-in view of high angle diffractions); (b) Simulated PXRD of BTB-MOF. Only (hk0) reflections are observed for the monolayer MOL structure, which is in stark contrast with the observation of $l \neq 0$ reflections in the BTB-MOF.

2.3 Synthesis of Co•TPY-MOL and Fe•TPY-MOL

$CoCl_2$•TPY-MOL: In a N_2 glovebox, TPY-MOL (4.0 μmol based on TPY) was dispersed in THF (1 mL) and followed by the addition of $CoCl_2$ (4.2 μmol, 1.05 eqv., 0.02 M in THF solution). The resulting mixture was kept at r.t. overnight and then washed with THF for at least three times to obtain $CoCl_2$•TPY-MOL. The extent of Co metalation was determined to be 100% based on TPY equivalents by ICP-MS. Co: 22.23 ± 0.36 ppb; Hf: 194.24 ± 1.28 ppb.

$FeBr_2$•Tpy-MOL: In a N_2 glovebox, TPY-MOL (4.0 μmol based on TPY) was dispersed in THF (1 mL) and followed by the addition of $FeBr_2(THF)_2$ (4.2 μmol, 1.05 eqv., 0.02 M in THF solution). The resulting mixture was kept at r.t. overnight and then washed with THF for at least three times to obtain $FeBr_2$•TPY-MOL. Fe metalation was determined to be 100% based on TPY equivalents by ICP-MS. Fe: 34.91 ± 0.37 ppb; Hf: 327.21 ± 4.84 ppb.

Co(THF)$_2$•TPY-MOL: In a N_2 glovebox, $CoCl_2$•TPY-MOL (4.0 μmol based on Co) was dispersed in THF (1 mL) and followed by the addition of $NaEt_3BH$ (40 μL, 1M THF solution, 10 eqv.). The resulting mixture was kept at r.t. for 1 h and then washed with THF for at least three times to obtain Co(THF)$_2$•TPY-MOL in quantitative yield.

Fe(THF)$_2$•TPY-MOL: In a N_2 glovebox, $FeBr_2$•TPY-MOL (4.0 μmol based on Fe) was dispersed in THF (1 mL) and followed by the addition of $NaEt_3BH$ (40 μL, 1M THF solution, 10 eqv.). The resulting mixture was kept at r.t. for 1 h and then washed with THF for at least three times to obtain Fe(THF)$_2$•TPY-MOL in quantitative yield.

2.4 Quantification of H_2

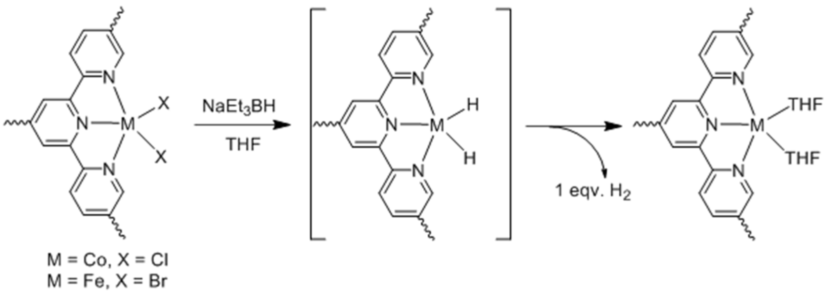

In a N_2 glovebox, $CoCl_2$•TPY-MOL (4 μmol based on Co•TPY) in 1.5 mL benzene was charged into a glass vial. $NaEt_3BH$ (100 μL, 1M toluene solution, 25 eqv.) was added into the vial and the mixture was kept at r.t. for 20 mins. The headspace gas was analyzed by gas chromatography. A parallel experiment with the same amount of TPY-MOL (based on TPY) was also conducted for background removal of H_2 generated from protons at SBUs. Consistent results were obtained in three runs. The amount of H_2 was calculated to be 3.97 ± 0.17 μmole (expected 4 μmole).

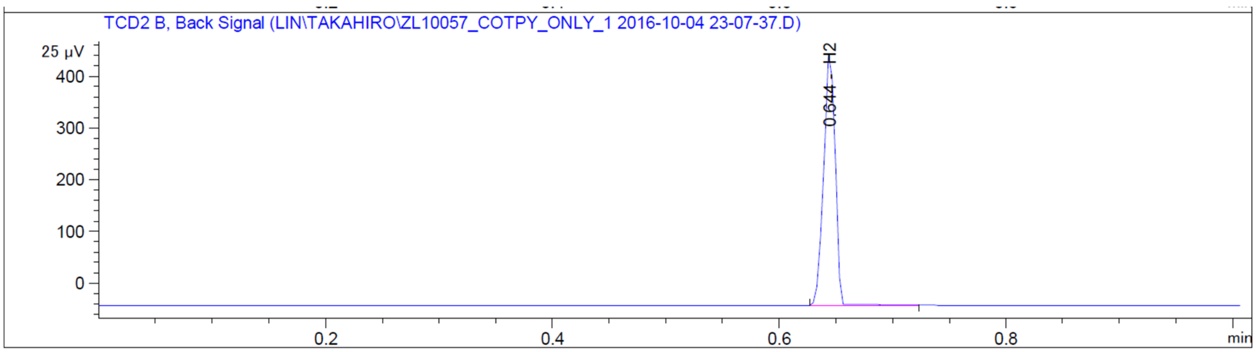

Figure S4. GC trace of H_2 evolved from $CoCl_2$•TPY-MOL when treated with $NaEt_3BH$.

In a N_2 glovebox, $FeBr_2$•TPY-MOL (4 µmol based on Fe•TPY) in 1.5 mL benzene was charged into a glass vial. $NaEt_3BH$ (100 µL, 1M toluene solution, 25 eqv.) was added into the vial and the mixture was kept at r.t. for 20 mins. The headspace gas was analyzed by gas chromatography. A parallel experiment with the same amount of TPY-MOL (based on TPY) was also conducted for background removal of H_2 generated from protons at SBUs. Consistent results were obtained in three runs. The amount of H_2 was calculated to be 4.08 ± 0.31 µmole (expected 4 µmole).

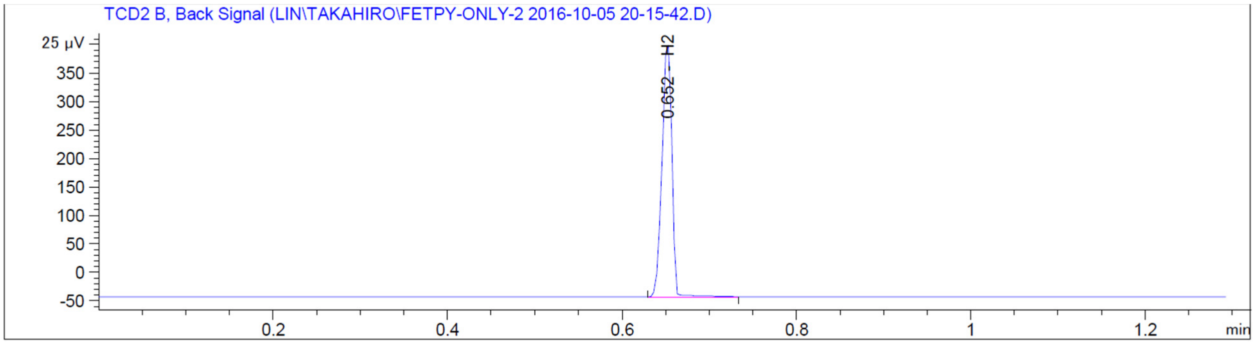

Figure S5. GC trace of H_2 evolved from $FeBr_2$•TPY-MOL reacting with $NaEt_3BH$.

2.5. Quantification of Ferrocenium Reduction

Standard solutions with various amount of ferrocene were added with 20 μmol of mesitylene as internal standard and analyzed by GC-MS to obtain a calibration curve for ferrocene.

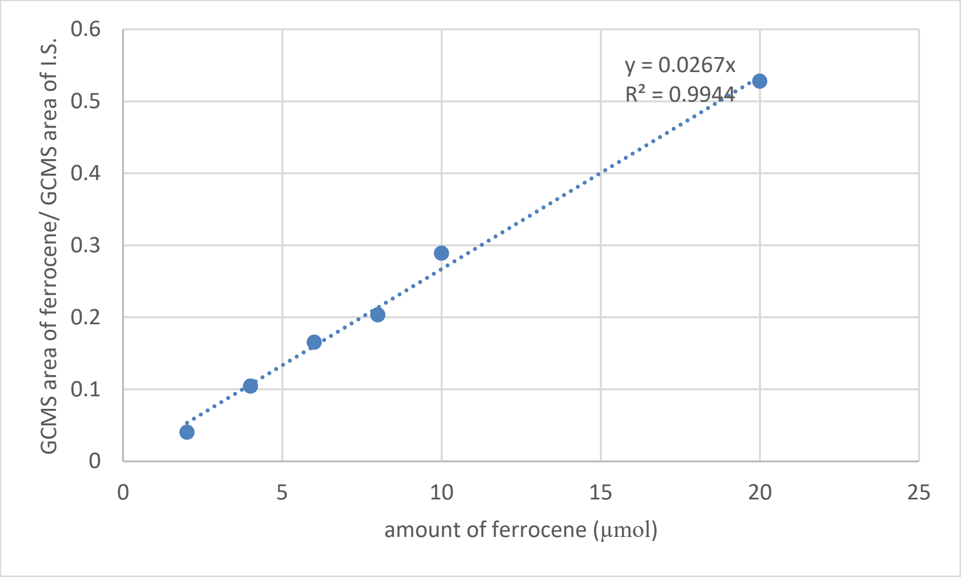

Figure S6. Calibration curve for ferrocene.

In a N_2 glovebox, $Co(THF)_2$•TPY-MOL (5 μmol based on Co•TPY) was titrated with ferrocenium hexaflorophosphate (2 mM in DCM). The blue ferrocenium hexaflorophosphate solution reacted with $Co(THF)_2$•TPY-MOL quickly to turn yellow, indicating the generation of ferrocene. The supernatant was separated from MOL via centrifugation. The titration stopped when the supernatant became green to light blue. All yellow supernatant were combined and added with 20 μmol of mesitylene as internal standard and analyzed by GC-MS to determine the amount of ferrocene generated. 2 equiv. of ferrocene (10.6 ± 2.07 μmol) w.r.t to CoTPY was detected.

In a N_2 glovebox, $Fe(THF)_2$•TPY-MOL (5 μmol based on Co•TPY) was titrated with ferrocenium hexaflorophosphate (2 mM in DCM). The blue ferrocenium hexaflorophosphate solution reacted with $Co(THF)_2$•TPY-MOL quickly to turn yellow, indicating the generation of ferrocene. The supernatant was separated from MOL via centrifugation. The titration stopped when the supernatant became green to light blue. All yellow supernatant were combined and added

with 20 μmol of mesitylene as internal standard and analyzed by GC-MS to determine the amount of ferrocene generated. 2 equiv. of ferrocene (11.6 ± 0.86 μmol) w.r.t to CoTPY was detected.

2.6. Atomic Force Microscopy Studies

MOL samples for AFM imaging were prepared by dropping a few drops of ethanol suspension of TPY-MOL (5 μL) onto a freshly cleaved mica substrate. Measurements were carried out at room temperature in tapping mode with a Si cantilever at a 50 – 90 KHz free vibration frequency and a spring constant of 0.4 N/m. Analyses of the images were done using the off-line software Gwyddion.

3. Synthesis and Characterization of $Co(tpy)Cl_2$, $Co(tpy)_2$, $Fe(tpy)Br_2$, and $Fe(tpy)_2$

3.1 Synthesis of $Co(tpy)Cl_2$ and $Fe(tpy)Br_2$

$CoCl_2$•tpy [CAS: 14854-50-3]: In a N_2–filled glovebox, a solution of terpyridine (200 mg, 0.857 mmol) in THF (20 mL) was slowly added to $CoCl_2$ (0.857 mmol, 0.02 M in THF solution). The resulting mixture was stirred at r.t. for 12 h and then filtered. After washing the resulting solid with THF, the green solid was dried under vacuum to give $Co(tpy)Cl_2$ (248 mg, 80% yield). HR-MS (ESI) calcd for $C_{15}H_{11}Cl_2CoN_3$ $[M]^+$ 361.9662, found 361.9646.

$FeBr_2$•tpy [CAS: 17203-81-5]: In a N_2–filled glovebox, a solution of terpyridine (200 mg, 0.857 mmol) in THF (20 mL) was slowly added to $FeBr_2$ (0.857 mmol, 0.02 M in THF solution). The resulting mixture was stirred at r.t. for 12 h and then filtered. After washing the resulting solid with THF, the purple solid was dried under vacuum to give $Fe(tpy)Br_2$ (258 mg, 67% yield). ESI-MS: calcd for $C_{15}H_{11}BrFeN_6$ $[M-Br]^+$ 368.0, found 368.0.

3.2 Synthesis of $Co(tpy)_2$ and $Fe(tpy)_2$

$Co(tpy)_2$: A mixture of $CoCl_2$•tpy (50 mg, 0.1377 mmol) and $NaBEt_3H$ (1.377 mmol, 1.0 M in THF) in THF (3.0 mL) was stirred at r.t. for 12 h. After removing of Co NPs by centrifugation,

the supernatant was filtered with Celite and dried in vacuo to give Co(tpy)$_2$. HR-MS (ESI) calcd for $C_{30}H_{22}CoN_6$ $[M]^+$ 525.1238, found 525.1257.

Fe(tpy)$_2$ [CAS: 20515-11-1]: A mixture of $FeBr_2$•tpy (50 mg, 0.111 mmol) and $NaBEt_3H$ (1.11 mmol, 1.0 M in THF) in THF (3.0 mL) was stirred at r.t. for 12 h. After removing of FeNPs by centrifugation, the supernatant was filtered with Celite and then dried in vacuo to give Fe(tpy)$_2$. ESI-MS: calcd for $C_{30}H_{22}FeN_6$ $[M]^+$ 522.1, found 522.5.

3.3. Electron Microscopy Images

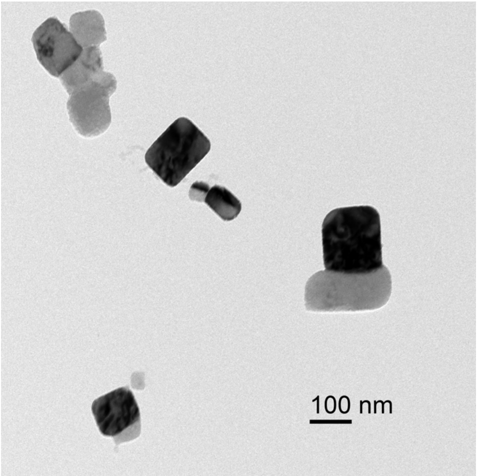

Figure S7. Transmission electron microscope (TEM) image of Co nanoparticles generated by treating $CoCl_2$•tpy with $NaEt_3BH$.

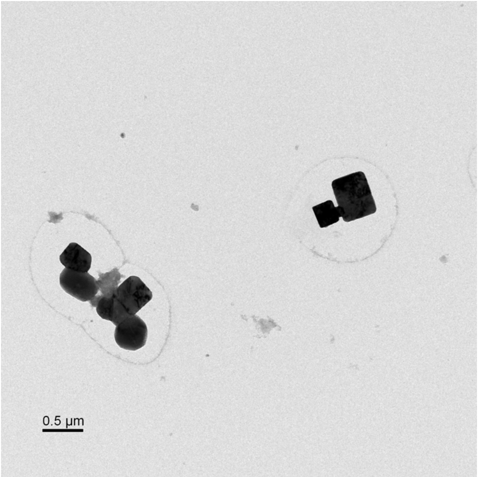

Figure S8. Transmission electron microscope (TEM) image of Fe nanoparticles generated by treating $FeBr_2$•tpy with $NaEt_3BH$.

3.4. Thermogravimetric Analysis

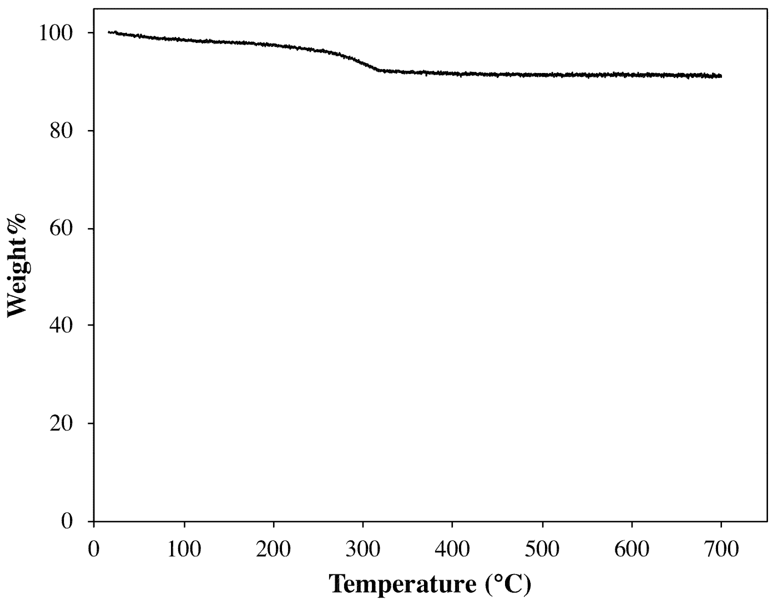

Figure S9. TGA curve of Co nanoparticles generated by treating $CoCl_2$•tpy with $NaEt_3BH$. 3.6% weight loss from 15 to 200° C is from the trapped solvent. 5.4% weight loss from 200 to 700 °C should be from unreacted $CoCl_2$•tpy which has poor solubility in THF. Calculated weight percentage for $CoCl_2$•tpy is 16.2% for Co, 64.2% for tpy and 19.5% for 2Cl. Therefore, the majority of the black precipitates are Co nanoparticles.

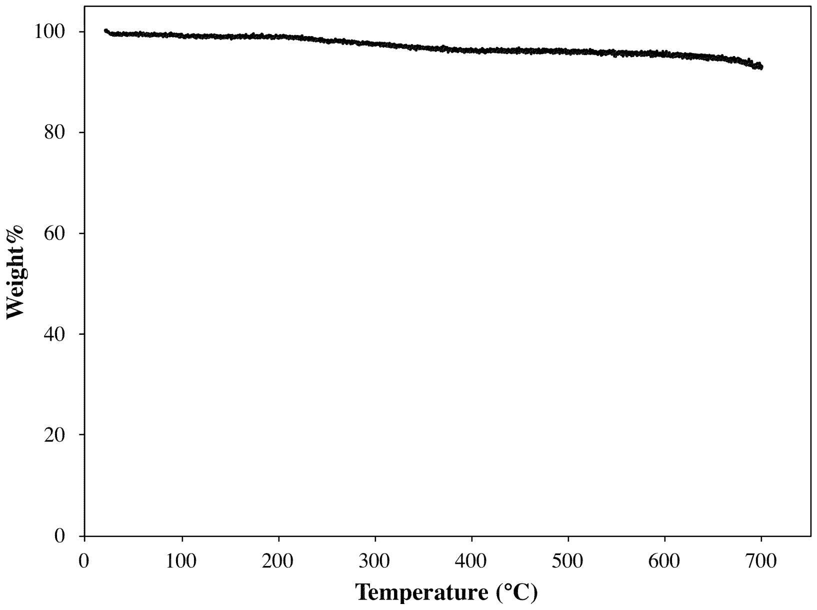

Figure S10. TGA curve of Fe nanoparticles generated by treating $FeBr_2$•tpy with $NaEt_3BH$. 1.0% weight loss from 20 to 200°C is from the trapped solvent. 5.9% weight loss from 200 to 700°C should be from unreacted $FeBr_2$•tpy which has poor solubility in THF. Calculated weight percentage for $FeBr_2$•tpy is 12.4% for Fe, 52.0% for tpy and 35.6% for 2Br. Therefore, the majority of the black precipitates are Fe nanoparticles.

4. X-ray Absorption Spectroscopy

4.1 Data collection.

X-ray absorption data were collected at Beamline 10-BM-A, B at the Advanced Photon Source (APS) at Argonne National Laboratory. Spectra were collected at the cobalt K-edge (7709 eV) or iron K-edge (7112 eV) in transmission mode. The X-ray beam was monochromatized by a Si(111) monochromater and detuned by 50% to reduce the contribution of higher-order harmonics below the level of noise. A metallic cobalt or iron foil standard was used as a reference for energy calibration and was measured simultaneously with experimental samples. For cobalt samples, the incident beam intensity (I_0), transmitted beam intensity (I_t), and reference (I_r) were measured by 20 cm ionization chambers with gas compositions of 63% N_2 and 37% He, 73% N_2 and 27% Ar, and 100% N_2, respectively. For iron samples, the incident beam intensity (I_0), transmitted beam intensity (I_t), and reference (I_r) were measured by 20 cm ionization chambers with gas

compositions of 51% N_2 and 49% He, 79% N_2 and 21% Ar, and 100% N_2, respectively. Data were collected over six regions: -250 to -30 eV (10 eV step size, dwell time of 0.25 s), -30 to -12 eV (5 eV step size, dwell time of 0.5 s), -12 to 30 eV (0.4 eV step size, dwell time of 1 s), 30 eV to 6 $Å^{-1}$, (0.05 $Å^{-1}$ step size, dwell time of 2 s), 6 $Å^{-1}$ to 12 $Å^{-1}$, (0.05 $Å^{-1}$ step size, dwell time of 4 s), 12 $Å^{-1}$ to 15 $Å^{-1}$, (0.05 $Å^{-1}$ step size, dwell time of 8 s). Multiple X-ray absorption spectra were collected at room temperature for each sample. Samples were ground and mixed with polyethylene glycol (PEG) and packed in a 6-shooter sample holder to achieve adequate absorption length.

4.2 Data processing.

Data were processed using the Athena and Artemis programs of the IFEFFIT package based on FEFF 6.[4, 5] Prior to merging, spectra were calibrated against the reference spectra and aligned to the first peak in the smoothed first derivative of the absorption spectrum, the background noise was removed, and the spectra were processed to obtain a normalized unit edge step.

4.3 XANES analysis

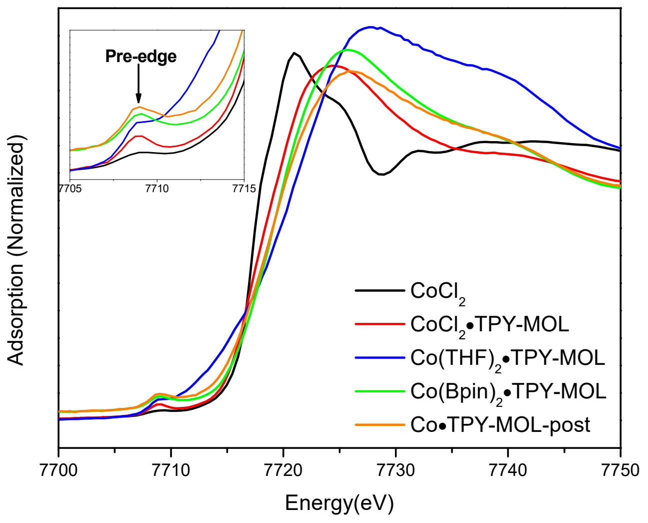

Figure S11. XANES spectra of $CoCl_2$, $CoCl_2$•TPY-MOL, $Co(THF)_2$•TPY-MOL, $Co(Bpin)_2$•TPY-MOL, and Co•TPY-MOL-post.

The oxidation states of Co species within MOLs were determined by the comparison of the energy of the pre-edge positions to the $CoCl_2$ as reference compound. The positions of the pre-

edge align well with that of the reference compound, which is 7708.9 eV. Therefore, we assign the oxidation state of cobalt in all Co-functionalized MOL materials as +2.

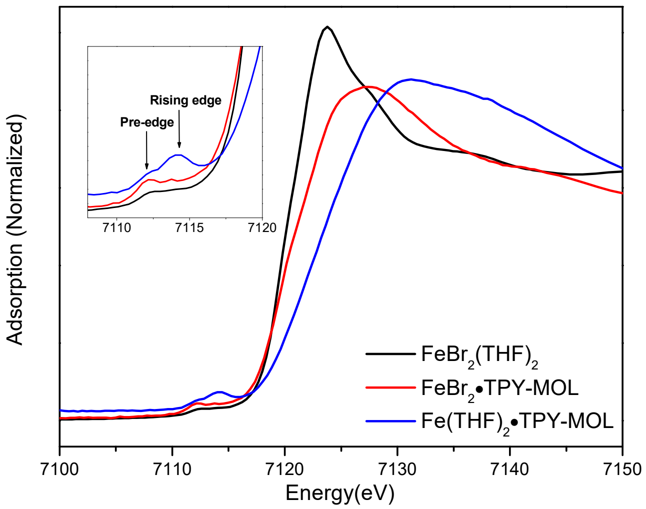

Figure S12. XANES spectra of $FeBr_2(THF)_2$, $FeBr_2$•TPY-MOL, and $Fe(THF)_2$•TPY-MOL.

The oxidation states of Fe species within MOLs were determined by comparing the energy of the pre-edge peaks to that of the reference compound $FeBr_2(THF)_2$. The positions of the pre-edge position for $FeBr_2$•TPY-MOL (7111.5 eV), $Fe(THF)_2$•TPY-MOL (7111.6 eV) align well with that of $FeBr_2(THF)_2$ (7111.5 eV) and two similar five-coordinate Fe(II) species, (^{iPr}PDI)$FeCl_2$ (7111.8) and (^{iPr}PDI)$Fe(N_2)_2$ (7111.9 eV).[6] Therefore, we assign the oxidation state of iron in all Fe-functionalized MOL materials as +2. The rising edge feature for $Fe(THF)_2$•TPY-MOL (7113.2 eV), due to the 1s to ligand π^* transitions, was also observed for (^{iPr}PDI)$Fe(N_2)_2$ (7114.0 eV).

4.4 EXAFS fitting.

Fits of the EXAFS regions were performed using the Artemis program of the IFEFFIT package. Fits were performed in *R*-space with a *k*-weight of 3 for cobalt samples and a *k*-weight of 2 for iron samples. Refinement was performed by optimizing an amplitude factor S_0^2 and energy shift ΔE_0, which are common to all paths, in addition to parameters for bond length (ΔR) and Debye-Waller factor (σ^2).

Table S1. Summary of EXAFS fitting parameters for $CoCl_2$•TPY-MOL

**Fitting range**	***k*** **3.00 – 12.30 Å^{-1}**
	R **1.0 – 5.0 Å**

**Independent points**	21
**Variables**	11
**Reduced chi-square**	20.06
**R-factor**	0.006
S_0^2	1.000
**Δ*E*₀(eV)**	5.21±1.53
R **(Co-N2) (1)**	2.09±0.01 Å
R **(Co-N1) (2)**	2.16±0.01 Å
R **(Co-Cl) (2)**	2.28±0.01 Å
R **(Co-C10) (2)**	2.96±0.01 Å
R **(Co-C11) (2)**	3.06±0.03 Å
R **(Co-C1) (2)**	3.23±0.02 Å
R **(Co-C7) (2)**	4.40±0.04 Å
R **(Co-C4) (2)**	4.45±0.04 Å
R **(Co-C2) (2)**	4.54±0.04 Å
σ^2 **(Co-N)**	0.004±0.001 $Å^2$
σ^2 **(Co-Cl)**	0.010±0.001 $Å^2$
σ^2 **(Co-C^{close})**	0.004±0.002 $Å^2$
σ^2 **(Co-C^{distal})**	0.018±0.004 $Å^2$

Table S2. Summary of EXAFS fitting parameters for Co(THF)$_2$•TPY-MOL

**Fitting range**	***k*** **3.00 – 10.00 $Å^{-1}$**<br>***R*** **1.0 – 5.0 Å**
**Independent points**	21
**Variables**	13
**Reduced chi-square**	29.81
**R-factor**	0.013
S_0^2	1.000
**ΔE_0(eV)**	1.34±2.81
R **(Co-N2) (1)**	1.81±0.02 Å
R **(Co-N1) (2)**	1.92±0.02 Å
R **(Co-O) (2)**	2.04±0.02 Å
R **(Co-C11) (4)**	2.88±0.04 Å
R **(Co-C^{THF}) (4)**	3.04±0.03 Å
R **(Co-C1) (2)**	3.13±0.09 Å

***R* (Co-C7) (2)**	3.99±0.06 Å
***R* (Co-C4) (2)**	4.04±0.09 Å
***R* (Co-C2) (2)**	4.06±0.06 Å
***R* (Co-Co) (12)**	2.51 Å
***R* (Co-Co) (6)**	3.54 Å
**σ^2 (Co-N)**	0.001±0.001 Å^2
**σ^2 (Co-O)**	0.001±0.001 Å^2
**σ^2 (Co-C^{close})**	0.006±0.008 Å^2
**σ^2 (Co-C^{distal})**	0.010±0.006 Å^2
**σ^2 (Co-Co)**	0.002 Å^2
**Fract**	0.019±0.010

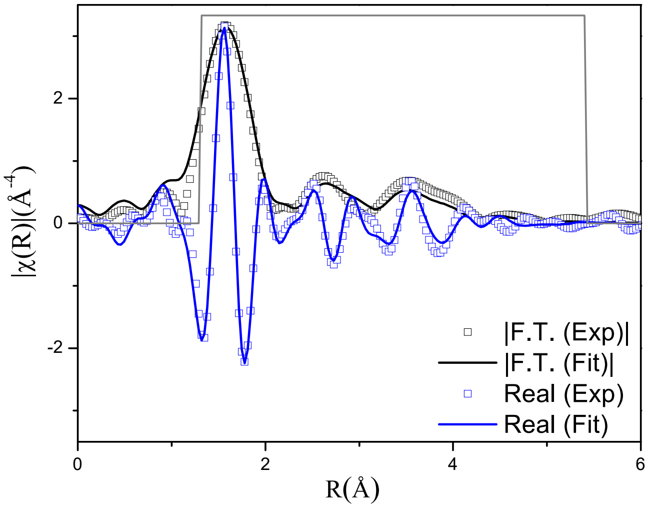

Figure S13. Experimental EXAFS spectra and fits of Co(Bpin)$_2$•TPY-MOL in R space showing the magnitude of Fourier Transform (black hollow squares, black solid line) and real components (blue hollow squares, blue solid line). The fitting range was 1.0 – 5.7 Å in R space (within the dashed lines).

Table S3. Summary of EXAFS fitting parameters for Co(Bpin)$_2$•TPY-MOL

**Fitting range**	***k* 3.10 – 10.30 Å^{-1}** ***R* 1.3 – 5.4 Å**
**Independent points**	18
**Variables**	12
**Reduced chi-square**	58.38
**R-factor**	0.023
**S_0^2**	1.000

**ΔE_0(eV)**	-3.20±1.84
***R* (Co-N2) (1)**	1.99±0.01 Å
***R* (Co-N1) (2)**	2.07±0.01 Å
***R* (Co-B) (2)**	2.01±0.06 Å
***R* (Co-C11) (4)**	2.91±0.04 Å
***R* (Co-O^{Bpin}) (4)**	3.01±0.03 Å
***R* (Co-C1) (2)**	3.21±0.08 Å
***R* (Co-C7) (4)**	4.21±0.01 Å
***R* (Co-C2) (2)**	4.33±0.01 Å
**σ^2 (Co-N)**	0.005±0.001 $Å^2$
**σ^2 (Co-B)**	0.021±0.013 $Å^2$
**σ^2 (Co-O)**	0.010±0.001 $Å^2$
**σ^2 (Co-C^{close})**	0.010±0.003 $Å^2$
**σ^2 (Co-C^{distal})**	0.018±0.015 $Å^2$

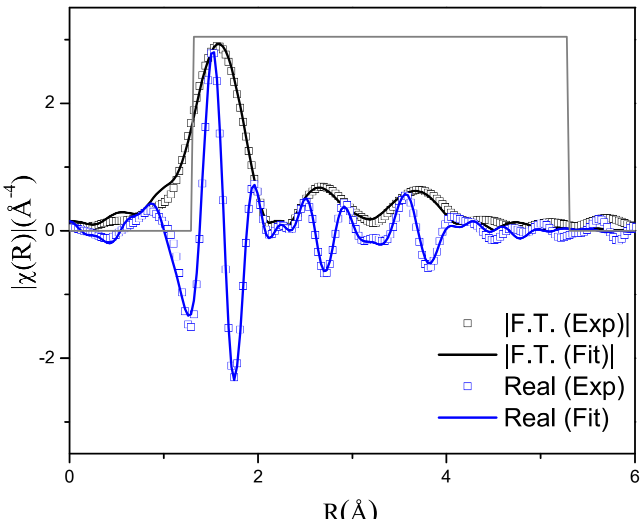

Figure S14. Experimental EXAFS spectra and fits of Co•TPY-MOL-post in R space showing the magnitude of Fourier Transform (black hollow squares, black solid line) and real components (blue hollow squares, blue solid line). The fitting range was 1.0 – 5.7 Å in R space (within the dashed lines).

Table S4. Summary of EXAFS fitting parameters for Co•TPY-MOL-post

**Fitting range**	***k* 3.20 – 10.60 $Å^{-1}$**<br>***R* 1.3 – 5.3 Å**
**Independent points**	18
**Variables**	12
**Reduced chi-square**	4.78

**R-factor**	0.010
S_0^2	1.000
**ΔE_0(eV)**	1.23±1.88
R **(Co-N2) (1)**	1.98±0.01 Å
R **(Co-N1) (2)**	2.06±0.01 Å
R **(Co-B) (2)**	2.00±0.03 Å
R **(Co-C11) (4)**	2.89±0.03 Å
R **(Co-O^{Bpin}) (4)**	3.00±0.02 Å
R **(Co-C1) (2)**	3.23±0.01 Å
R **(Co-C7) (4)**	4.21±0.05 Å
R **(Co-C2) (2)**	4.43±0.05 Å
σ^2 **(Co-N)**	0.007±0.001 $Å^2$
σ^2 **(Co-B)**	0.022±0.011 $Å^2$
σ^2 **(Co-O)**	0.009±0.004 $Å^2$
σ^2 **(Co-C^{close})**	0.012±0.006 $Å^2$
σ^2 **(Co-C^{distal})**	0.014±0.004 $Å^2$

Table S5. Summary of EXAFS fitting parameters for $FeBr_2$•TPY-MOL

**Fitting range**	***k*** **3.10 – 10.80 $Å^{-1}$**<br>***R*** **1.3 – 5.1 Å**
**Independent points**	18
**Variables**	12
**Reduced chi-square**	52.26
**R-factor**	0.011
S_0^2	1.000
**ΔE_0(eV)**	3.55±1.51
R **(Fe-N2) (1)**	2.06±0.04 Å
R **(Fe-N1) (2)**	2.22±0.01 Å
R **(Fe-Br) (2)**	2.44±0.01 Å
R **(Fe-C16) (2)**	3.09±0.02 Å
R **(Fe-C17) (2)**	3.14±0.02 Å
R **(Fe-C1) (2)**	3.39±0.06 Å
R **(Fe-C15) (2)**	4.08±0.06 Å
R **(Fe-C18) (2)**	4.21±0.06 Å
R **(Fe-C8) (2)**	4.31±0.08 Å
σ^2 **(Fe-N)**	0.004±0.005 $Å^2$

**σ^2 (Fe-Br)**	0.003±0.001 Å^2
**σ^2 (Fe-C^{close})**	0.006±0.003 Å^2
**σ^2 (Fe-C^{distal})**	0.001±0.001 Å^2

Table S6. Summary of EXAFS fitting parameters for Fe(THF)$_2$•TPY-MOL

**Fitting range**	***k* 1.50 – 10.60 Å^{-1}**<br>***R* 1.0 – 4.7 Å**
**Independent points**	21
**Variables**	14
**Reduced chi-square**	62.76
**R-factor**	0.015
**S_0^2**	1.000
**ΔE_0(eV)**	-1.94±1.05
***R* (Fe-N2) (1)**	1.94±0.02 Å
***R* (Fe-N1) (2)**	2.13±0.02 Å
***R* (Fe-O) (2)**	2.00±0.02 Å
***R* (Fe-C16) (2)**	3.12±0.04 Å
***R* (Fe-C17) (2)**	3.17±0.04 Å
***R* (Fe-C1) (2)**	3.47±0.05 Å
***R* (Fe-C^{THF}) (4)**	2.95±0.04 Å
***R* (Fe-C15) (2)**	4.34±0.09 Å
***R* (Fe-C18) (2)**	4.47±0.09 Å
***R* (Fe-C8) (2)**	4.37±0.07 Å
**σ^2 (Fe-N)**	0.001±0.001 Å^2
**σ^2 (Fe-O)**	0.010±0.003 Å^2
**σ^2 (Fe-C^{close})**	0.001±0.001 Å^2
**σ^2 (Fe-C^{distal})**	0.016±0.014 Å^2
**σ^2 (Fe-C^{THF})**	0.004±0.006 Å^2

5. X-Ray Photoelectron Spectroscopy (XPS)

XPS data were collected using the AXIS Nova spectrometer (Kratos Analytical) with monochromatic Al Kα X-ray source. The Al anode was powered at 10 mA and 15 kV. The instrument work function was calibrated to give an Au 4f$_{7/2}$ metallic gold binding energy (BE) of

83.95 eV. Instrument base pressure was *ca.* 1×10^{-9} Torr. The analysis area size was 0.3×0.7 mm^2. For calibration purposes, the binding energies were referenced to the C 1s peak at 284.8 eV. Survey spectra were collected with a step size of 1 eV and 160 eV pass energy. The Co 2p and Fe 2p spectra of $Co(THF)_2$•TPY-MOL and $Fe(THF)_2$•TPY-MOL were collected with a pass energy of 160 eV and 0.25 eV step size using 10 and 15 sweeps of 300 and 600 seconds for each sweep, respectively. The MOL samples were introduced into the XPS analysis chamber without exposing to air.

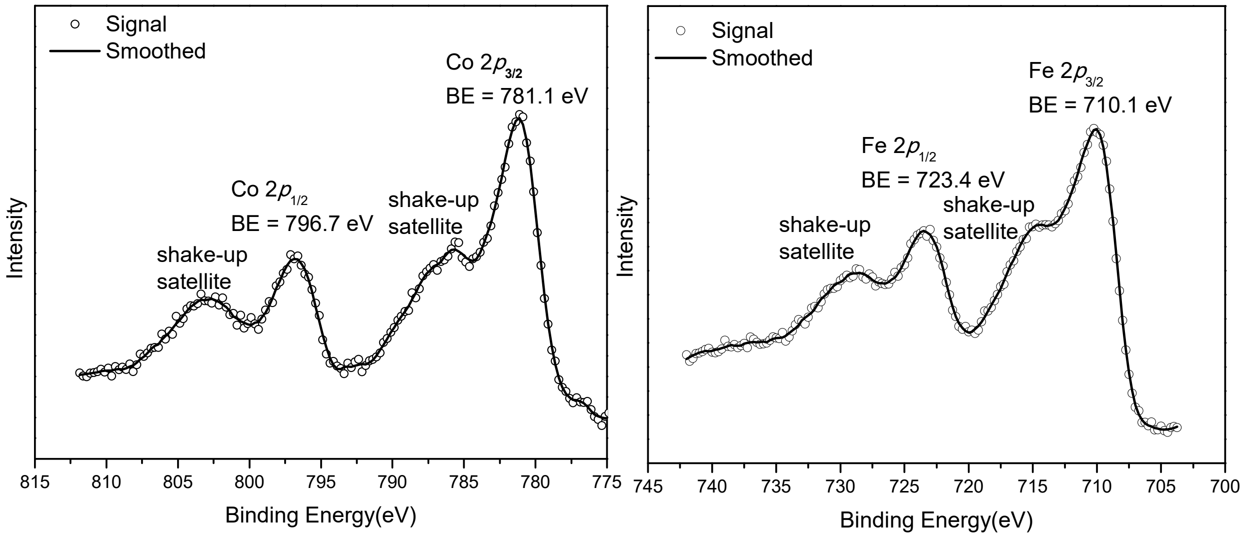

Figure S15. Co 2p and Fe 2p XPS spectra of $CoCl_2$•TPY-MOL (left) and $FeBr_2$•TPY-MOL (right).

6. Electron Paramagnetic Resonance Spectroscopy

EPR Measurements were performed using a Bruker Elexsys 500 X-band EPR spectrometer. For low temperature measurements, the samples were held at 20 K using an Oxford Systems continuous-flow He Cryostat coupled with a 10 K He stinger from Bruker. Spectra were acquired with the Bruker Win-EPR software suite. The spectrometer was equipped with a dual mode cavity, operating in the perpendicular mode.

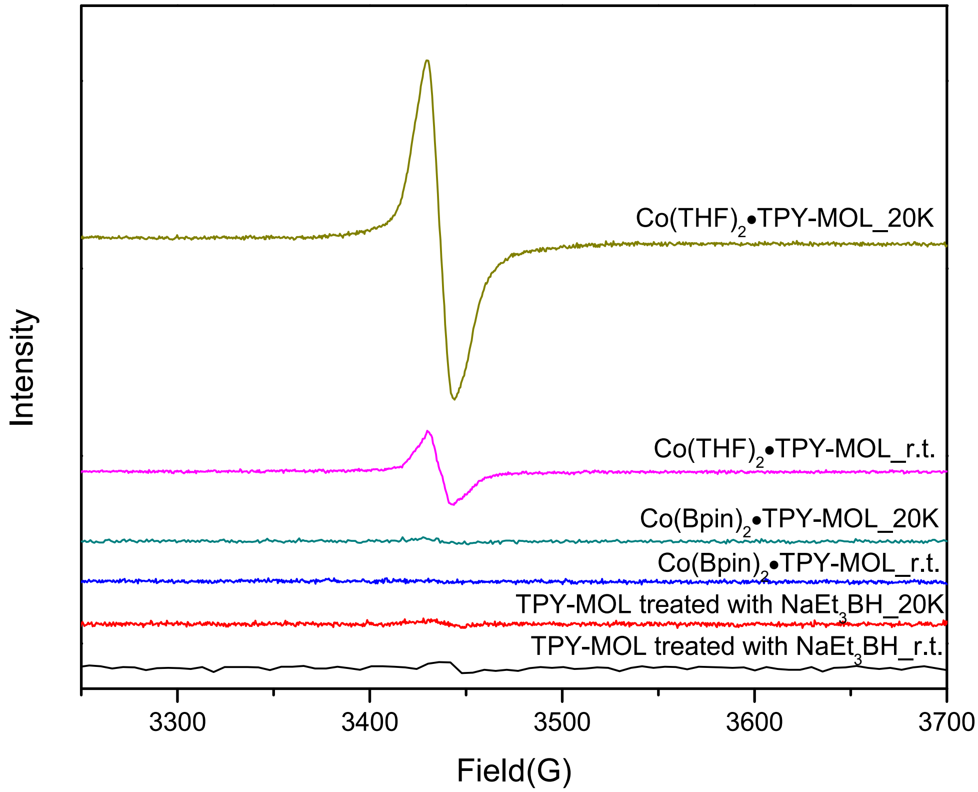

Figure S16. X-band EPR spectra of TPY-MOL treated with $NaEt_3BH$, $Co(Bpin)_2$•TPY-MOL, and $Co(THF)_2$•TPY-MOL at r.t. and 20K.

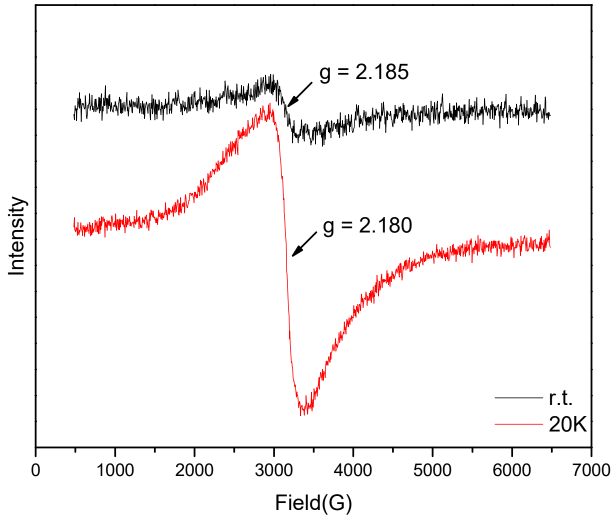

Figure S17. X-band EPR spectra of $Co(tpy)_2$ in toluene at r.t. and 20K. Microwave frequency: 9.634 GHz at r.t.; 9.632 GHz at 20K. EPR of $Co(tpy)_2$ at both r.t. and 20K gave a broad spectrum

that exhibited a g value of 2.18. This EPR spectrum is consistent with an S = 3/2 spin state assignment, similar to that reported for $(^{Ar}tpy)_2Co$.[7]

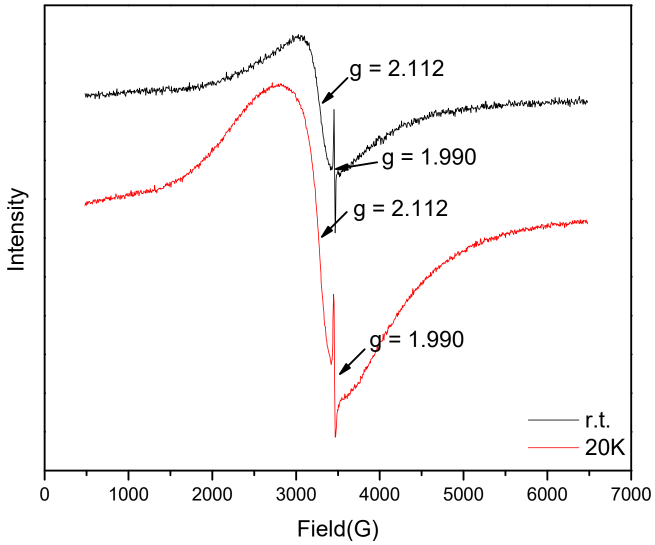

Figure S18. X-band EPR spectra of $Fe(tpy)_2$ in toluene at r.t. and 20K. Microwave frequency: 9.634 GHz at r.t.; 9.632 GHz at 20K. EPR spectra of $Fe(tpy)_2$ at r.t. and 20K gave a broad peak with g = 2.112 and another sharp peak with g = 1.990, which contradicts a previous report for $Fe(tpy)_2$.[8] We believe that the two EPR signals come from the monoanion $[Fe(tpy)_2]^-$ and the monocation $[Fe(tpy)_2]^+$ that result from the disproportionation reaction of $Fe(tpy)_2$.

7. Synthesis of Substrates

7.1 Synthesis of 5'-(4-bromobutyl)-1,1':3',1''-terphenyl

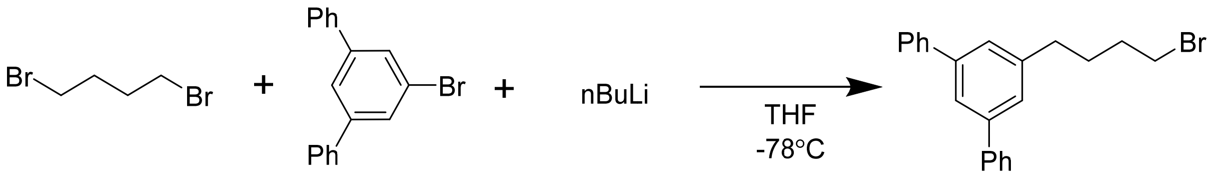

3,5-diphenyl-1-bromobenzene (909 mg, 2.9 mmol) was charged into a dry 1-neck round-bottom flask with a stir bar. The flask was degassed and placed under N_2 atmosphere. THF (20 mL) was added. The resulting solution was cooled to -78°C, and ^{n}BuLi (1.2 mL, 2.5M in hexene) was added dropwise. The solution was kept at -78°C for 40 min and added into 1,4-dibromobutane

(846 mg, 3.9 mmol, dissolved in 5 mL THF) dropwise. The solution was kept at -78°C for 2 h, and then warmed to r.t. overnight. Et_2O (40 mL) was added, and the organic layer was washed with H_2O (20 mL×2), dried over $MgSO_4$, and the solvent removed in vacuo. The resulting solid was purified by column chromatography on silica gel with 10% EtOAc/88% Hexane/2% DCM as eluent to obtain the desired product as a colorless solid (410 mg, 38% yield). ^{1}H NMR (500 MHz, $CDCl_3$): δ 7.64 (d, J = 8.0 Hz, 4H), 7.63 (s, 1H), 7.46 (t, J = 8.0 Hz, 4H), 7.39 (s, 2H), 7.37 (t, J = 8.0 Hz, 2H), 3.46 (t, J = 7.0 Hz, 2H), 2.78 (t, J = 7.5 Hz, 2H), 1.97 (m, 2H), 1.88 (m, 2H).

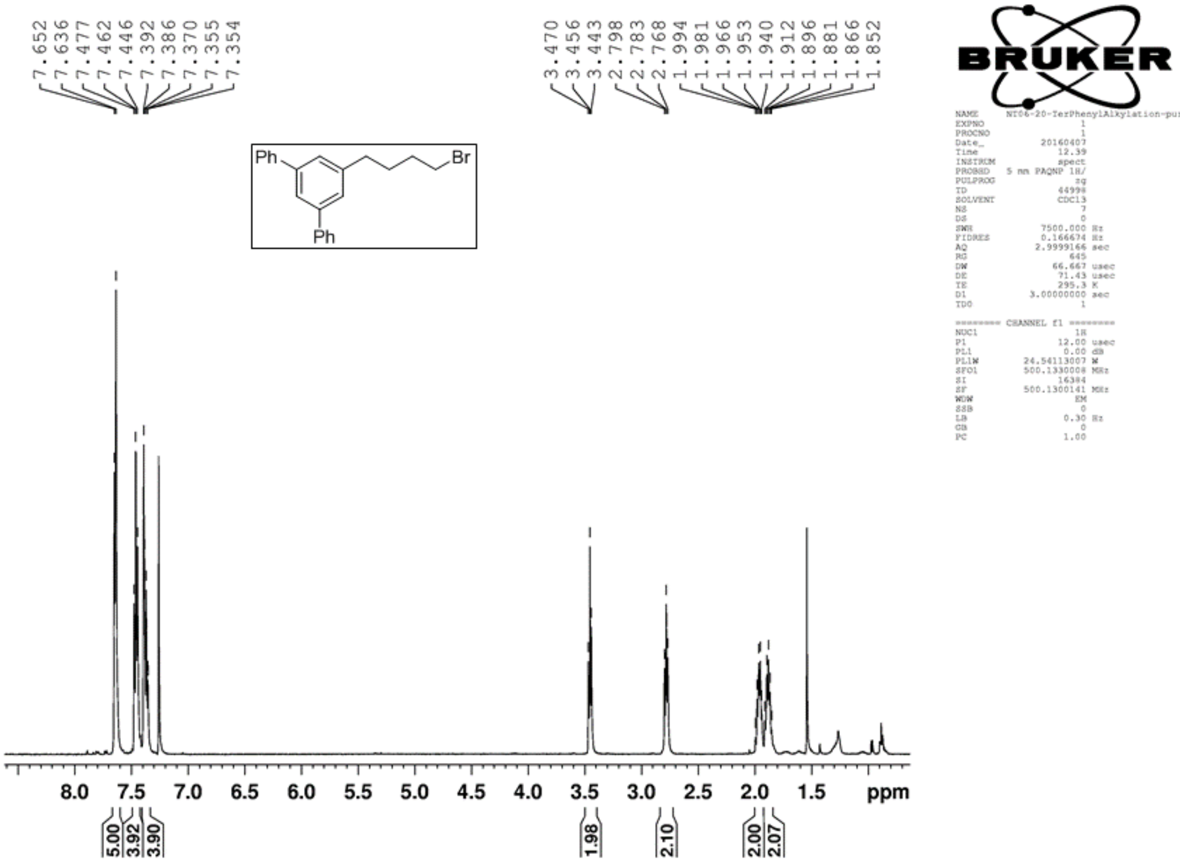

Figure S19. ^{1}H NMR of 5'-(4-bromobutyl)-1,1':3',1"-terphenyl.

7.2 Synthesis of 5'-(4-azidobutyl)-1,1':3',1"-terphenyl

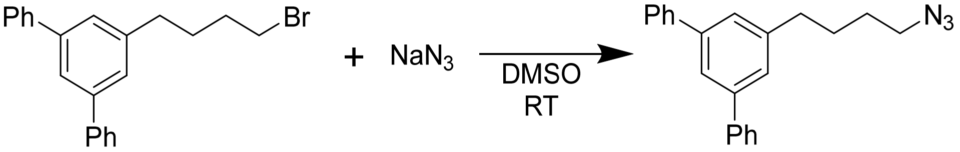

NaN_3 (107 mg, 1.65 mmol) was charged into a dry 50 mL round-bottom flask with a stir bar. The flask was evacuated and flushed with N_2. Dry DMSO (6 mL) was added, and the solution

was stirred for 2 h until the NaN_3 was completely dissolved. 5'-(4-bromobutyl)-1,1':3',1''-terphenyl (401 mg, 1.1 mmol) was dissolved in dry DMSO (2 mL) and added to the stirring solution. The solution as allowed to stir at r.t. for 2 d. The solution was added water (10 mL) dropwise followed by Et_2O (10 mL) in one portion. The organic layer was separated from the aqueous layer in a separatory funnel. The aqueous layer was further extracted with Et_2O (20 mL×3). All organics were combined, dried over Na_2SO_4, and concentrated in vacuo. The crude residue was purified with column chromatography on silica gel using 5% EtOAc/92% Hexane/3% DCM as eluent to obtain the desired product as a colorless oil (278 mg, 77% yield). ^{1}H NMR (500 MHz, $CDCl_3$): δ 7.68 (m ,5H), 7.49 (t, J = 8.0 Hz, 4H), 7.42 (s, 2H), 7.40 (t, J = 8.0 Hz, 2H), 3.34 (t, J = 7.0 Hz, 2H), 2.81 (t, J = 7.5 Hz, 2H), 1.84 (m, 2H), 1.73 (m, 2H).). ^{13}C NMR (125 MHz, $CDCl_3$): δ 142.89, 141.99, 141.29, 128.84, 127.45, 127.34, 126.36, 124.01, 51.41, 35.62, 28.62, 28.60.

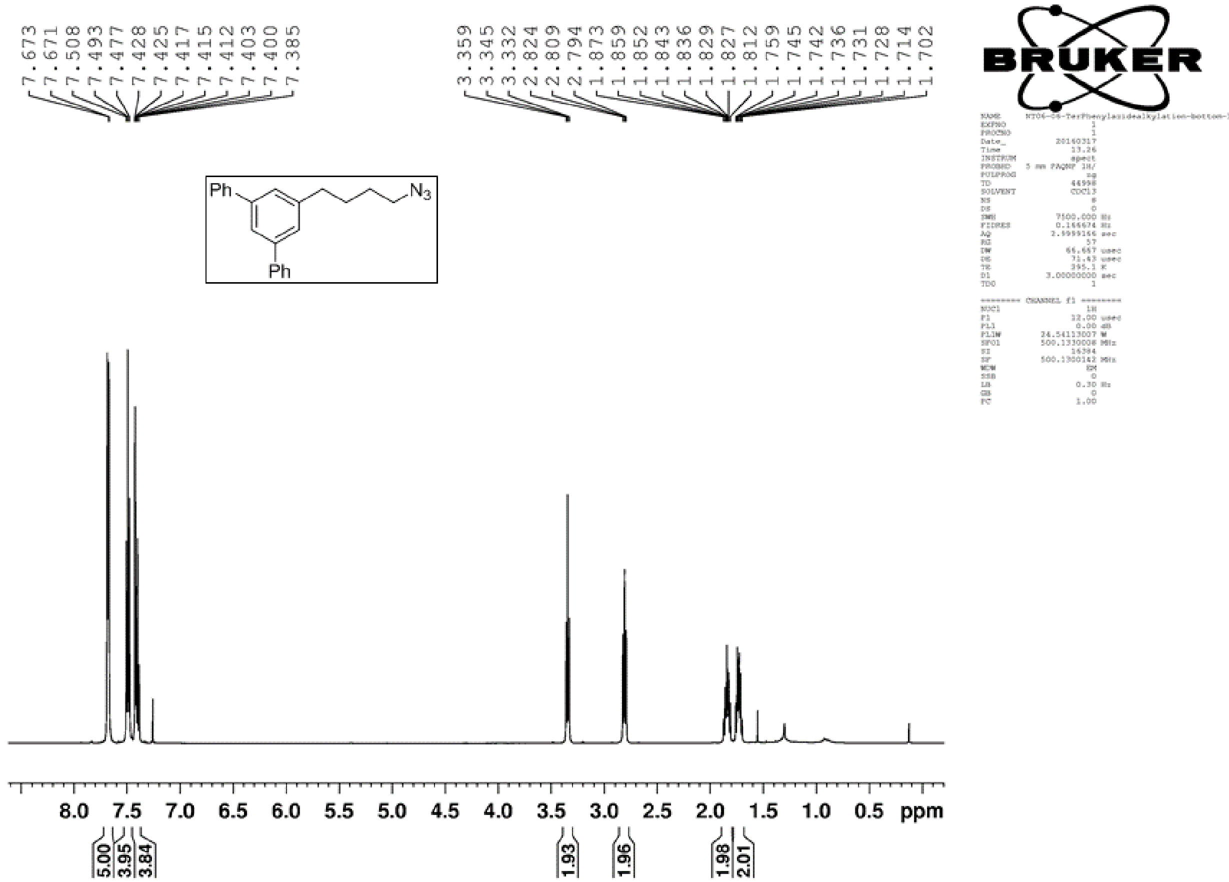

Figure S20. ^{1}H NMR of 5'-(4-azidobutyl)-1,1':3',1''-terphenyl.

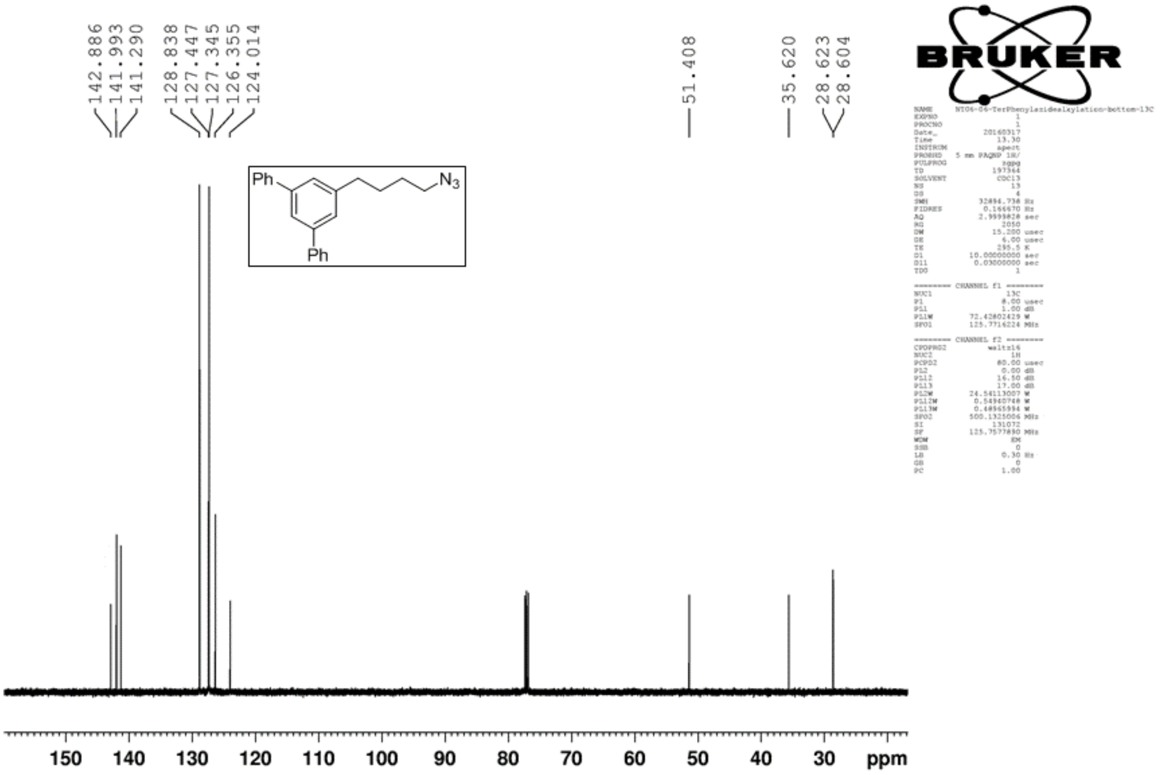

Figure S21. ^{13}C NMR of 5'-(4-azidobutyl)-1,1':3',1''-terphenyl.

8. Benzylic C-H Borylation of Arenes with Co(THF)$_2$•TPY-MOL

8.1 A typical procedure for Co(THF)$_2$•TPY-MOL catalyzed benzylic C-H active borylation of arenes (Table 2).

Co(THF)$_2$•TPY-MOL (1.5 µmol Co) was prepared as described above. After washing the MOL with *m*-xylene twice, B$_2$pin$_2$ (38.1 mg, 0.15 mmol) and *m*-xylene (1.11 mL, 9.0 mmol) were added to Co(THF)$_2$•TPY-MOL (1.5 µmol Co). The reaction mixture was stirred under nitrogen at 100 °C for 3 d. The MOL was removed from the solution by centrifugation. The supernatant was transferred to a round-bottom flask, and the MOL was washed with THF twice. The combined organic extracts were concentrated in vacuo to afford 4,4,5,5-tetramethyl-2-(3-methylbenzyl)-1,3,2-dioxaborolane (78% NMR yield based on MeNO$_2$) and 2-(3,5-dimethylphenyl)-4,4,5,5-tetramethyl-1,3,2-dioxaborolane (17% NMR yield based on MeNO$_2$).

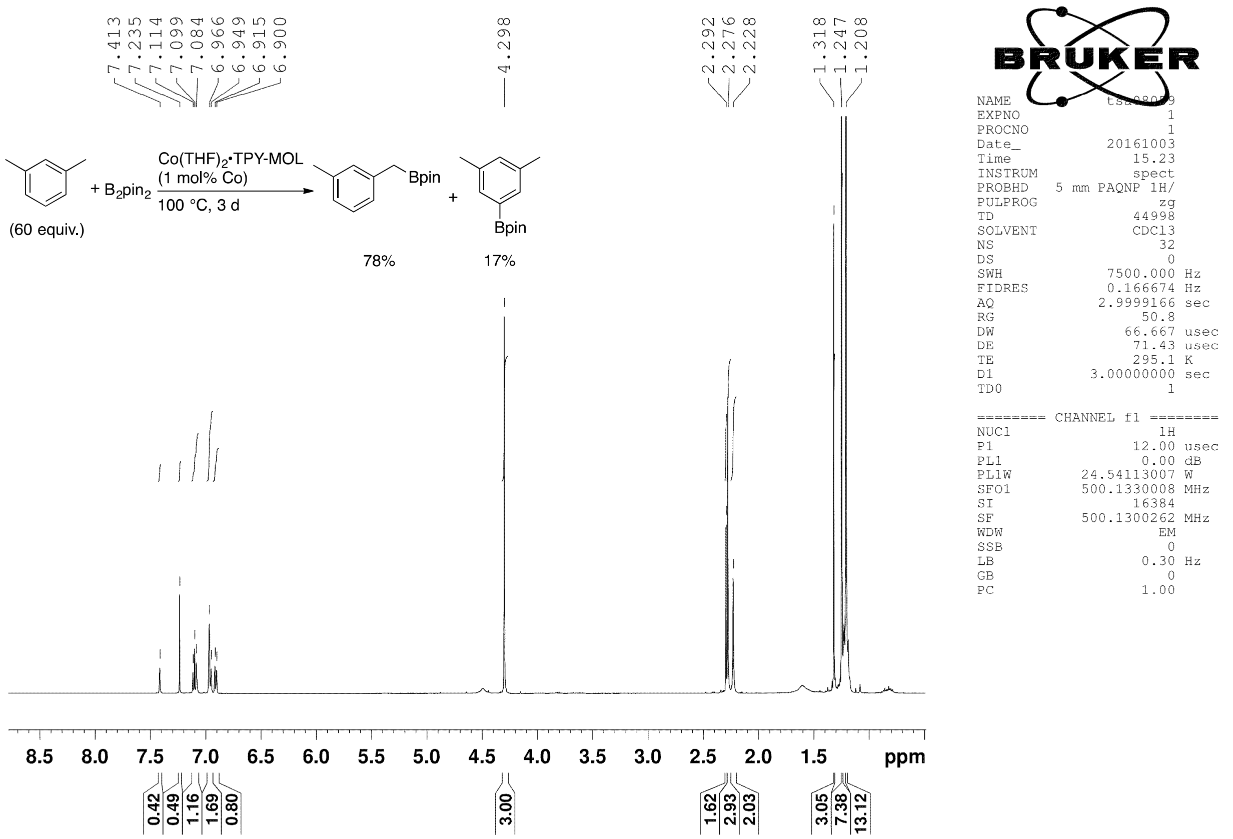

Figure S22. ^{1}H NMR spectrum (500 MHz, $CDCl_3$) of crude products from C-H borylation of m-xylene.

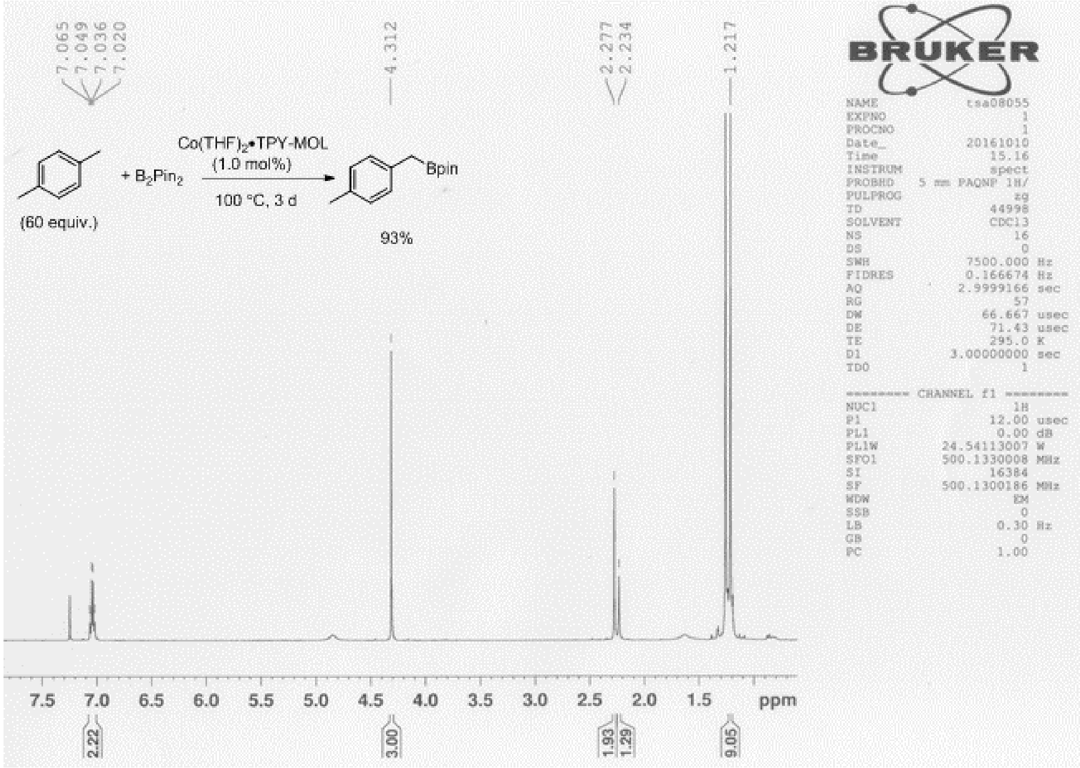

Figure S23. ^{1}H NMR spectrum (500 MHz, $CDCl_3$) of crude product from C-H borylation of p-xylene.

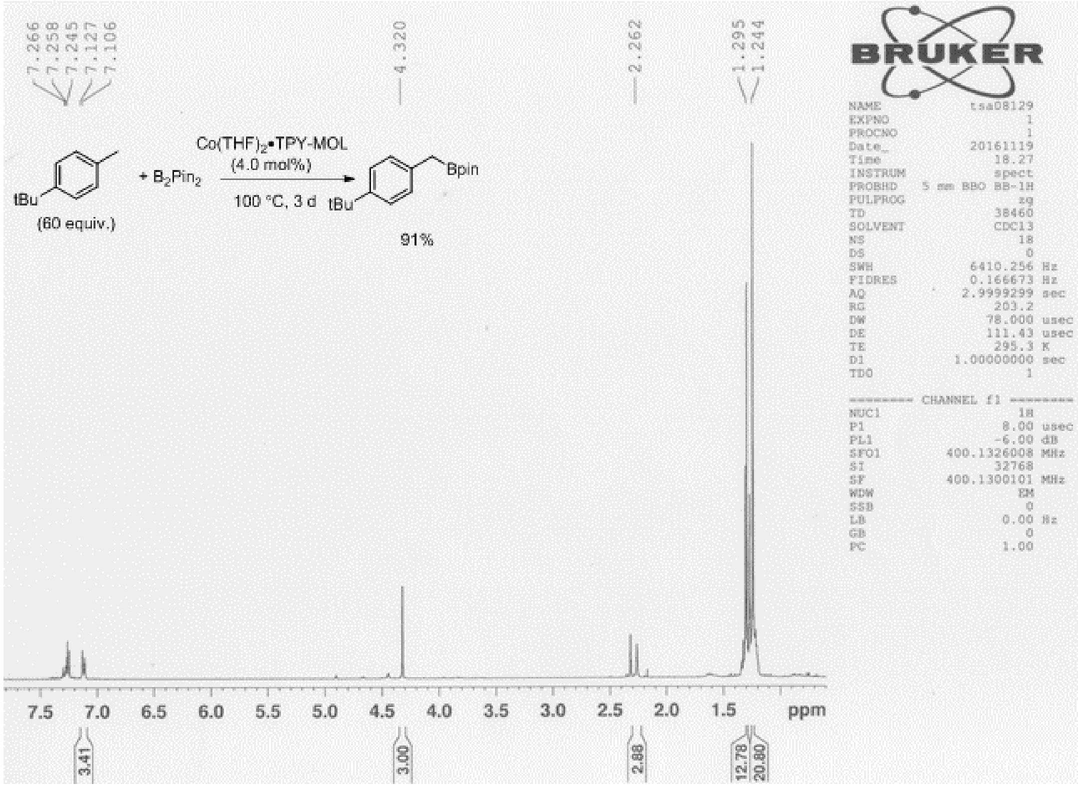

Figure S24. ^{1}H NMR spectrum (500 MHz, $CDCl_3$) of crude product from C-H borylation of 4-*tert*-butyltoluene.

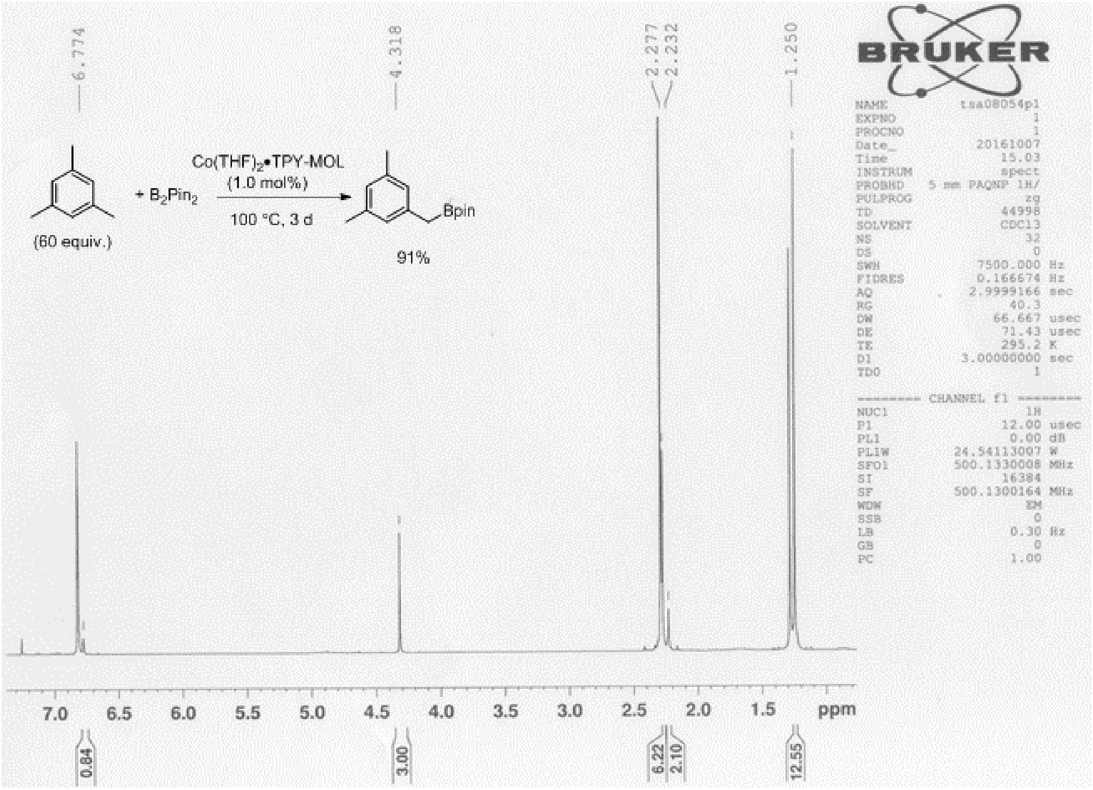

Figure S25. ^{1}H NMR spectrum (500 MHz, $CDCl_3$) of crude product from C-H borylation of mesitylene.

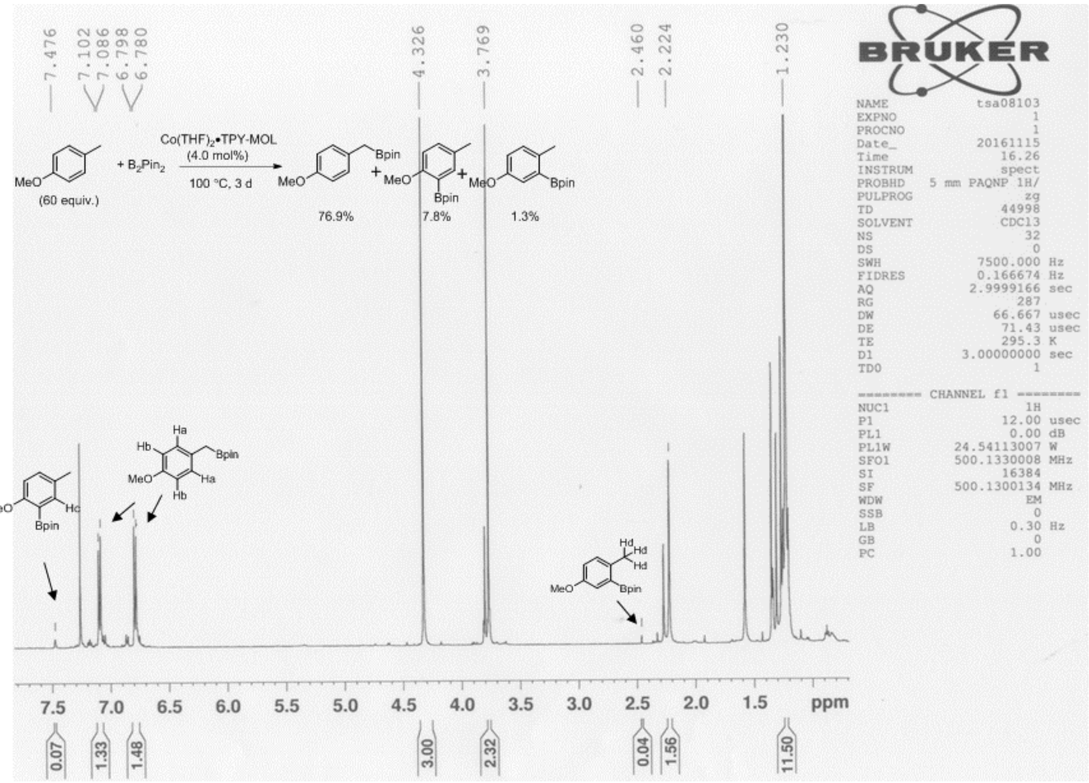

Figure S26. ^{1}H NMR spectrum (500 MHz, $CDCl_3$) of crude product from C-H borylation of 4-methylanisole.

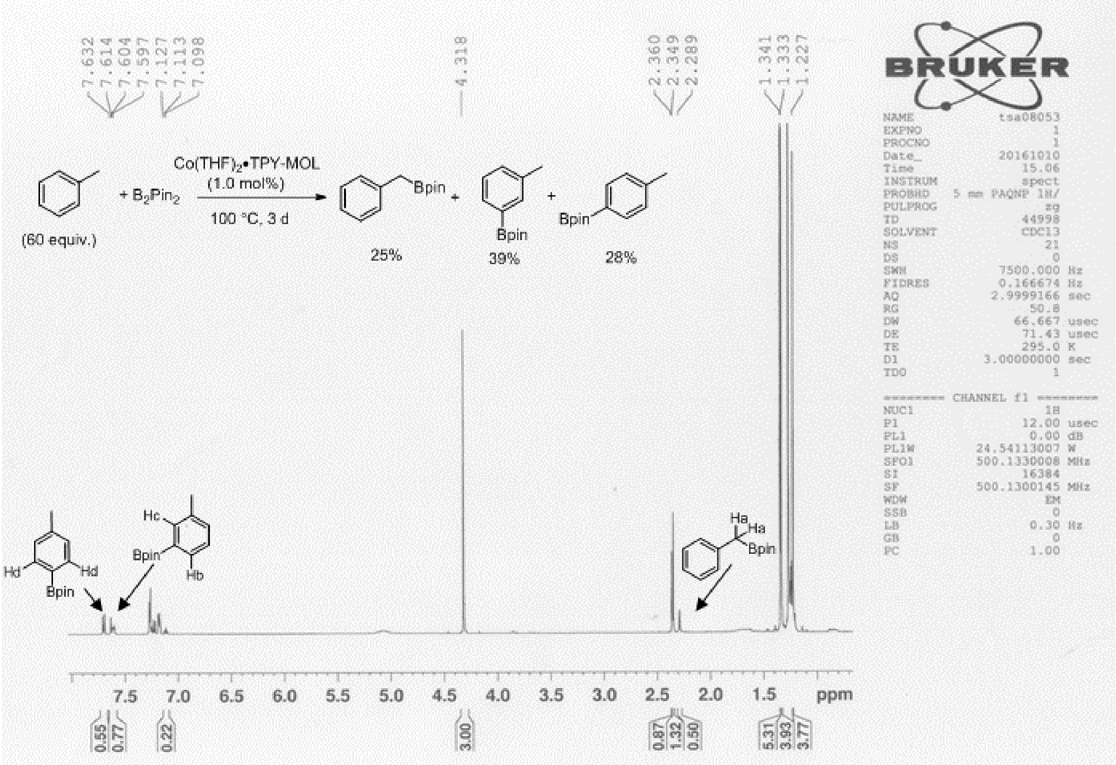

Figure S27. ^{1}H NMR spectrum (500 MHz, $CDCl_3$) of crude products from C-H borylation of toluene.

8.2 Procedures for Co(THF)$_2$•TPY-MOL catalyzed benzylic C-H active borylation of arenes (isolated yields).

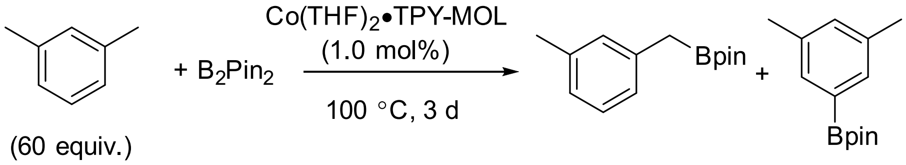

Co(THF)$_2$•TPY-MOL (4.75 µmol Co) was prepared as described above. After washing the MOL with *m*-xylene twice, B_2pin_2 (120.6 mg, 0.475 mmol) and *m*-xylene (3.52 mL, 28.5 mmol) were added to Co(THF)$_2$•TPY-MOL (4.75 µmol Co). The reaction mixture was stirred under nitrogen at 100 °C for 3 d. The MOL was removed from the solution by centrifugation. The supernatant was transferred to a round-bottom flask, and the MOL was washed with THF twice. The combined organic extracts were concentrated in vacuo and then purified by flash chromatography on silica gel (hexane/ethyl acetate = 97/3) to afford 4,4,5,5-tetramethyl-2-(3-methylbenzyl)-1,3,2-dioxaborolane (80.5 mg, 73% isolated yield) and 2-(3,5-dimethylphenyl)-4,4,5,5-tetramethyl-1,3,2-dioxaborolane (15.4 mg, 14% isolated yield).

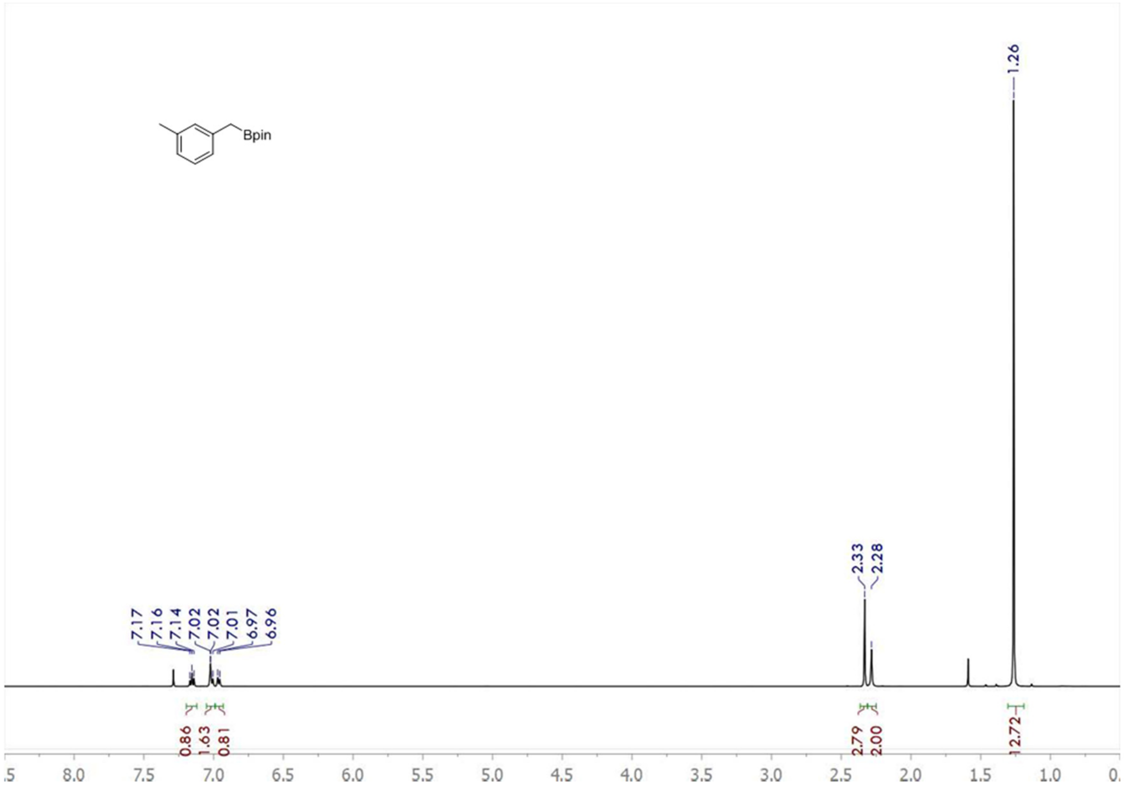

Figure S28. ^{1}H NMR spectrum (500 MHz, $CDCl_3$) of isolated 2-(3,5-dimethylphenyl)-4,4,5,5-tetramethyl-1,3,2-dioxaborolane.

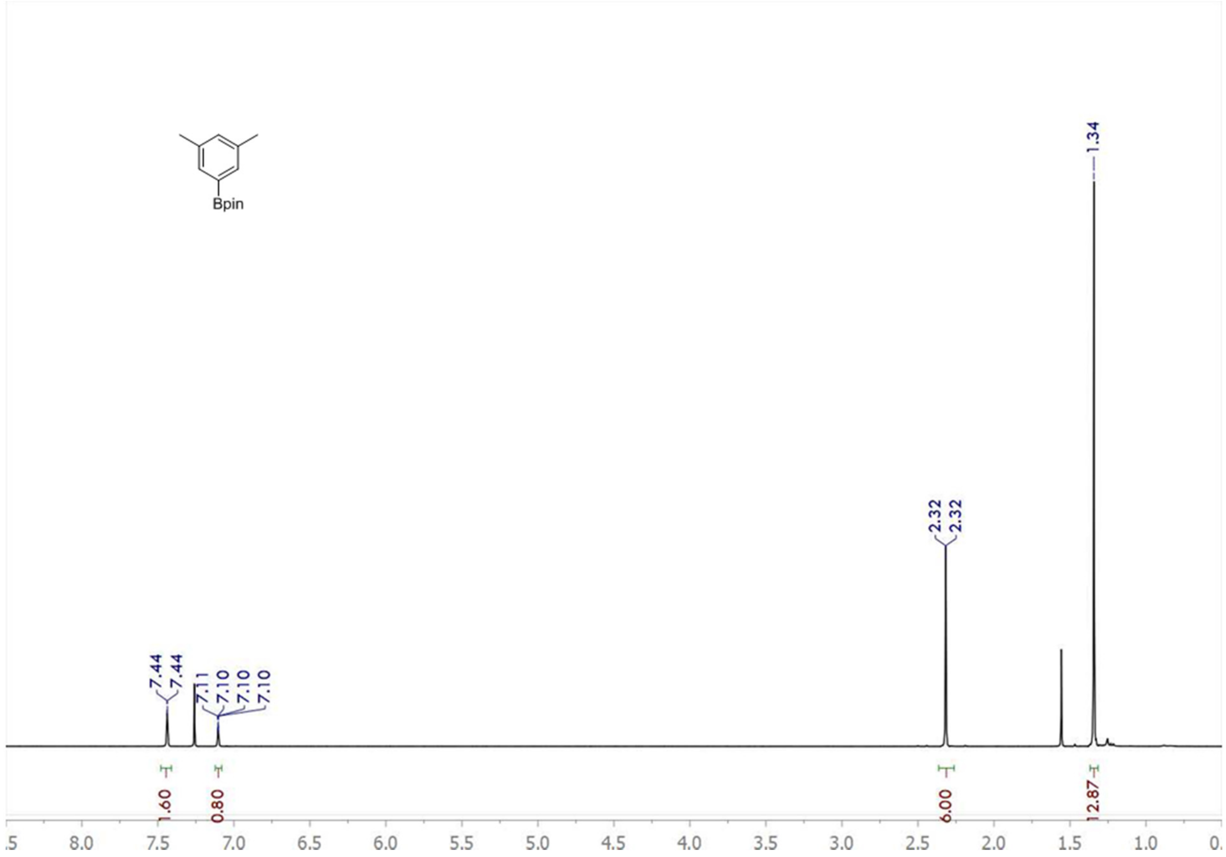

Figure S29. ^{1}H NMR spectrum (500 MHz, $CDCl_3$) of isolated 4,4,5,5-tetramethyl-2-(3-methylbenzyl)-1,3,2-dioxaborolane.

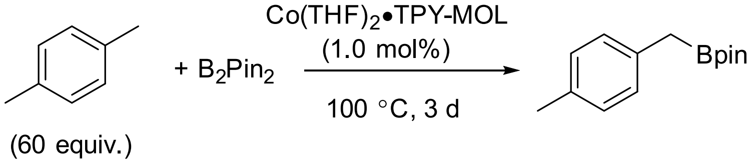

Co(THF)$_2$•TPY-MOL (4.75 μmol Co) was prepared as described above. After washing the MOL with *m*-xylene twice, B_2pin_2 (120.6 mg, 0.475 mmol) and *p*-xylene (3.52 mL, 28.5 mmol) were added to Co(THF)$_2$•TPY-MOL (4.75 μmol Co). The reaction mixture was stirred under nitrogen at 100 °C for 3 d. The MOL was removed from the solution by centrifugation. The supernatant was transferred to a round-bottom flask, and the MOL was washed with THF twice. The combined organic extracts were concentrated in vacuo and then purified by flash chromatography on silica gel (hexane/ethyl acetate = 97/3) to afford 4,4,5,5-tetramethyl-2-(4-methylbenzyl)-1,3,2-dioxaborolane (97.0 mg, 88% isolated yield)

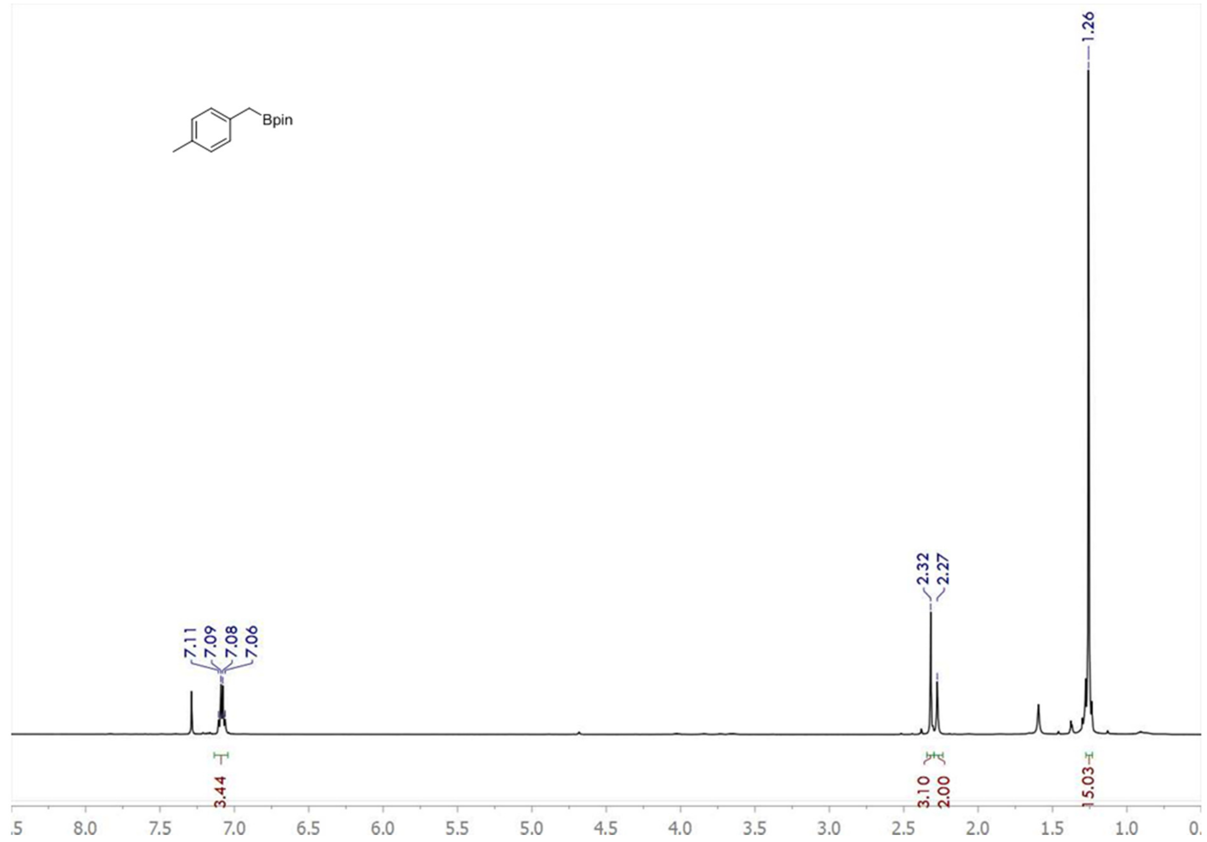

Figure S30. ^{1}H NMR spectrum (500 MHz, $CDCl_3$) of isolated 4,4,5,5-tetramethyl-2-(4-methylbenzyl)-1,3,2-dioxaborolane.

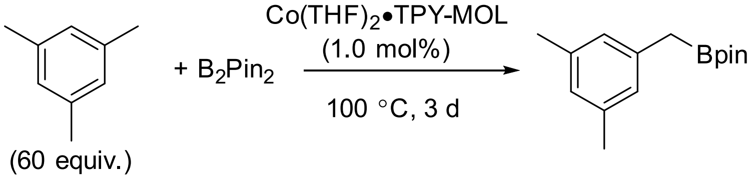

Co$(THF)_2$•TPY-MOL (4.75 μmol Co) was prepared as described above. After washing the MOL with *m*-xylene twice, B_2pin_2 (120.6 mg, 0.475 mmol) and mesitylene (3.96 mL, 28.5 mmol) were added to Co$(THF)_2$•TPY-MOL (4.75 μmol Co). The reaction mixture was stirred under nitrogen at 100 °C for 3 d. The MOL was removed from the solution by centrifugation. The supernatant was transferred to a round-bottom flask, and the MOL was washed with THF twice. The combined organic extracts were concentrated in vacuo and then purified by flash chromatography on silica gel (hexane/ethyl acetate = 97/3) to afford 4,4,5,5-tetramethyl-2-(4-methylbenzyl)-1,3,2-dioxaborolane (98.2 mg, 84% isolated yield)

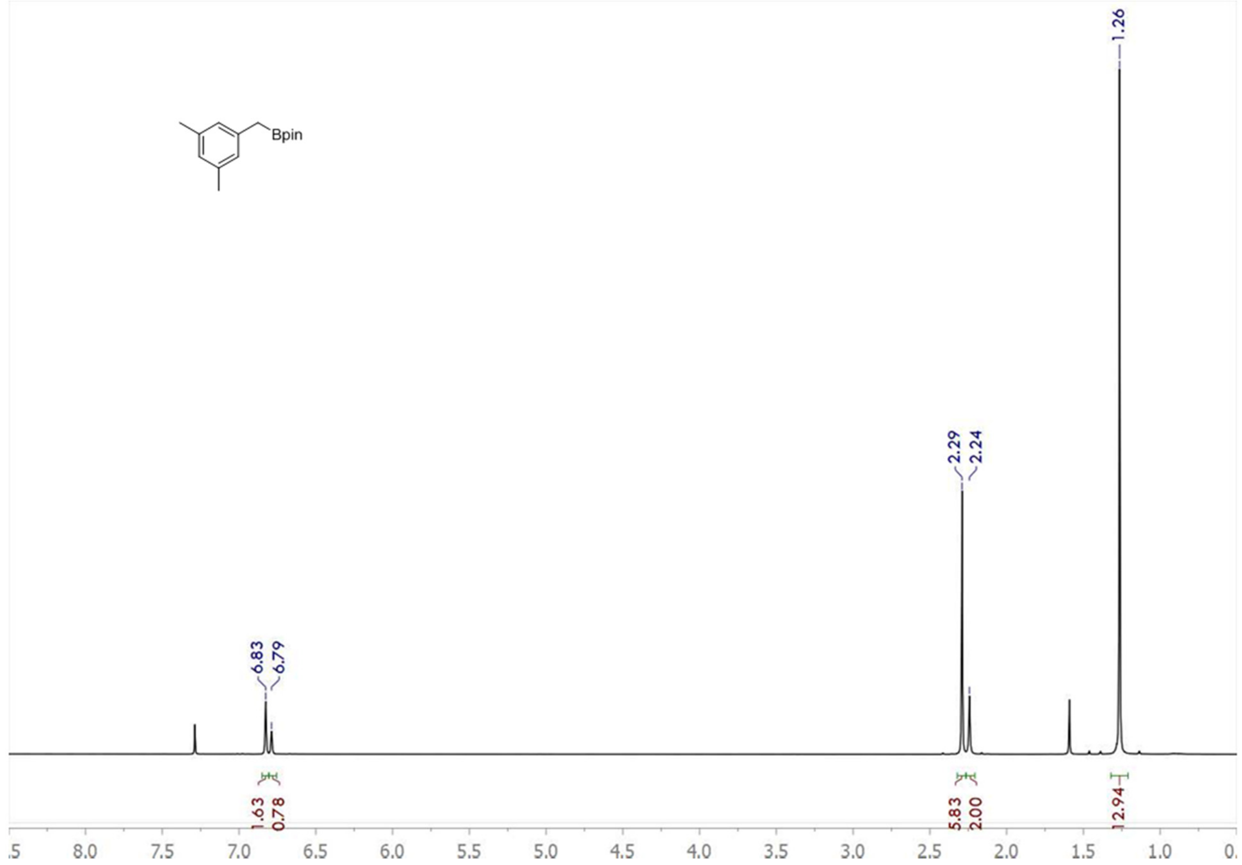

Figure S31. ^{1}H NMR spectrum (500 MHz, $CDCl_3$) of isolated 4,4,5,5-tetramethyl-2-(3,5-dimethylbenzyl)-1,3,2-dioxaborolane.

8.3 Recycling experiment

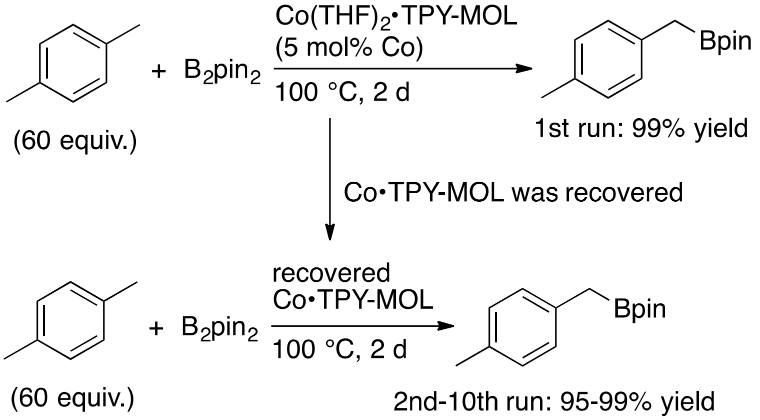

Scheme S1. Recycling of Co•TPY-MOL for the C-H active borylation of *p*-xylene with bis(pinacolate)diborane.

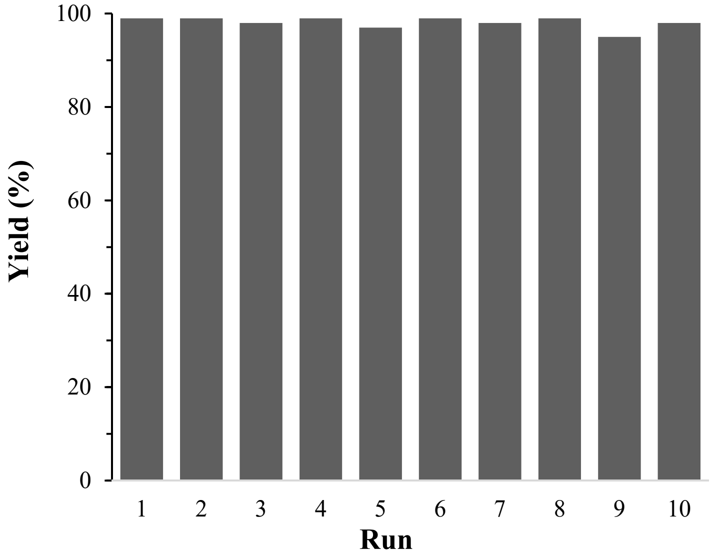

Figure S32. Plots of yields (%) of 4,4,5,5-tetramethyl-2-(4-methylbenzyl)-1,3,2-dioxaborolane at different runs in the recycling experiments of Co•TPY-MOL for C-H borylation of *p*-xylene with B_2pin_2. The Co-loadings were 5 mol%.

Co$(THF)_2$•TPY-MOL (12.3 μmol Co) was prepared as described above. After washing the MOL with *p*-xylene twice, B_2pin_2 (62.5 mg, 0.246 mmol) and *p*-xylene (1.82 mL, 14.8 mmol) were added to Co$(THF)_2$•TPY-MOL (12.3 μmol Co). The reaction mixture was stirred under nitrogen at 100 °C for 2 d. Co•TPY-MOL was recovered from the solution by centrifugation. The yield of 4,4,5,5-tetramethyl-2-(4-methylbenzyl)-1,3,2-dioxaborolane was determined by gas chromatography (99% yield). The recovered solid catalyst was used for subsequent reactions, and the reaction mixture was stirred for 2 d in each run.

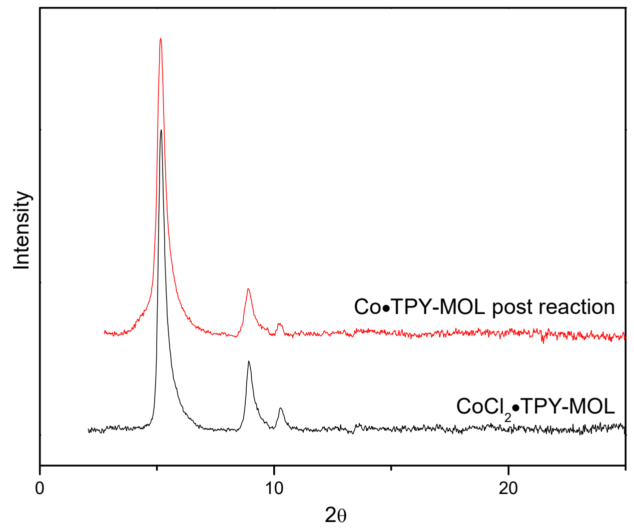

Figure S33. PXRD of Co•TPY-MOL post reaction indicates the stability of MOL catalysts under reaction conditions.

8.4 Testing the heterogeneity of Co(THF)$_2$•TPY-MOL

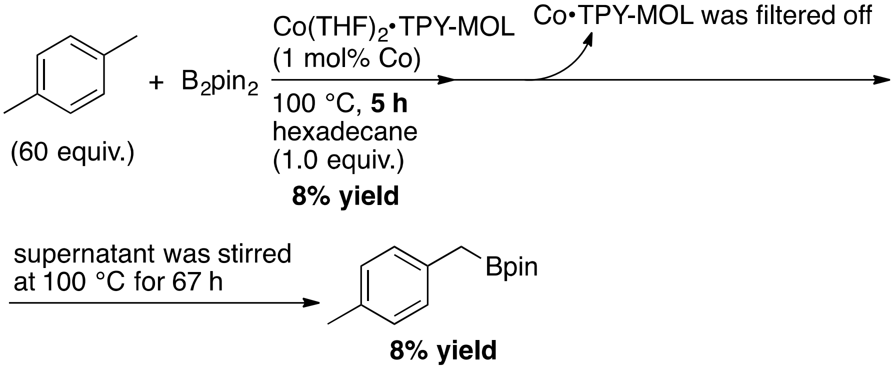

Scheme S2. Testing the heterogeneity of Co(THF)$_2$•TPY-MOL

Co(THF)$_2$•TPY-MOL (0.96 μmol Co) was prepared as described above. After washing the MOL with *p*-xylene twice, B_2pin_2 (24.4 mg, 0.096 mmol), *p*-xylene (0.711 mL, 5.76 mmol), and hexadecane (28 μL, 0.096 mmol) were added to Co(THF)$_2$•TPY-MOL (0.96 μmol Co). The reaction mixture was stirred under nitrogen at 100 °C for 5 h. The MOL was removed by centrifugation, and the supernatant was filtered through Celite. Gas chromatography (GC) of the filtrate gave a yield of 8% for 4,4,5,5-tetramethyl-2-(4-methylbenzyl)-1,3,2-dioxaborolane with hexadecane as an internal standard. The filtrate was allowed to react for an additional 67 h, but the

reaction did not proceed further (8% of 4,4,5,5-tetramethyl-2-(4-methylbenzyl)-1,3,2-dioxaborolane based on hexadecane internal standard).

8.5 Measurement of the kinetic isotope effect of C-H active borylation

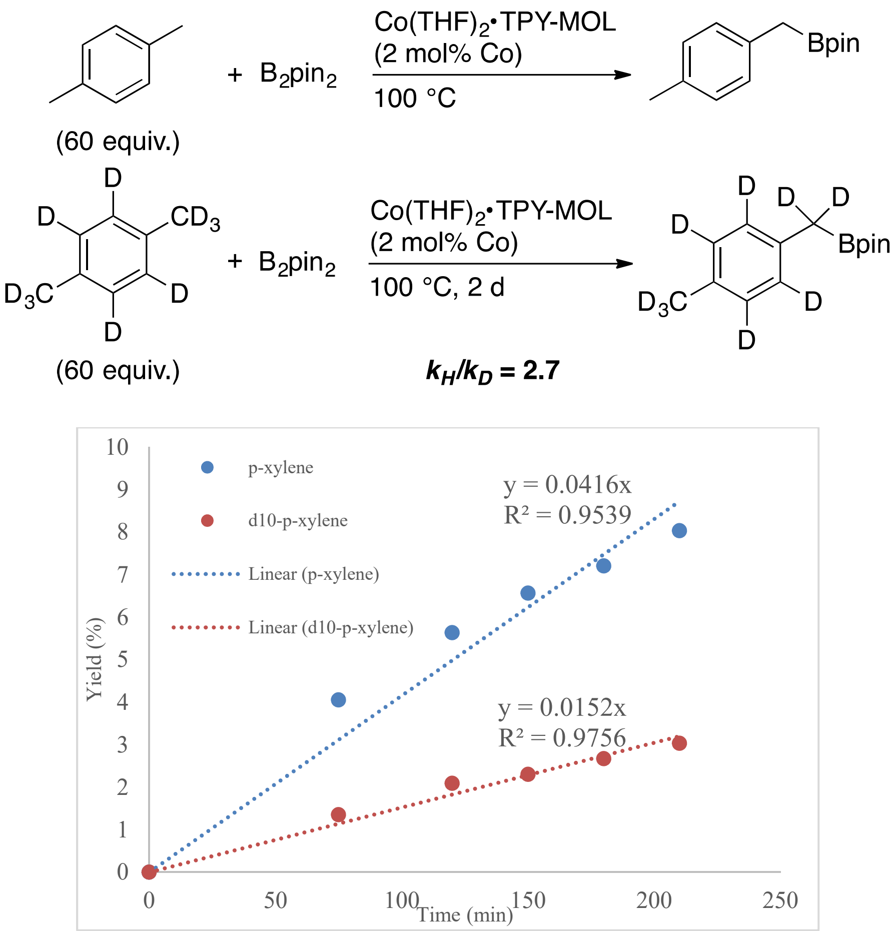

Scheme S3. Measurement of the kinetic isotope effect of C-H active borylation

Co(THF)$_2$•TPY-MOL (4.14 µmol Co) was prepared as described above. After washing the MOL with *p*-xylene or *p*-xylene-d_{10} twice, B_2pin_2 (52.6 mg, 0.207 mmol), *p*-xylene or *p*-xylene-d_{10} (1.52 mL, 12.4 mmol) and hexadecane (61 µL, 0.207 mmol) were added to Co(THF)$_2$•TPY-MOL (4.14 µmol Co). The reaction mixture was stirred under nitrogen at 100 °C. At various time points, aliquots were taken from the mixture and analyzed by gas chromatography. From the initial rates for the borylation of *p*-xylene and *p*-xylene-d_{10}, the kinetic isotope effect was determined.

Table S7. Comparison of the activity of Co(THF)$_2$•TPY-MOL with previous works[a]

Substrate	Catalyst	Catalyst Loading (mol%)	Yield (*Bn*:*Ar*)	Reference

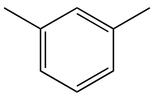	Co(THF)$_2$•TPY-MOL	1	95 (4.6:1)	this work
	Pd/C	3	79 (100:0)	9
	[Ir(OMe)(cod)]$_2$, dtbpy	2	86 (89:11)	10
	[Ir]	2	57 (45:1)	11
	UiO-Co	0.2	92 (96:4)	12
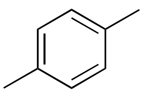	Co(THF)$_2$•TPY-MOL	1	93 (100:0)	this work
	RhCl(P^{i}Pr$_3$)$_2$(N$_2$)	1	42 (98:2)	13
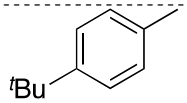	Co(THF)$_2$•TPY-MOL	1	91 (100:0)	this work
	Co(O$_2$CR)$_2$(^{Cy}ADI)	30	38 (100:0)	14
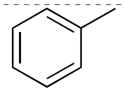	Co(THF)$_2$•TPY-MOL	1	92 (0.91:2.4)	this work
	Ni(cod)$_2$, ICy•HCl, NaO^{t}Bu	3	65 (37:63)	15

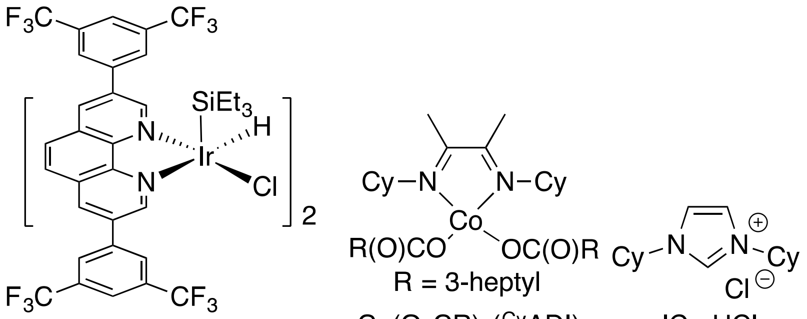

[Ir] Co(O$_2$CR)$_2$(^{Cy}ADI) ICy•HCl

dtbpy = 4,4'-di-*tert*-butylbipyridine, cod = cyclooctadiene

[a]The catalytic productivity does not necessarily reflect the catalytic activity of various catalysts.

9. Fe-catalyzed Amination

9.1 A typical procedure of Fe(THF)$_2$•TPY-MOL catalyzed C$_{sp}$^{3} C-H amination

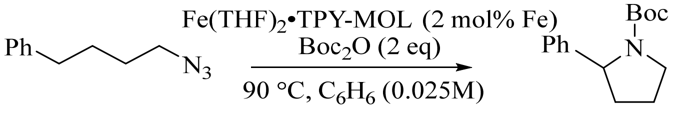

Inside a nitrogen filled glovebox, Fe(THF)$_2$•TPY-MOL (2.3 μmol, suspended in 1.0 mL benzene) was added 1-azido-4-phenylbutane (**1a**) (20.15 mg, 115 μmol) dissolved in 1.3 mL benzene. Boc$_2$O (52.8 μL, 231 μmol) was added, and the flask was sealed with a glass stopper. After removing the reaction flask from the glovebox, the sidearm was connected to a Schlenk line and subjected to vacuum then backfilled with N$_2$ five times. The sidearm was then opened to positive N$_2$ flow [IMPORTANT as CO$_2$ is released throughout the reaction], and the reaction flask

was heated at 90 °C with gentle stirring for 48 h. After cooling to r.t., MOL was separated by centrifugation, and the product was extracted from the MOL with THF washes (4 x 1 mL). The combined organic extracts were concentrated on a rotary evaporator. The residue was added nitromethane (6.17 µL) as an internal standard and the yield was determined by ^{1}H NMR in $CDCl_3$. The ^{1}H NMR data for peaks corresponding to desired product matched those of the literature report.[16]

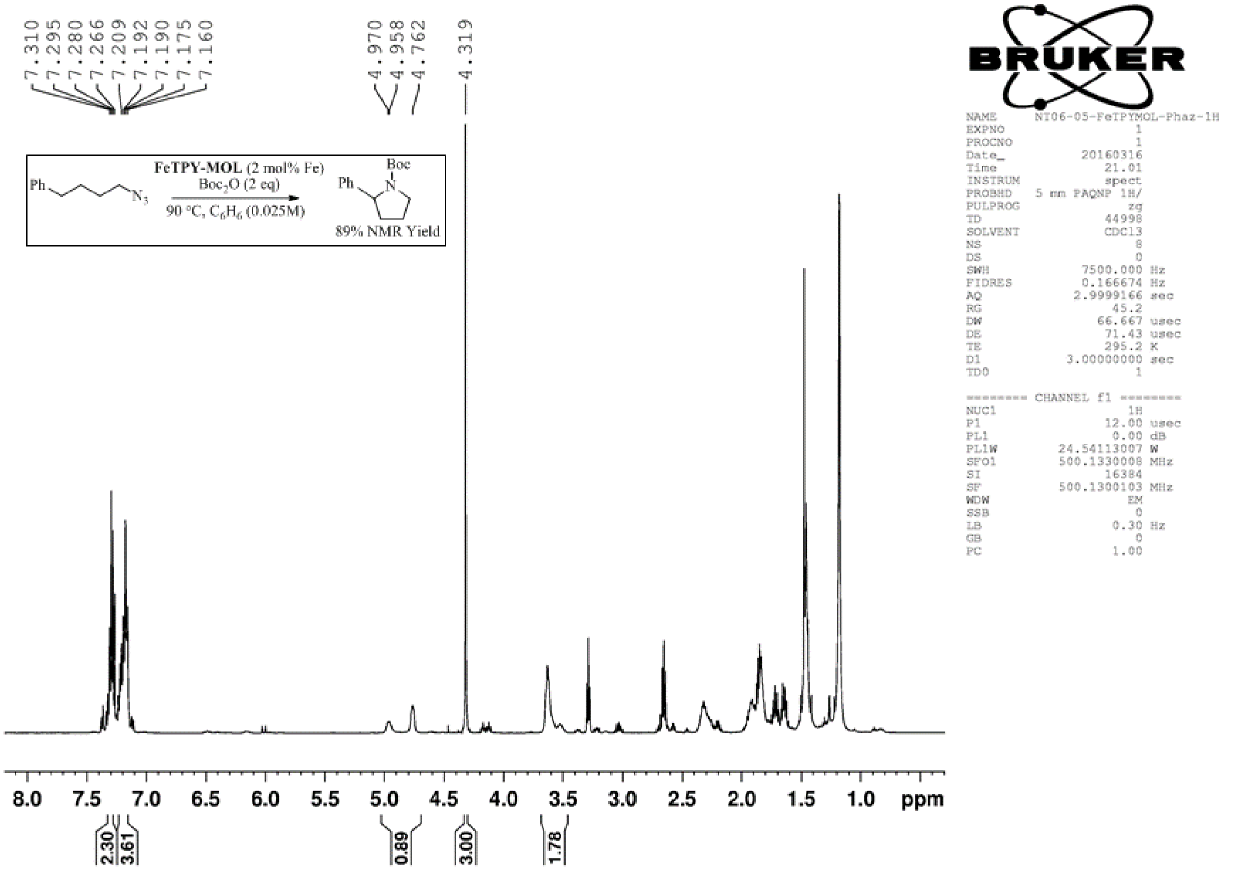

Figure S34. ^{1}H NMR spectrum (500 MHz, $CDCl_3$) of crude tert-butyl 2-phenylpyrrolidine-1-carboxylate.

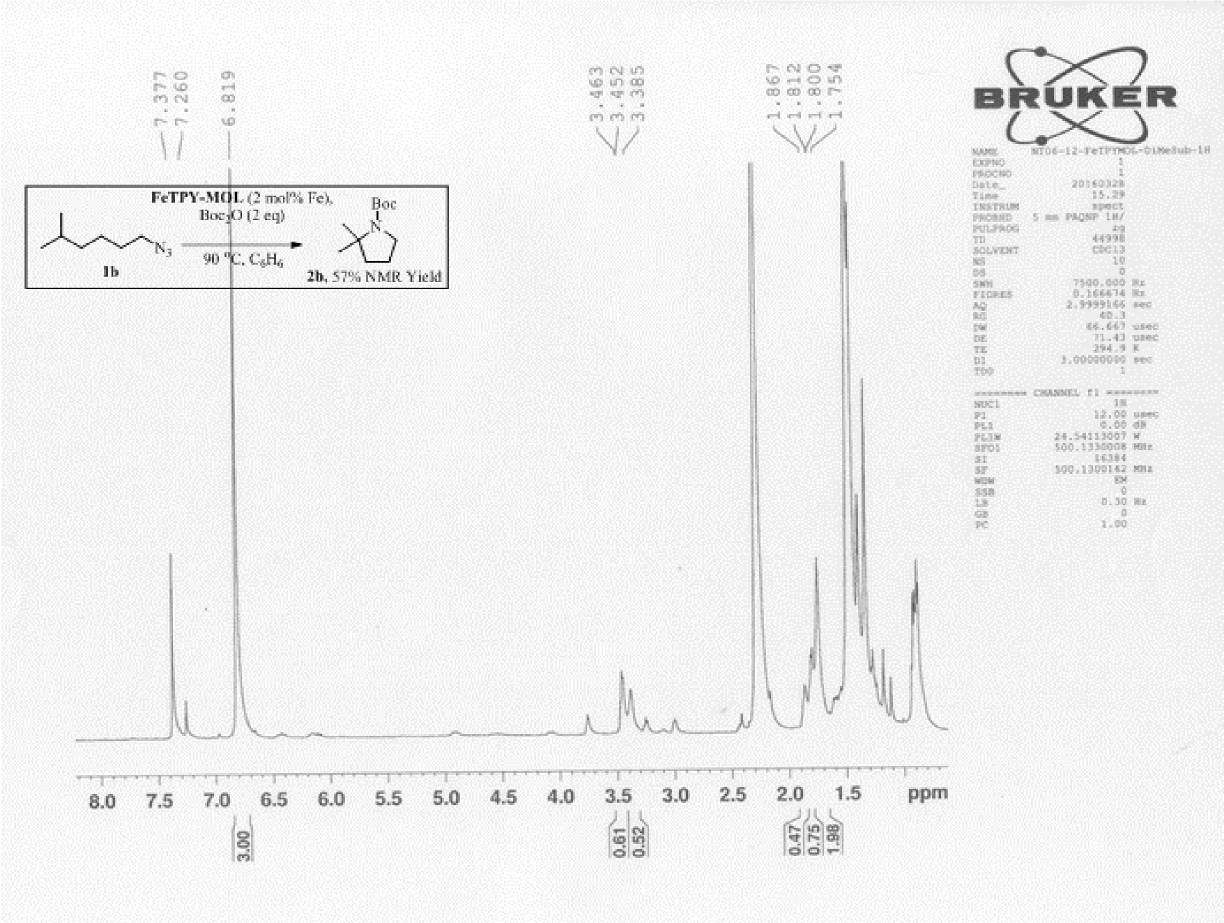

Figure S35. ^{1}H NMR spectrum (500 MHz, $CDCl_3$) of crude tert-butyl 2,2-dimethylpyrrolidine-1-carboxylate.

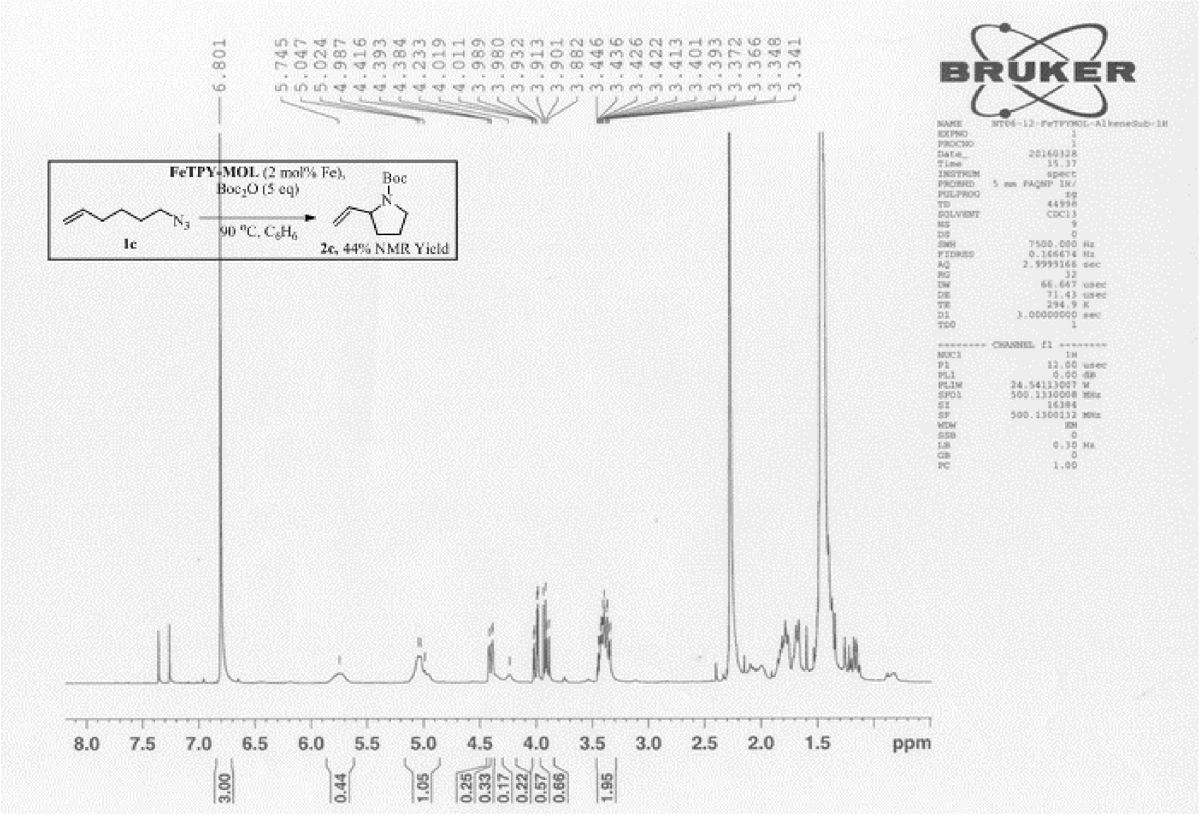

Figure S36. ^{1}H NMR spectrum (500 MHz, $CDCl_3$) of crude tert-butyl 2-vinylpyrrolidine-1-carboxylate.

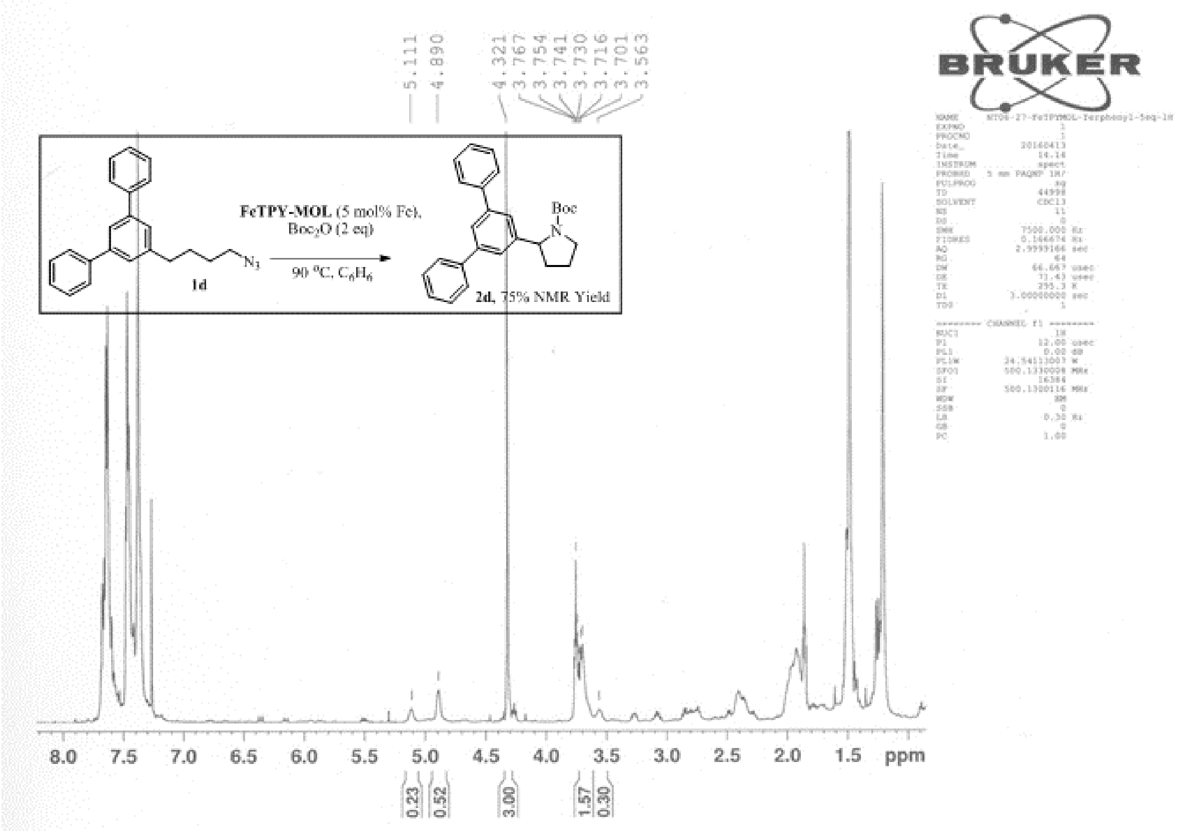

Figure S37. ^{1}H NMR spectrum (500 MHz, $CDCl_3$) of crude tert-butyl 2-([1,1':3',1"-terphenyl]-5'-yl) pyrrolidine-1-carboxylate.

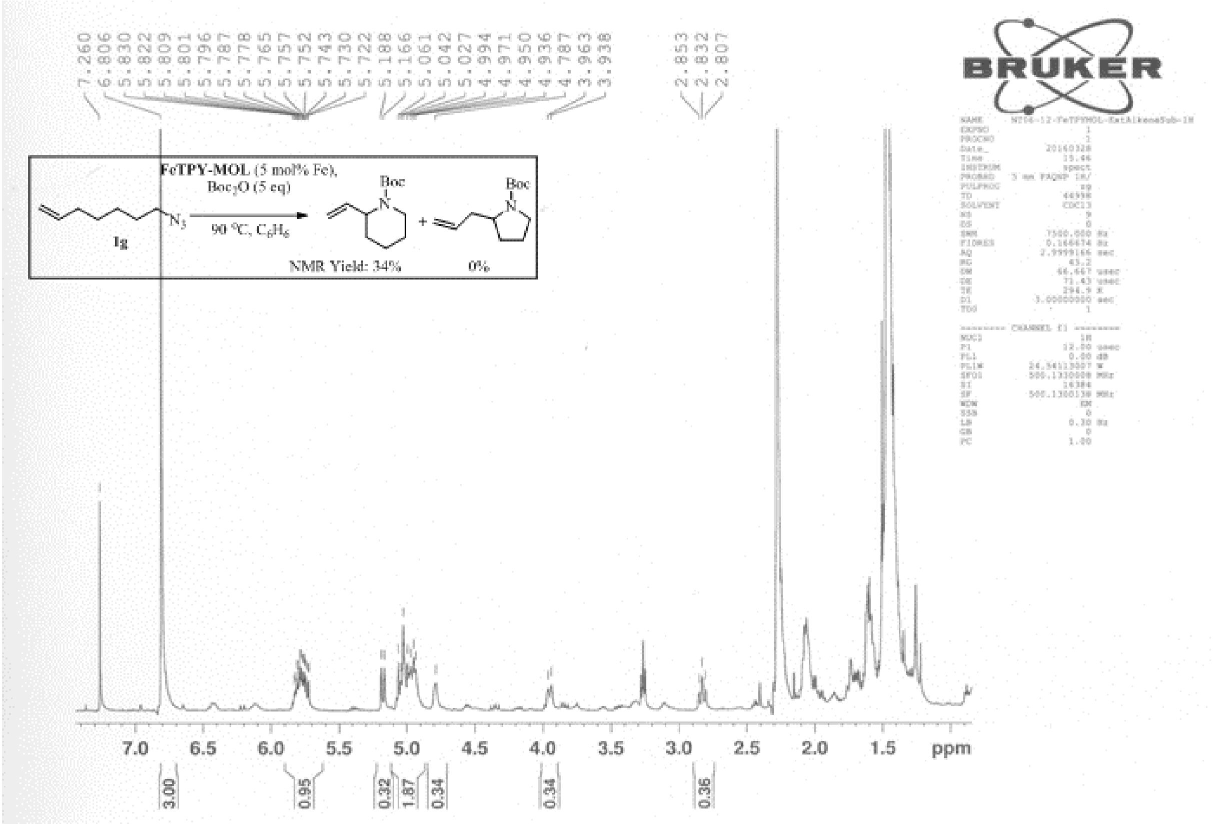

Figure S38. ^{1}H NMR spectrum (500 MHz, $CDCl_3$) of crude tert-butyl 2-vinylpiperidine-1-carboxylate.

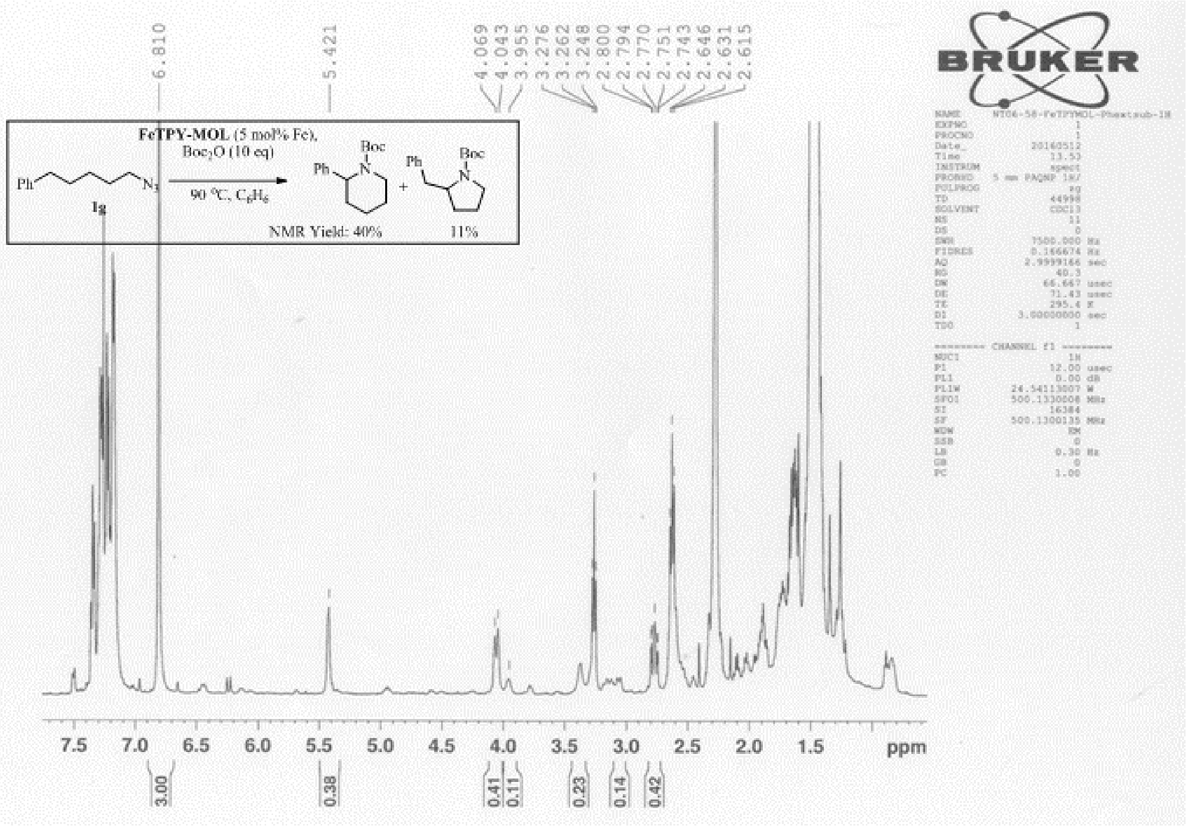

Figure S39. ^{1}H NMR spectrum (500 MHz, $CDCl_3$) of crude tert-butyl 2-phenylpiperidine-1-carboxylate and tert-butyl 2-benzylpyrrolidine-1-carboxylate.

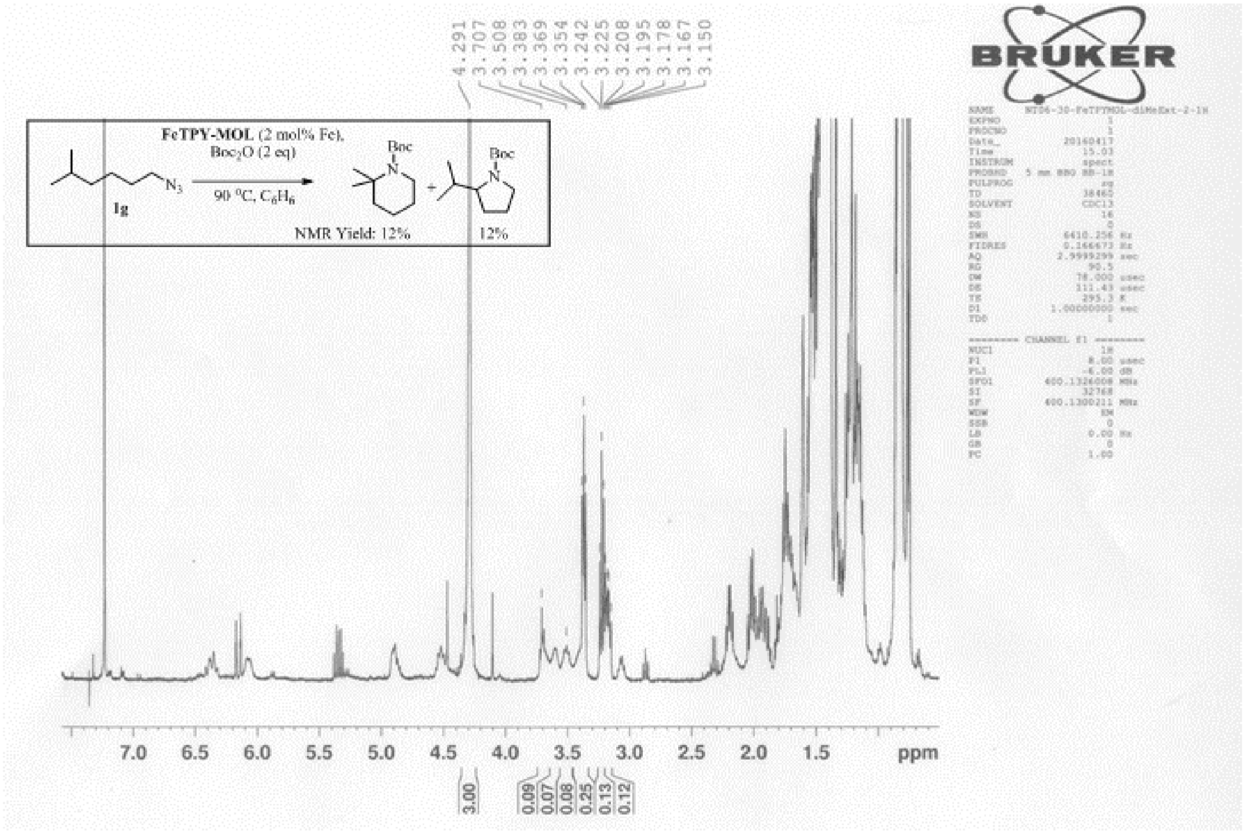

Figure S40. ^{1}H NMR spectrum (500 MHz, $CDCl_3$) of crude tert-butyl 2,2-dimethylpiperidine-1-carboxylate and tert-butyl 2isopropylpyrrolidine-1-carboxylate.

9.2 Procedures of $Fe(THF)_2$•TPY-MOL catalyzed $C_{sp}{}^3$ C-H amination reactions (isolated yields)

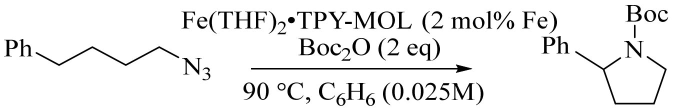

Inside a nitrogen-filled glovebox, $Fe(THF)_2$•TPY-MOL (4.75 μmol, suspended in 2.1 mL benzene) was added 1-azido-4-phenylbutane (**1a**) (41.6 mg, 238 μmol) dissolved in 2.7 mL benzene. Boc_2O (109 μL, 477 μmol) was added, and the flask was sealed with a glass stopper. After removing the reaction flask from the glovebox, the sidearm was connected to a Schlenk line and subjected to vacuum then backfilled with N_2 five times. The sidearm was then opened to positive N_2 flow [IMPORTANT as CO_2 is released throughout the reaction], and the reaction flask was heated at 90 °C with gentle stirring for 48 h. After cooling to r.t., MOL was separated by centrifugation, and the product was extracted from the MOL with THF washes (4 x 1 mL). The

combined organic extracts were concentrated on a rotary evaporator. Crude reaction mixture was then subjected to flash chromatography on silica gel using dichloromethane / methanol as eluent (being eluting with 99:1 and gradually increase to 95:5 dichloromethane / methanol) affording tert-butyl-2-phenylpyrrolidine-1-carboxylate (**2a)** as a colorless oil (49.2 mg, 84%). The ^{1}H NMR data of **2a** is indistinguishable from reported spectra.[16]

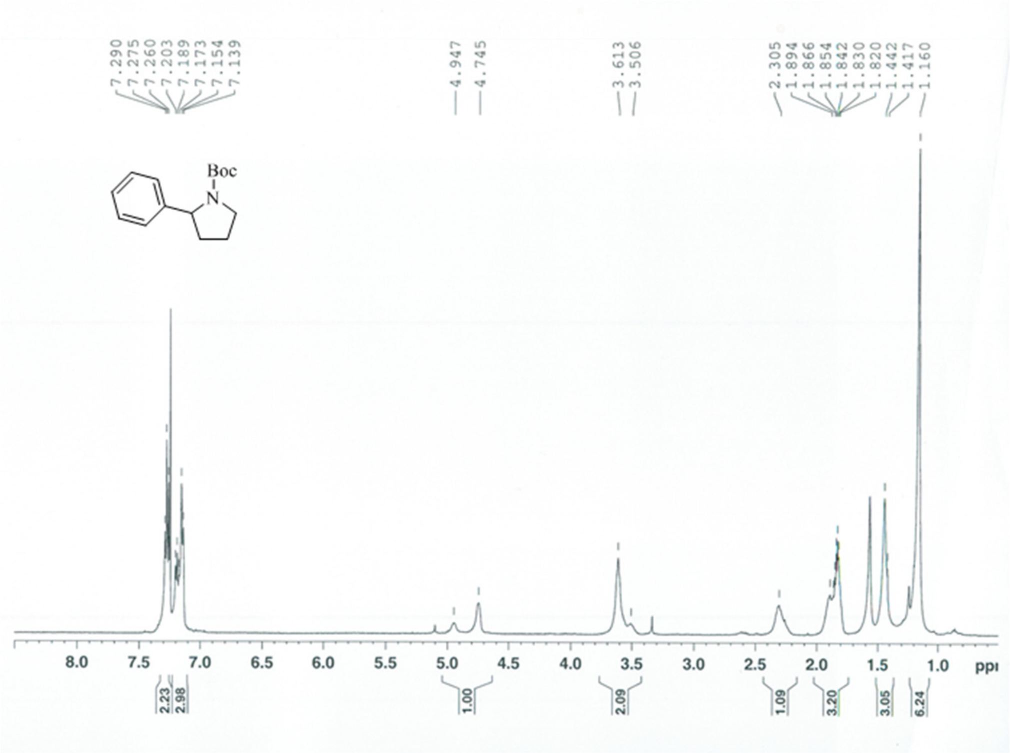

Figure S41. ^{1}H NMR spectrum (500 MHz, $CDCl_3$) of isolated **2a**.

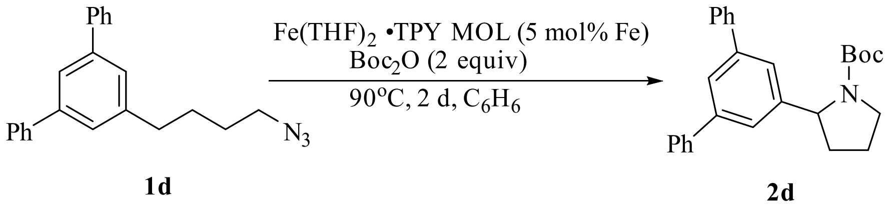

Inside a nitrogen filled glovebox, Fe(THF)$_2$•TPY-MOL (5.75 µmol, suspended in 2.5 mL benzene) was added 5'-(4-azidobutyl)-1,1':3',1"-terphenyl (**1d**) (37.65 mg, 115 µmol) dissolved in 1.3 mL benzene. Boc_2O (52.8 µL, 231 µmol) was added, and the flask was sealed with a glass stopper. After removing the reaction flask from the glovebox, the sidearm was connected to a Schlenk line and subjected to vacuum then backfilled with N_2 five times. The sidearm was then opened to positive N_2 flow [IMPORTANT as CO_2 is released throughout the reaction], and the

reaction flask was heated at 90 °C with gentle stirring for 48 h. After cooling to r.t., MOL was separated by centrifugation, and the product was extracted from the MOL with THF washes (4 x 1 mL). The combined organic extracts were concentrated on a rotary evaporator. Crude reaction mixture was then subjected to flash chromatography on silica gel using dichloromethane / methanol as eluent (being eluting with 99:1 and gradually increase to 95:5 dichloromethane / methanol) affording tert-butyl 2-([1,1':3',1"-terphenyl]-5'-yl) pyrrolidine-1-carboxylate (**2d)** as a colorless oil (31.83 mg, 69%).

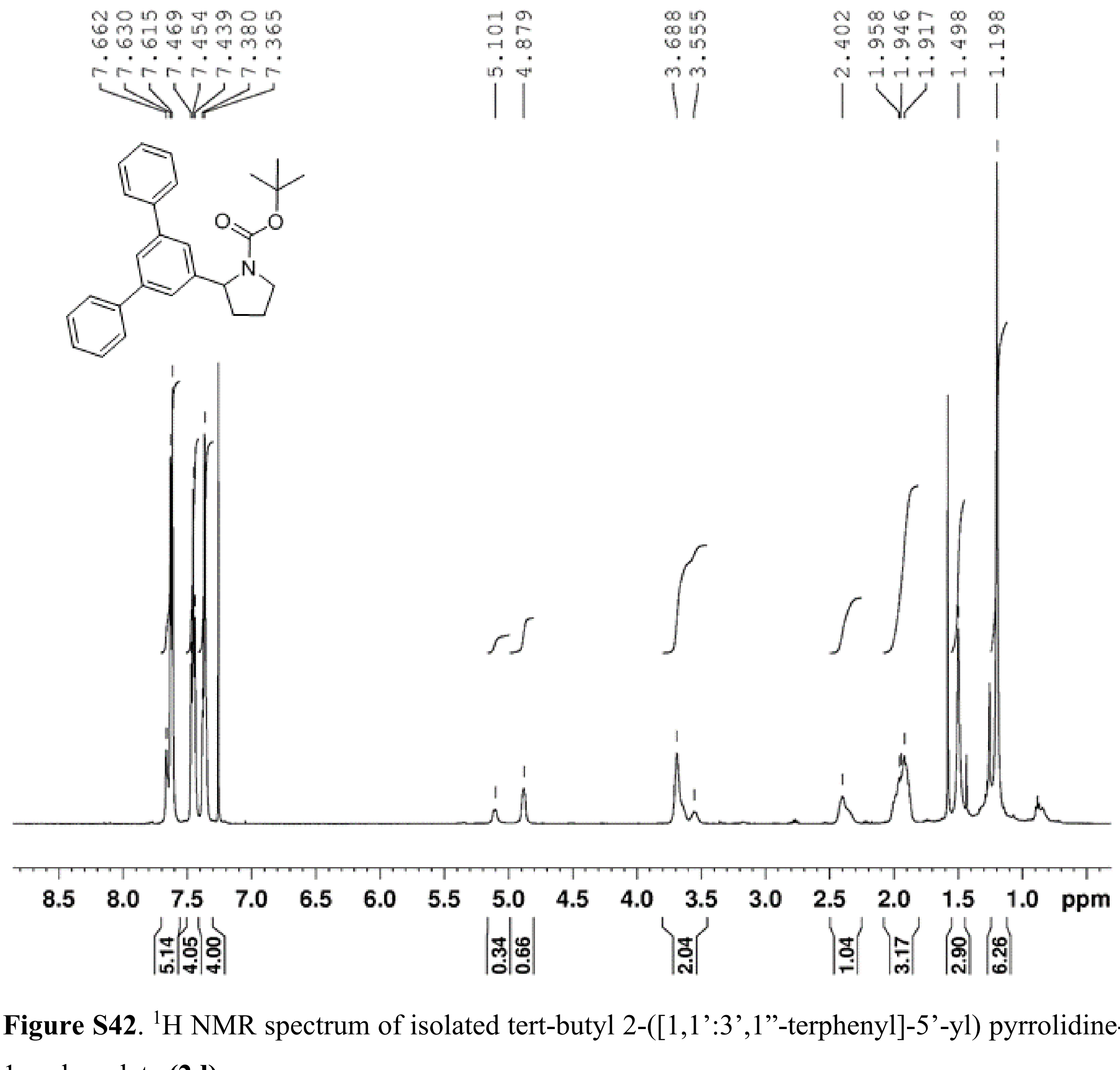

Figure S42. ^{1}H NMR spectrum of isolated tert-butyl 2-([1,1':3',1"-terphenyl]-5'-yl) pyrrolidine-1-carboxylate **(2d)**.

9.3 Recycling experiments for Fe(THF)$_2$•TPY-MOL catalyzed C_{sp}^3 C-H Amination

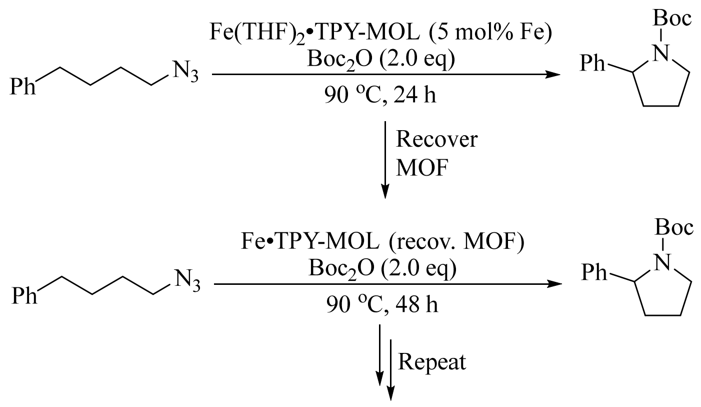

Run	Yield	Starting Material Remaining
**1**	100%	0%
**2**	60%	35%
**3**	59%	41%
**4**	53%	~50%
**5**	40%	~60%

Scheme S4. Recycling experiments for Fe(THF)$_2$•TPY-MOL catalyzed Csp^3 C-H Amination

Inside a nitrogen filled glovebox, Fe(THF)$_2$•TPY-MOL (0.68 µmol, suspended in 0.47 mL benzene) was added 1-azido-4-phenylbutane (**1a**) (2.38 mg, 13.6 µmol) dissolved in 0.1 mL of benzene. Boc$_2$O (6.2 µL, 27.2 µmol) was added, and the flask was sealed with a glass stopper. After removing the reaction flask from the glovebox, the sidearm was connected to a Schlenk line and subjected to vacuum then backfilled with N_2 (5x). The sidearm was then opened to positive N_2 flow [IMPORTANT as CO_2 is released throughout reaction], and the reaction flask was heated at 90 °C with gentle stirring for 48 h. After cooling to r.t., the flask was transferred back to the N_2-glovebox where the MOL was separated by centrifugation, and the product was extracted away from the MOL with THF washes (4x). The combined organic extracts were concentrated on a rotary evaporator. The residue was added mesitylene (1.89 µL) as an internal standard and the yield was determined by ^{1}H NMR in $CDCl_3$. Recovered Fe•TPY-MOL was transferred back into the reaction tube, and **1a** (2.38 mg, 14.2 µmol), benzene (0.57 mL), and Boc$_2$O (6.5 µL, 28.4 µmol)

were added. The reaction vessel was sealed and reactions repeated under the conditions as described above.

9.4 Heterogeneity test and cross-over experiments

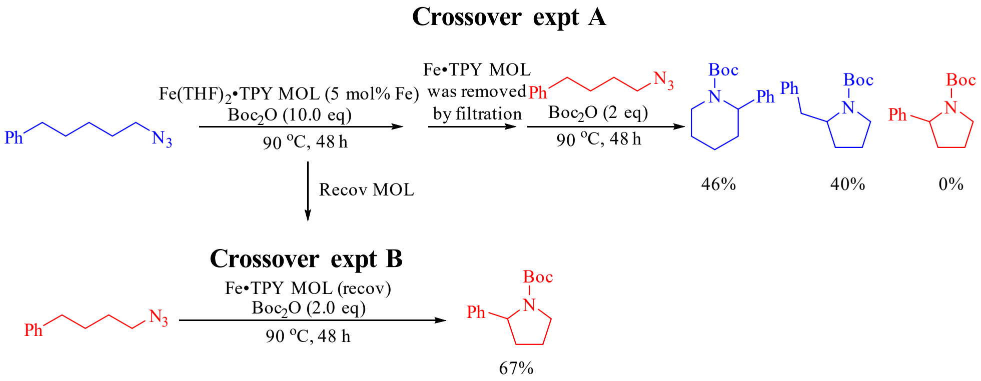

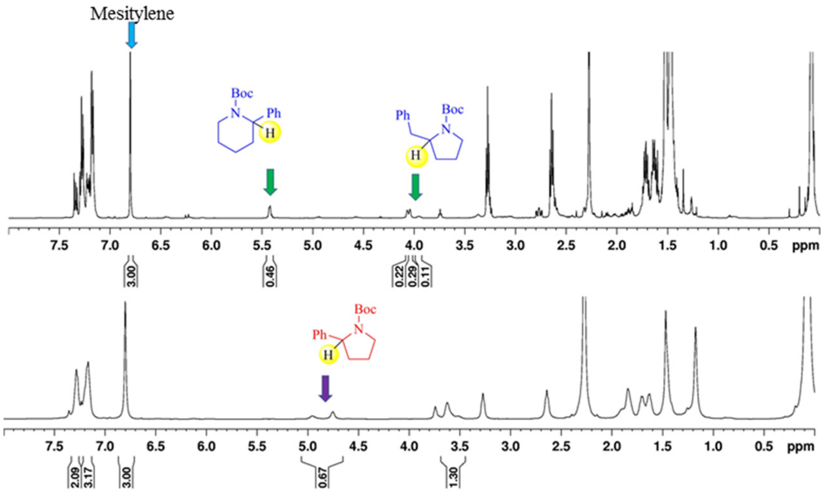

Inside a nitrogen filled glovebox, $Fe(THF)_2$•TPY-MOL (0.68 μmol, suspended in 0.47 mL benzene) was added to 1-azido-4-phenylpentane (**1a**) (2.57 mg, 13.6 μmol) dissolved in 0.1 mL benzene. Boc_2O (31.2 μL, 136 μmol) was added, and the flask was sealed with a glass stopper. After removing the reaction flask from the glovebox, the sidearm was connected to a Schlenk line and subjected to vacuum then backfilled with N_2 (5x). The sidearm was then opened to positive N_2 flow [IMPORTANT as CO_2 is released throughout reaction], and the reaction flask was heated at 90 °C with gentle stirring for 48 h. After cooling to r.t., the MOL was separated by centrifugation, and the supernatant was filtered through a short plug of Celite which was subsequently washed with 0.5 mL benzene. 1-azido-4-phenylbutane (**1a**) (2.38 mg, 13.6 μmol) and Boc_2O (6.2 μL, 27.2 μmol) were added to the supernatant, and the resulting solution was heated at 90 °C under N_2 protection for 48 h. The reaction mixture was concentrated in vacuo. The

resulting residue was added mesitylene as internal standard, and the yields of possible amination products were determined from ^{1}H NMR spectra taken in $CDCl_3$. After removal of the MOL, no product is formed from 1-azido-4-phenylbutane. Recovered Fe•TPY-MOL was transferred back into the reaction tube, and **1a** (2.38 mg, 14.2 μmol), benzene (0.57 mL), and Boc_2O (6.5 μL, 28.4 μmol) were added. The reaction vessel was sealed and reaction repeated under the conditions as described above. A yield of 65% was observed for 1-azido-4-phenylbutane, confirming the heterogeneous nature of the MOL catalyst.

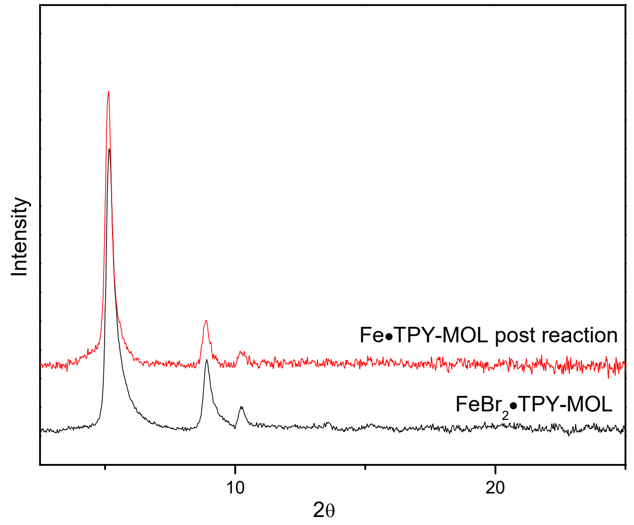

Figure S43. PXRD of Fe•TPY-MOL post reaction indicates the stability of MOL catalysts under reaction conditions.

Table S8. Comparison of the activity of $Fe(THF)_2$•TPY-MOL with previous reports[a]

Substrate	Product	Catalyst	Cat. Loading (mol%)	Yield	Reference
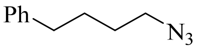	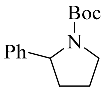	$Fe(THF)_2$•TPY-MOL	1	76%	this work
		[Fe]	10	57%	16
		[Pd]	10	10%	17
		Nacnac-MOF-Fe	5	90%	18
		[Fe-NNO]	0.1	62%[b]	19
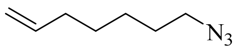	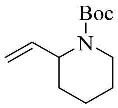	$Fe(THF)_2$•TPY-MOL	5	37%	this work
		[Fe]	100	45%	16
		[Fe-NNO]	5	38%[c]	19
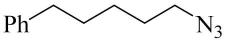		$Fe(THF)_2$•TPY-MOL	5	70% (1.0 : 0.9)	this work

	[Fe]	30	52 (1.0:0.9)	16

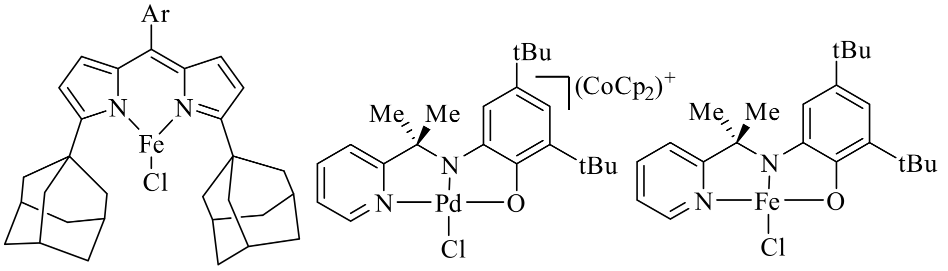

[Fe] [Pd-NNO] [Fe-NNO]

[a]The catalytic productivity does not necessarily reflect the catalytic activity of various catalysts. [b]Undesired Boc-protected amine (38%) was not observed in this work. [c]Undesired Boc-protected pyrrolidine (57%) was not observed in this work.

10. Density Functional Theory Calculation

Density functional theory (DFT) calculations were performed for Fe(tpy)X_2 and Co(tpy)X_2 systems with X=Cl, Br, or THF, using the Gaussian 09 software suite, version E01.[20] These complexes were optimized at the level of open-shell B3LYP/6-311G(d) theory. Tetrahydrofuran solvation was incorporated with the implicit solvent model, the conductor-like polarizable continuum model (CPCM).

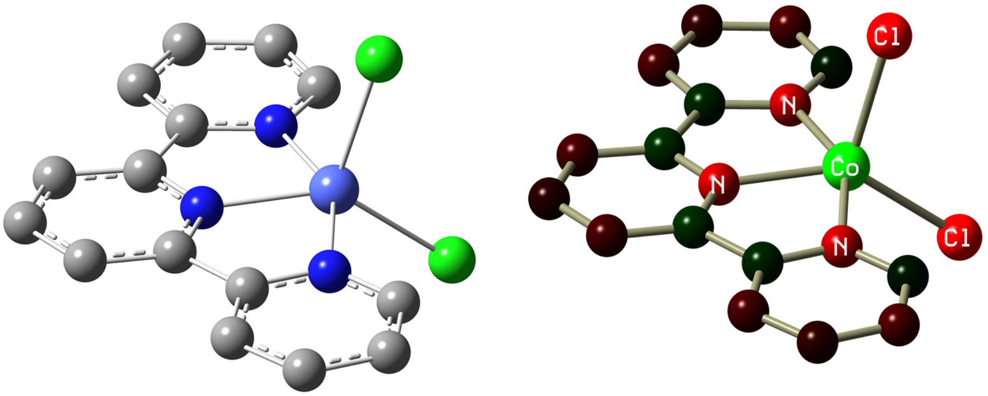

Figure S44. Optimized structure and calculated NBO charge distribution of Co(tpy)Cl_2. Positively charged and negatively charged atoms are denoted by green and red colors.

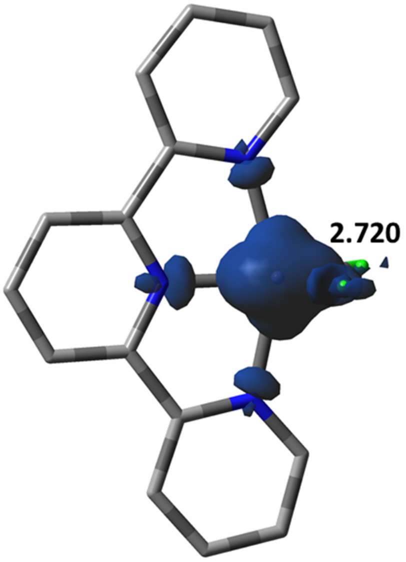

Figure S45.Spin density plot (blue: positive, green: negative) of Co(tpy)Cl_2.

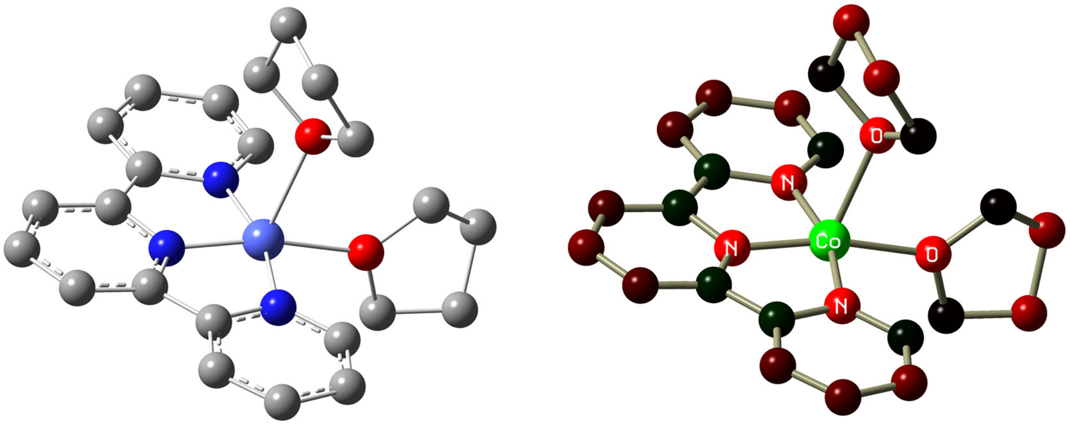

Figure S46. Optimized structure and calculated NBO charge distribution of Co(tpy)$(THF)_2$. Positively charged and negatively charged atoms are denoted by green and red colors.

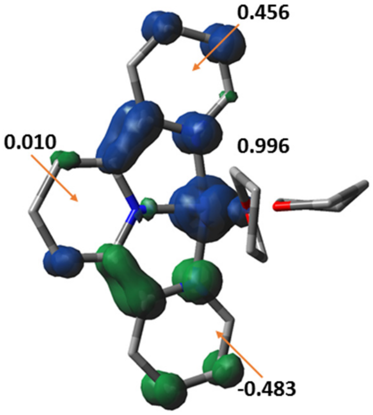

Figure S47.Spin density plot (blue: positive, green: negative) of Co(tpy)(THF)$_2$ (doublet GS).

Table S9. NBO charge population analysis of Co(tpy)Cl$_2$ and Co(tpy)(THF)$_2$

Fragment	Co(tpy)Cl$_2$	Co(tpy)(THF)$_2$_doublet	Co(tpy)(THF)$_2$_quartet
Co	1.45	1.24	1.29
tpy	0.200	-1.34	-1.40
X1	-0.824	0.030	0.080
X2	-0.823	0.071	0.030

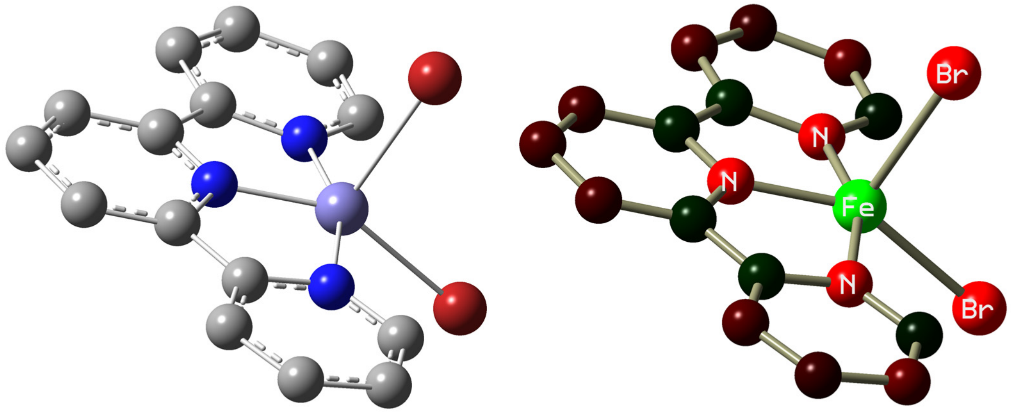

Figure S48. Optimized structure and calculated NBO charge distribution of Fe(tpy)Br$_2$. Positively charged and negatively charged atoms are denoted by green and red colors.

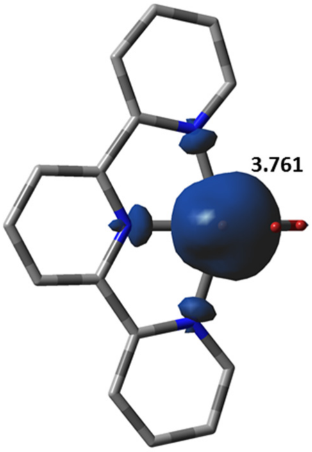

Figure S49. Spin density plot (blue: positive, green: negative) of Fe(tpy)Br$_2$.

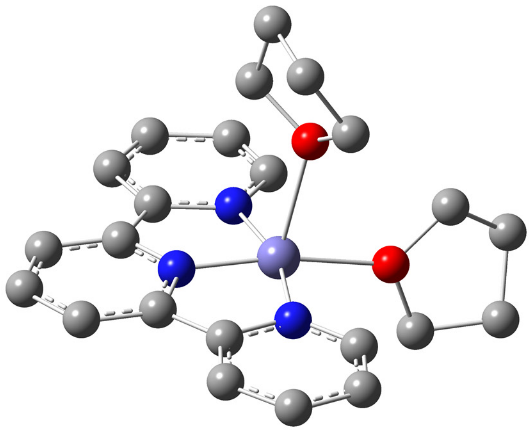

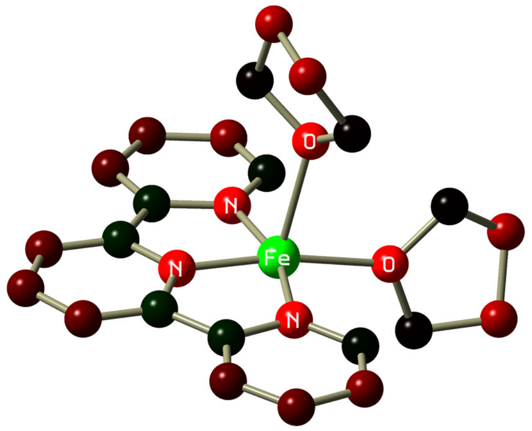

Figure S50. Optimized structure and calculated NBO charge distribution of Fe(tpy)$(THF)_2$. Positively charged and negatively charged atoms are denoted by green and red colors.

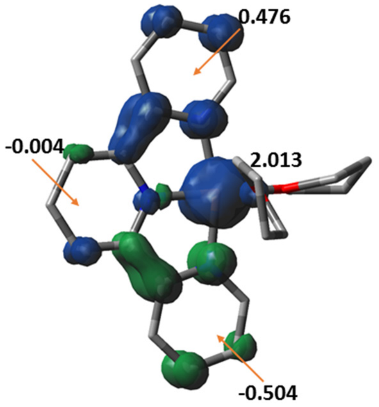

Figure S51.Spin density plot (blue: positive, green: negative) of Fe(tpy)$(THF)_2$ (triplet GS).

Table S10. NBO charge population analysis of Fe(tpy)Br_2, and Fe(tpy)$(THF)_2$.

Fragment	Fe(tpy)Br_2	Fe(tpy)$(THF)_2$_triplet	Fe(tpy)$(THF)_2$_quintet
Fe	1.48	1.29	1.34
tpy	0.174	-1.39	-1.44
X1	-0.839	0.026	0.028
X2	-0.816	0.068	0.074

Table S11. Selected DFT-calculated bond distances (Å) of complexes

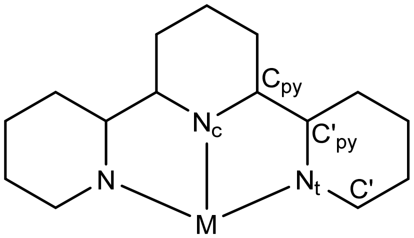

Complex	M-N_c	M-N_t	C_{py}-C'_{py}	N_c-C_{py}	N_t-C'_{py}	N_t-C'
$Co^{II}(tpy^0)Cl_2$	2.046	2.172	1.489	1.342	1.351	1.334
$Co^{II}(tpy^{2-})(THF)_2$	1.838[a]<br>1.856[b]	1.940[a]<br>1.953[b]	1.427[a]<br>1.423[b]	1.383[a]<br>1.371[b]	1.409[a]<br>1.411[b]	1.355[a]<br>1.346[b]
$Fe^{II}(tpy^0)Br_2$	2.149	2.202	1.487	1.340	1.352	1.336
$Fe^{II}(tpy^{2-})(THF)_2$	1.856[a]<br>1.885[b]	1.967[a]<br>1.992[b]	1.429[a]<br>1.425[b]	1.387[a]<br>1.373[b]	1.410[a]<br>1.412[b]	1.358[a]<br>1.348[b]

[a]$(tpy^{2-})^{2-}$ ($S = 0$) ground state. [b]$(tpy^{\bullet\bullet})^{2-}$ ($S = 1$) excited state

12. References

1. Sato, Y.; Nakayama, Y.; Yasuda, H. *J. Organometallic Chem.* **2004,** *689* (4), 744-750.
2. Kamata, K.; Suzuki, A.; Nakai, Y.; Nakazawa, H. *Organometallics* **2012,** *31* (10), 3825-3828.
3. Cao, L.; Lin, Z.; Peng, F.; Wang, W.; Huang, R.; Wang, C.; Yan, J.; Liang, J.; Zhang, Z.; Zhang, T.; Long, L.; Sun, J.; Lin, W. *Angew. Chem., Int. Ed.* **2016,** *55* (16), 4962-4966.
4. Rehr, J. J.; Albers, R. C. *Rev. Mod. Phys.* **2000,** *72* (3), 621-654.
5. Ravel, B.; Newville, M. *J. Synchrotron Rad.* : **2005**; 12, 537-541.
6. Stieber, S. C. E.; Milsmann, C.; Hoyt, J. M.; Turner, Z. R.; Finkelstein, K. D.; Wieghardt, K.; DeBeer, S.; Chirik, P. J. *Inorg. Chem.* **2012,** *51* (6), 3770-3785.
7. Léonard, N. G.; Bezdek, M. J.; Chirik, P. J. *Organometallics* **2017**, *36*, 142-150
8. England, J.; Scarborough, C. C.; Weyhermüller, T.; Sproules, S.; Wieghardt, K. *Eur. J. Inorg. Chem.* **2012,** 4605-4621
9. Ishiyama, T.; Ishida, K.; Takagi, J.; Miyaura, N. *Chem. Lett.* **2001**, 1082-1083.
10. Boebel, T. A.; Hartwig, J. F. *Organometallics* **2008**, *27*, 6013-6019.
11. Larsen,. M. A.; Wilson, C. V.; Hartwig, J. F. *J. Am. Chem. Soc.* **2015**, *137*, 8633-8643.
12. Manna, K.; Ji, P.; Lin, Z.; Greene, F. X.; Urban, A.; Thacker, N. C.; Lin, W. *Nat. Commun.* **2016**, *7*, 12610.
13. Shimada, S.; Batsanov, A. S.; Howard, J. A. K.; Marder, T. B. *Angew. Chem., Int. Ed.* **2001**, *40*, 2168-2171
14. E: Palmer, W. N.; Obligacion, J. V.; Pappas, I.; Chirik, P. J. *J. Am. Chem. Soc.* **2016**, *138*, 766-769.
15. F: Furukawa, T.; Tobisu, M.; Chatani, N. *Chem. Commun.* **2015**, *51*, 6508-6511.
16. Hennessy, E. T.; Betley, T. A. *Science* **2013,** *340* (6132), 591-595.
17. Broere, D. L. J.; de Bruin, B.; Reek, J. N. H.; Lutz, M.; Dechert, S.; van der Vlugt, J. I. *J. Am. Chem. Soc.* **2014**, *136*, 11574−11577.
18. Thacker, N. C.; Lin, Z.; Zhang, T.; Gilhula, J. C.; Abney, C. W.; Lin, W. *J. Am. Chem. Soc.* **2016**, *138*, 3501-3509

19. Bagh, B.; Broere, D. L. J.; Sinha, V.; Kuijpers, P. F.; van Leest, N. P.; de Bruin, B.; Demeshko, S.; Siegler, M. A.; Vlugt, J. I. D. *J. Amer. Chem. Soc.* **2017**, *139*, 5117
20. M. J. Frisch, G. W. Trucks, H. B. Schlegel, G. E. Scuseria, M. A. Robb, J. R. Cheeseman, G. Scalmani, V. Barone, B. Mennucci, G. A. Petersson, H. Nakatsuji, M. Caricato, X. Li, H. P. Hratchian, A. F. Izmaylov, J. Bloino, G. Zheng, J. L. Sonnenberg, M. Hada, M. Ehara, K. Toyota, R. Fukuda, J. Hasegawa, M. Ishida, T. Nakajima, Y. Honda, O. Kitao, H. Nakai, T. Vreven, J. A. Montgomery, Jr., J. E. Peralta, F. Ogliaro, M. Bearpark, J. J. Heyd, E. Brothers, K. N. Kudin, V. N. Staroverov, T. Keith, R. Kobayashi, J. Normand, K. Raghavachari, A. Rendell, J. C. Burant, S. S. Iyengar, J. Tomasi, M. Cossi, N. Rega, J. M. Millam, M. Klene, J. E. Knox, J. B. Cross, V. Bakken, C. Adamo, J. Jaramillo, R. Gomperts, R. E. Stratmann, O. Yazyev, A. J. Austin, R. Cammi, C. Pomelli, J. W. Ochterski, R. L. Martin, K. Morokuma, V. G. Zakrzewski, G. A. Voth, P. Salvador, J. J. Dannenberg, S. Dapprich, A. D. Daniels, O. Farkas, J. B. Foresman, J. V. Ortiz, J. Cioslowski, and D. J. Fox, Gaussian, Inc., Wallingford CT, 2013.
21. Sheldrick, G. M. *Acta Cryst.* **2015,** *C71*, 3-8.
22. Dolomanov, O. V.; Bourhis, L. J.; Gildea, R. J.; Howard, J. A. K.; Puschmann, H. *J. Appl. Cryst.* **2009,** *42*, 339-341.
23. Sheldrick, G. M. *Acta Cryst.* **2008,** *A64*, 112-122.
